# Integration Between Cerebral Hemispheres Contributes to Defense Mechanisms

**DOI:** 10.3389/fpsyg.2020.01534

**Published:** 2020-07-07

**Authors:** Sergio Paradiso, Warren S. Brown, John H. Porcerelli, Daniel Tranel, Ralph Adolphs, Lynn K. Paul

**Affiliations:** ^1^Institute of Cognitive and Translational Neuroscience, INECO Foundation, Favaloro University, CONICET, Buenos Aires, Argentina; ^2^Travis Research Institute, Fuller Graduate School of Psychology, Pasadena, CA, United States; ^3^Department of Psychology, University of Detroit Mercy, Detroit, MI, United States; ^4^Department of Neurology and Psychology and Neuroscience Program, The University of Iowa, Iowa City, IA, United States; ^5^Division of Biology, California Institute of Technology, Pasadena, CA, United States; ^6^Division of the Humanities and Social Sciences, California Institute of Technology, Pasadena, CA, United States; ^7^International Research Consortium for the Corpus Callosum and Cerebral Connectivity (IRC5), Pasadena, CA, United States

**Keywords:** corpus callosum, agenesis of the corpus callosum, defense mechanisms, connectivity, interhemispheric transfer

## Abstract

Defense mechanisms are mental functions which facilitate coping when real or imagined events challenge personal wishes, needs, and feelings. Whether defense mechanisms have a specific neural basis is unknown. The present research tested the hypothesis that interhemispheric integration plays a critical role in defense mechanism development, by studying a unique sample of patients born without the corpus callosum (agenesis of the corpus callosum; AgCC). Adults with AgCC (*N* = 27) and matched healthy volunteers (*N* = 30) were compared on defense mechanism use across increasing levels of developmental maturity (denial, least; projection, intermediate; identification, most). Narratives generated in response to Thematic Apperception Test images were scored according to the Defense Mechanism Manual. Greater use of denial and less identification was found in persons with AgCC, compared to healthy comparisons. This difference emerged after age 18 when full maturation of defenses among healthy individuals was expected. The findings provide clinically important characterization of social and emotional processing in persons with AgCC. More broadly, the results support the hypothesis that functional integration across the hemispheres is important for the development of defense mechanisms.

## Introduction

Defense mechanisms have critical importance in everyday life and in psychopathology (Fenichel, 1946; Lowenstein et al., 1975; Wallerstein, 1983; Lampl-de Groot, 1985; Cramer, 2006) playing a substantive role in the comprehensive understanding and managing of psychiatric illness (Handbook of Psychiatric Measures, 2008). Assessing defense mechanisms affords diagnostic and prognostic information on development and psychopathology (Vaillant, 2011). Besides giving an important indication of an individual’s level of adaptation, defense mechanisms are strong predictors of psychological adjustment after many decades of life (Vaillant, 1994) and are amenable to change from less to more mature after successful dynamic psychotherapy (Albucher et al., 1998).

The DSM-5 describes defenses as “Mechanisms that mediate the individual’s reaction to emotional conflicts and external stressors” (American Psychiatric Association, 2013, p. 819). Following the insights of his early clinical work, Sigmund Freud identified a force directed to modulate or stop (repress) unacceptable impulses that would otherwise provoke overwhelming anxiety (Freud and Breuer, 1895). Repression, the first defense Freud included in his monumental work that laid the foundations of psychoanalysis, begins early in life. When a child finds out that acting on some desires may give raise to anxiety, this powerful emotion may set in motion repression of the desire. Mechanisms similar to repression were noted earlier by Arthur Schopenhauer and Johann Friedrich Herbart (psychologist and founder of academic pedagogy, 1776–1841), but it was Freud who established repression (in German *Verdrängung*) as the cornerstone of psychoanalysis. Later on, his daughter Anna discussed and clarified the various types of defenses (10 or more) that appeared throughout her father’s publications (Freud, 1936).

More than 40 different defenses have been described to date (Bibring et al., 1961) substantially broadening Freud’s early indications. For instance, recent emphasis has been placed on interpersonal factors in defense use and development. A child who finds out that a significant adult caregiver lacks empathy may “fail” to acknowledge this lack to keep the original feeling toward the adult. According to Kohut (1977) defense mechanisms have thus an additional function: to protect from disappointment of empathic failures of adults, therefore protecting the self and supporting self-esteem (Cramer, 2006). In summary, defense mechanisms are mental functions that address percepts originating within the self or in the external world that clash with personal wishes and feelings or are inconsistent with overt or covert opinions about the self.

As neuroscientists, we presume that brain structure and function has a critical impact on all mental functions, including defense mechanisms. To date, attempts to relate modern neurology and psychodynamic theory have focused on defining (or re-defining) neurological symptoms within a psychodynamic framework. For example, anosognosia (i.e., denial of deficit/illness) occurring after right hemisphere damage has been interpreted as a manifestation of difficulty handling separation and loss (tolerate negative emotions) and interpreted psychodynamically as denial of personal limitations (physical as well as emotional) to limit the emotional consequences of catastrophic change (e.g., Kaplan-Solms and Solms, 2000; Turnbull et al., 2002, 2005, 2014). In contrast, the present study directly assesses use of defense mechanisms in a population with a known neuroanatomic malformation.

Defense mechanisms have been posited to lay on complex cognitive-emotional interactions (Westen and Gabbard, 2002a, b) requiring coordinated functioning of multiple anatomically distinct brain regions (i.e., large scale neuronal integration) (Northoff and Boeker, 2006; Northoff et al., 2007). This view forms the background of the present study. In the present research we tested a major aspect of neuroanatomical integration, that is integration between the two cerebral hemispheres. The necessity of intact interhemispheric connectivity via the corpus callosum for development of defense mechanisms was tested by examining the use of defenses in a rare sample of individuals with congenital absence of the corpus callosum (AgCC). Absence of the corpus callosum is congenital in this sample, therefore the present study tests the critical importance of this brain structure in the development of defenses.

The human corpus callosum with its ∼200 million axons transferring information between the two brain hemispheres (Tomasch, 1954) is the largest fiber tract in the human brain. Individuals with AgCC have complete or partial absence of the corpus callosum. A 20-year study of birth-defects registries in California found that AgCC occurred 1 in 4,000 births (Glass et al., 2008). Although AgCC is associated with multiple syndromes of brain malformation related to known toxic-metabolic conditions or genetic causes, in 55–70% of cases the cause of AgCC is unknown (Bedeschi et al., 2006; Schell-Apacik et al., 2008; Tang et al., 2009, for a review see Chiappedi and Bejor, 2010). Although it can co-occur with other brain malformations and genetic conditions, AgCC can be an isolated neuroanatomical finding (Paul et al., 2007). When AgCC is accompanied by other brain malformations or occurs in the context of broader neurodevelopmental syndromes, these additional factors typically dominate the behavioral outcomes. However, when AgCC is the primary neurological finding (i.e., without any other or only very minor other brain dysmorphology and no known developmental syndrome), it is more common to see globally intact intellectual abilities with relatively subtle limitations (Chiappedi et al., 2012; Brown and Paul, 2019). The present research is based on examination of individuals for whom AgCC appears to be the primary neurodevelopmental complication, affording the rare opportunity to test the role of congenital disruption of interhemispheric connectivity in the development of defenses.

Back in the 70’s, Klaus Hoppe envisioned that a systematic examination of individuals who underwent surgical callosotomy to alleviate intractable seizures might give insights into the understanding of the brain bases of psychoanalytic theories (Hoppe, 1977). This line of research gave rise to a series of studies on “split brain” patients who displayed a disconnection syndrome characterized by absent callosal transfer of sensory information and deficient bimanual motor control (Sperry, 1968; Sperry et al., 1969; Bogen and Frederiks, 1985). These patients also exhibited alexithymia (see for instance TenHouten et al., 1985a, b), a pattern of poor awareness for their emotional processes and limited capacity to verbalize them (Taylor et al., 1997). The association between callosal function and alexithymia has been further born out in subsequent studies of individuals with AgCC (Buchanan et al., 1980; Paul et al., 2007). Although individuals with AgCC do not display the full disconnection syndrome seen in split-brain patients, they do exhibit diminished interhemispheric transfer compared to controls (for review see Paul et al., 2007). Connection between alexithymia and immature defense mechanisms (Helmes et al., 2008; Besharat and Khajavi, 2013) offer additional support for the general hypothesis that functional integration between the two hemispheres plays a critical role in development and implementation of defense mechanisms.

For the present report we focus on three widely studied and developmentally anchored defense mechanisms: denial, projection, and identification. Because defenses often take place below the level of awareness, we chose an observational method of measurement over a self-report one. An observational method gives people free rein to speak personal thoughts and feelings while these are recorded for coding. A commonly used method to elicit a narrative from participants, the Thematic Apperception Test, was used (TAT; Murray, 1943) together with the Defense Mechanism Manual (DMM) for coding (Cramer, 1991a, 2007; Porcerelli et al., 1998). This approach yields the above three defense categories (each of which is composed of seven subscales). Other widely used methods (Defense Mechanism Rating Scales, Perry, 1990; Defense Style Questionnaire, Andrews et al., 1993) yield a larger number of defenses that in research studies are grouped into a smaller number of defense categories. Factor analytic studies have repeatedly shown a three-factor solution with dimensions similar to those obtained with the DMM (for an in-depth discussion see Cramer, 2006, pp. 15–19). Here follows a brief explanation of the defense categories used in this research. Denial aims at minimizing tension by warding off internal or external percepts that if acknowledged could be potentially upsetting. Projection aims to minimize tension by (mis)attributing to another person unacceptable thoughts, feelings or impulses that originate in the self. Identification aims at minimizing tension by taking on behaviors, qualities or attitudes of another person and experiencing them as part of the self (Cramer, 2006). Defenses are linked to developmental stages (see below). They can also be distinguished based on complexities of the mental operation involved. When it comes to denial the operations comprising it are broadly of two types. A majority are closely related to the perceptual system (not seeing or distorting what is perceived) while others involve the construction of a personal fantasy or an enacted dream (Freud, 1936) distorting reality and replacing a significant part of the experience. Because of this reliance on the perceptual system (the system that “provides the first bridge to the outside world,” Cramer, 2006, p. 44) denial is considered most primitive. In the mechanism of projection, the perceptual phase is largely unaffected, as is the capacity to make hedonic judgments on percepts. Various degrees of alteration of the capacity of attribution of these to the internal or the external world are present. The greater complexity relative to denial is apparent. Identification is yet more complex a defense. Whereas identification has been described as comprising multiple components (incorporation, introjection and identification proper; Meissner, 1974) which may include aspects of incomplete differentiation between subject and object, at the far end of this continuum (identification proper) lays a mature capacity to distinguish the other person as separate. Notably the present research reports on identification as a defense (i.e., the mental mechanism aiming at avoiding anxiety and keeping self-esteem). *Developmental* identification is a process in individual maturation serving the purpose of becoming independent and autonomous from significant others. In identification as a defense the prospected or actual loss of a significant other is handled by recreating an internal object as a replica of the lost one. In addition, partial aspects of a significant other may be included in the ego such as moral standards in order to keep parents’ approval or reduce unacceptable drives and impulses.

Emergence of defenses begins in childhood and continues during adolescence (Cramer, 1987, 1997; Porcerelli et al., 1998) mapping onto the phases of corpus callosum development. A period of rapid callosal myelination occurs during the second year of life (Morriss et al., 1999) just prior to the peak use of denial beginning around age 2 (Cramer, 1987, 1997; Porcerelli et al., 1998). In the period between ages 2 and 12, the anterior callosum grows substantially (Luders et al., 2010) while the use of denial declines and use of projection peaks (Cramer, 1987, 1997; Porcerelli et al., 1998). Finally, the posterior callosum begins a period of substantial growth and myelination around age 9–12 (Ballmaier et al., 2008; Lebel et al., 2008; Knyazeva, 2013) just as identification enters the repertoire of defense mechanisms (Cramer, 1987, 1997; Porcerelli et al., 1998). Identification is the defense mechanism typical of adolescence and early adulthood and is considered the most adaptive of the three types of mechanisms (Cramer, 1987, 1997; Porcerelli et al., 1998). It should be clarified that defenses in general and in particular those examined in the present research differ by age exclusively in their expected “peaks” of relative frequency of occurrence. This means that there is plenty of overlap among defenses during normal childhood, adolescence and early adulthood (Cramer, 1991b).

Previous studies of individuals with AgCC utilized stories elicited in response to the picture stimuli from the Thematic Apperception Test (TAT; Murray, 1943) to gain insights regarding aspects of cognitive and emotional functioning. A small-sample study of five adults with AgCC and age-matched controls first reported that the stories told by individuals with AgCC were impoverished in story logic, social understanding, and inclusion of common content (Paul et al., 2004). This was followed by linguistic analysis of stories told by 22 individuals with AgCC (Turk et al., 2010). Compared to stories told by age- and IQ-matched controls, the stories told by individuals with AgCC in this study were far more variable in length and tended to include more words overall. Relative to total words per story, individuals with AgCC used fewer words pertaining to emotionality, cognitive processes, and social processes, but they used relatively more present-tense verbs and first person pronouns (Turk et al., 2010). These results suggest that callosal agenesis might interfere with the ability to picture social interactions and the mental processes of others. No study to date has formally examined defense mechanisms in this rare population.

The general hypothesis set forth in the present study was that progression from immature (denial) to more mature (identification) defenses requires optimal functioning of brain structures devoted to information integration. Individuals with AgCC were expected to show greater utilization of immature defenses (denial) and less utilization of mature defenses (identification) relative to sex-, age- and IQ-matched healthy participants. In addition, based on defense mechanisms developmental data (Cramer, 1987, 1997; Porcerelli et al., 1998), age was expected to be significantly negatively correlated with denial and significantly positively correlated with identification among healthy participants, whereas among participants with AgCC, such associations were predicted to be absent. Finally, we expected that relative compensation for immature defense responses could arise in individuals with AgCC and relatively higher intellect, leading to the prediction that in individuals with AgCC, intelligence would be significantly negatively correlated with denial and significantly positively correlated with more mature defenses.

## Materials and Methods

### Participants

Participants included thirty individuals with agenesis of the corpus callosum (20 men) and 30 healthy comparison individuals (23 men) (Table 1). Inclusion criteria for all participants were the following: full scale intelligence quotient (FSIQ) of 80 or greater, native English speaking, and participation in mainstream education. Exclusion criteria were history of major head trauma, neurosurgery or central nervous system disease (agenesis of the corpus callosum excluded), more than two life-time seizures, diagnosis of schizophrenia or other psychotic disorder, or diagnosis of bipolar disorder.

**TABLE 1:** Demographics, cognitive scores and relative defense scores.

	AgCC (*n* = 27)			Comparison (*n* = 30)		
	Mean	*SD*	Range	Mean	*SD*	Range
Age	19.26	11.72	7–56	19.00	11.00	8–51
FSIQ	97.52	10.58	83–122	95.43	7.27	84–120
+PIQ	98.85	11.66	78–120	97.12	8.18	78–109
+VIQ	98.38	15.00	76–140	93.18	6.48	84–109
Mean words per story	135.72	150.20	8.83–619.67	88.42	42.38	21.3–204.3
Mean queries per story	1.99	1.56	0–6.33	1.78	1.15	0–4
RDS Denial*	0.53	0.22	0.17–1	0.41	0.19	0–0.72
RDS Projection	0.34	0.18	0–0.78	0.34	0.12	0.09–0.56
RDS Identification**	0.13	0.17	0–0.6	0.25	0.19	0–0.7
Gender	10F: 17M			7F: 23M		
Handedness	7L: 20R			3L: 27R		

*p < 0.05, **p < 0.005, + AgCC *n* = 29 and Comparison *n* = 17; SD, standard deviation; FSIQ, Full Scale Intelligence Quotient; PIQ, Performance Intelligence Quotient; VIQ, Verbal Intelligence Quotient.

AgCC was confirmed by MRI in all participants but one who received a CT scan. MRI was acquired at Caltech for 11 participants, at UCSF for 1 participant, and in clinical settings for the remainder. We were able to review 26 of the MRI scans (including all 4 partial AgCC participants) and reviewed clinical imaging reports for the remaining four participants. The size of residual CC for each participant with partial AgCC was less than <10% of typical CC size (based on visual inspection of a midline sagittal MRI image by two raters). Individuals with AgCC were excluded if they had other major brain abnormalities detected on MRI (other than colpocephaly or small interhemispheric cysts, both of which frequently accompany complete AgCC). Three participants with AgCC were excluded from analysis due to presence of additional neuropathology (one with bilateral heterotopia, one with global dysgenesis of left hemisphere, and one with bilateral dysgenesis of frontal lobes). Of the remaining 27 participants, 23 had complete AgCC, and 4 had partial AgCC. In direct review of MRI scans (23 of the remaining 27 participants), the anterior and posterior commissures were visible on 20 scans. Probst bundles were visible bilaterally in 18 participants with complete AgCC and 3 with partial AgCC. Probst bundles are white matter tracts that form during neurodevelopment when axons that would typically form the corpus callosum are unable to cross the midline and instead they form aberrant bundles along the interhemispheric fissure from the anterior to the posterior aspects of the brain in complete AgCC (in partial AgCC they begin at the posterior end of the callosal remnant and end in posterior aspects of the brain).

From the final sample, 26 AgCC participants received the age-appropriate Wechsler Intelligence test (Wechsler Adult Intelligence Scale-III or Wechsler Intelligence Scale for Children-III) and one received the Stanford-Binet. Two AgCC participants were taking anti-epileptic medications (valproic acid, or phenytoin) at the time of testing. Eighteen participants with AgCC were included in a previous study by Turk et al. (2010) and three were included in the study by Paul et al. (2004).

Healthy participants were recruited from community college psychology courses and from local employment agencies or were selected from elementary school populations in order to equate FSIQ and age to that of participants with AgCC. Verbal intelligence quotient (VIQ) and performance intelligence quotient (PIQ) were not available for thirteen healthy participants who were recruited from local private elementary schools (school psychologist provided FSIQ scores). All other comparison participants received the Wechsler Abbreviated Scales of Intelligence. Groups were matched on age [*t*(55) = 0.09, *p* = 0.93], FSIQ [*t*(55) = 0.87, *p* = 0.39], VIQ [*t*(41) = 1.35, *p* = 0.19], PIQ [*t*(41) = 0.53, *p* = 0.60], sex [Fisher’s Exact Test = 0.39] and handedness [Fisher’s Exact Test = 0.17].

Handedness was measured using the Edinburgh Handedness Inventory (range −100 to 100; Oldfield, 1971) with handedness categorization determined as in the original article (< −40 = left-handed, −40 to 40 = Ambidextrous, >40 = right-handed).

### Procedures

Defense mechanisms were measured from archival data acquired at the Travis Research Institute. Data collection methods and procedures were reviewed and approved by the Human Subjects Review Committee at the Travis Research Institute and participants were treated in accordance with the American Psychiatric Association (APA) Ethical Principles and provided informed consented to participate. Defense mechanisms measurement and analyses reported in this paper dataset were conducted with approval of the Caltech Institutional Review Board.

In light of the rarity of individuals with AgCC, and the lack of any prior effect size estimate, we made an *a priori* decision to fix our sample size at the maximum available, a conservative, common and accepted approach especially with clinical samples. Following participant exclusions described above, the final dataset included 57 transcripts (30 HC, 27 AgCC), each of which included the stories generated by participants in response to six pictures: 1, 2, 6BM, 8BM, 12 MF, 13MF of the TAT (Murray, 1943). Use of 2–6 TAT cards is standard in the psychological and psychiatric literature (Keiser and Prather, 1990; Porcerelli and Sandler, 1995). Researchers at Travis Research Institute utilized these six cards because of their potential to elicit social inference and emotional expressiveness in narrative responses and because they are frequently used in clinical practice. The TAT is a free-response (formerly known as “projective”) test widely used to measure defenses from stories generated in response to black and white drawings depicting various interpersonal scenarios. The thought processes of participants are inferred from relatively extended samples of thought (stories) stimulated by open-ended questions (i.e., thought content is used to infer thought processes or defenses) (Cramer, 1991a, 2007; Porcerelli et al., 1998).

According to the standard TAT administration protocol, participants were shown the cards one at a time and asked to tell a story for each card. Specifically the administrator said, “For the next test, I’m going to sit next to you. I am going to show you a series of pictures. For each picture, I want you to tell me a story with a beginning, middle, and end. Tell me what the characters are thinking, feeling, and doing. And make sure you tell me how it ends.” The examiner wrote down the stories verbatim. When the participant indicated that the story was finished, standardized queries were used to prompt for any of the six elements (beginning, middle, end, thinking, feeling, and doing) that had not already been spontaneously provided (e.g., “What are the characters thinking?” or “How does the story end?”). The TAT was administered by trained technicians, who wrote down the verbatim responses of the participants.

One of us, a psychiatrist (diplomat of the American Board of Psychiatry and Neurology) with extensive training in psychodynamics (SP), who was blind to participant group, and to demographic and clinical characteristics of the participants, scored the 60 randomly ordered transcripts using the Defense Mechanism Manual (DMM) (Cramer, 1991a, 2007; Porcerelli et al., 1998). To confirm that the rater was implementing the rating system accurately, fifteen randomly selected transcripts (7 from the AgCC group and 8 from HC group; 25% of the original dataset) were also scored by a board-certified psychologist (JHP) with 17 years of experience with the DMM, who was blinded to the diagnosis, demographic and clinical characteristics of participants. Inter-rater reliability for this subset was excellent [denial: ICC = 0.77 (0.87 corrected for double coding), projection = 0.76 (0.86 corrected for double coding), identification = 0.81 (0.90 corrected for double coding)].

The DMM is a detailed system for scoring stories elicited by TAT cards. DMM scoring produces frequency scores for denial, projection and identification. Each of these defense scores is comprised of the sum from 7 sub-categories reflecting a different aspect of the defense (for denial sub-categories include omission, misperception, reversal, statements of negation, denial of reality, overly maximizing positive, minimizing negative, unexpected goodness, optimism, positiveness, gentleness). Direct repetitions in a story are only scored once. See Table 2 for an example of a scored narrative from a participant with AgCC and a healthy participant. Groups did not differ for average number of words per story, *U* = 395, *p* = 0.87, or average number of queries per story, *U* = 386.5, *p* = 0.77 (two-tailed).

**TABLE 2:** Sample TAT stories.

Healthy participant	AgCC
TAT Card 2: Farm	TAT Card 2: Farm
S43: This summer day and the whole family is out on the farm. The husband is working the fields and mom is pregnant relaxing in the sun and the daughter is going to sit under a tree and read because it is a nice day. At the end of the day they sit at the dinner table and talk about how nice a day it was. They all feel joyful and happy because it is a nice clear day and they are all relaxing around the farm.	S39: This girl is coming home from school and she sees her mother watching her son work on the field like plant, using a plow behind the horse having it plow the fields and stuff like that and the girl goes and tells her mother that she is going to go do some homework and her mother does not respond, she is just staring off into space, looking at sky and sun and rest of field and the girl just walks off looking at her mother, wondering why she did not respond to her when she said she was going to do homework and after she gets back to the house, the other family members come back, too, and the mother and daughter sit down and talk. The daughter is basically doing small talk because she is not happy about something she was not allowed to do the night before. And they resolve their differences.
D: None	D1: Omission D7: Unexpected goodness, optimism…
P: None	P1: Aggressive, hostile feelings
I: None	I3: Regulation of motives, behavior

*D, Denial; P, Projection; I, Identification.*

Validity of the DMM has been addressed through experimental studies of child and adult responses to stress, correlational studies with personality and psychopathology, and longitudinal studies (including treatment studies) of changes in defense over time (Cramer, 2006).

### Data Analysis

**FIGURE 1:**
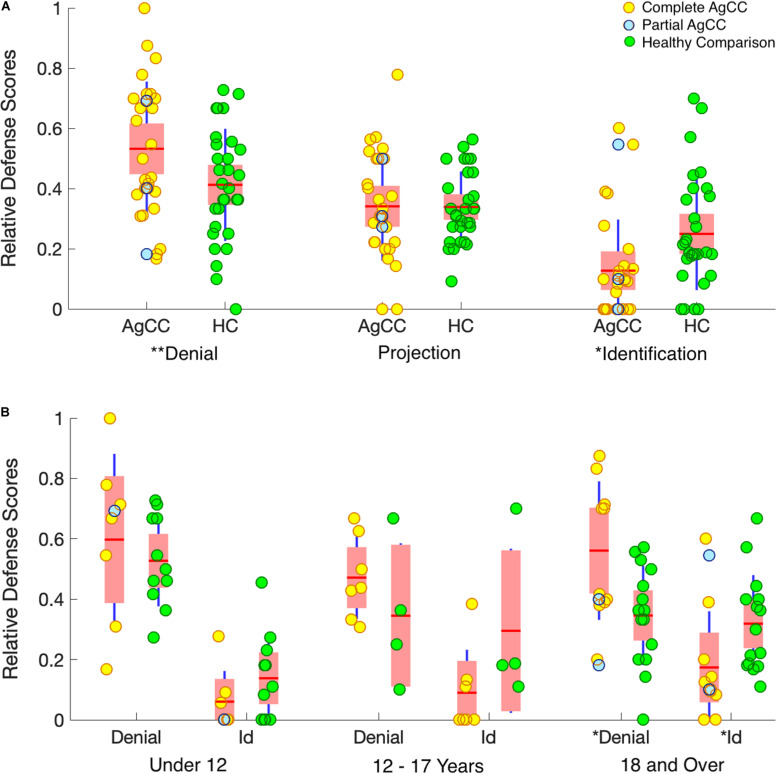
Relative defense scores. **(A)** Results by group. **(B)** Results by group and age range (under 12, 12–17, 18, and over). Results from individual participants, complete AgCC (yellow circles), partial AgCC (blue circles), and healthy participants (green circles), are overlaid onto boxplots of group statistics. Group means are indicated by horizontal red lines. On each boxplot, the wider pink area represents standard error of the mean (95% confidence interval) and the additional dark-blue vertical lines indicate the standard deviation. *p < 0.05 and **p < 0.1.

To control for individual differences in overall responsiveness to the TAT stimuli, each participant’s defense score was normalized into a relative defense score (RDS). RDS was calculated by dividing the count for each category of defenses by the participant’s total defense count across the three categories. RDS scores for all three defenses are included in Figure 1 and Tables 1, 3.

**TABLE 3:** Rank-order correlations of Relative Defense Scores with age, FSIQ, VIQ and words per story (two-tailed p-values for Projection).

Covariate	Age		FSIQ		VIQ		Words per story	
	τ	*p*	τ	*p*	τ	*p*	T	*p*
**AgCC**					*n = 26*			

Denial	–0.08	0.29	–0.22	0.059	–0.31	**0.016**	–0.32	0.10
Projection	–0.13	0.36	0.13	0.38	0.20	0.17	0.17	0.90
Identification	0.27	**0.031**	0.22	0.070	0.16	0.14	0.40	**0.003**

**Healthy volunteers**					*n = 17*			

Denial	–0.24	**0.035**	0.13	0.17	0.03	0.47	–0.13	0.31
Projection	–0.05	0.73	–0.15	0.28	0.04	0.87	0.17	0.18
Identification	0.33	**0.007**	–0.13	0.18	–0.18	0.17	0.11	0.20

*τ, Kendall’s tau; *r*, Spearman rank partial correlation; FSIQ, Full Scale Intelligence Quotient; VIQ, Verbal Intelligence Quotient. Bold p-values are significant at *p* < 0.05.*

Our approach to statistical analysis generally used parametric tests in the first instance, for more complex and factorial designs, followed by post-hoc tests that were non-parametric. Our reasons for this are as follows. First, parametric tests, such as ANOVA, have very well understood properties, and moreover are generally quite robust to modest violations in the assumptions of the distribution of the data. Second, parametric tests generally have greater statistical power than do non-parametric tests. By contrast, non-parametric tests make fewer distributional assumptions, but at the cost of lower statistical power. However, a clear benefit of non-parametric tests is that they consequently yield fewer false positives (i.e., they are more conservative in this sense). Thus, our approach used parametric tests first to discover possible effects with the greatest statistical power, followed by non-parametric tests to test specific contrasts in the most conservative fashion.

To directly address our *a priori* hypotheses regarding the defenses most markedly influenced by maturity level (denial and identification), we conducted a 2 × 2 ANOVA (subject group × defense type – denial vs. identification). Although variance was homogeneous across groups, RDS Identification scores in the AgCC group violated the normal-distribution assumption of the ANOVA. Nonetheless, we applied the ANOVA to this data because evidence from simulation studies have shown that the false positive rate of ANOVA is not markedly affected by non-normal distributions (Glass et al., 1972; Harwell et al., 1992; Lix et al., 1996) and it is the most direct measure of our hypothesis. To address concerns about non-normality, non-parametric tests (Mann–Whitney *U*) were used for between-group *post hoc* comparisons of RDS scores. Kendall rank (tau, “τ”) and Spearman rank partial correlation were used for all correlations. Since our data and its scoring also produced projection scores, we report these for readers’ interest only – but they do not constitute our main hypothesis that we wished to test.

Based on the clear directionality of the hypotheses [e.g., individuals with AgCC were expected to show greater utilization of immature defenses (denial) and less utilization of mature defenses (identification)], all direct analyses of these measures were one-tailed unless otherwise indicated. In contrast, two-tailed tests were used for analyses where directionality could not be predicted based on the literature (i.e., projection RDS scores).

To understand the association between age and defense mechanisms in each subject group, and how they might differ between the groups, we examined RDS scores in relation to age for each group, as well as the correlation of age with a combined defense metric (RDS identification divided by RDS denial). Finally, we conducted specific pairwise tests between subject groups broken down by age, in order to confirm that the predicted effects held across all ages. This involved a 2 × 2 ANOVA (subject group × defense type) conducted in three age ranges: under 12 when denial and projection are more prominent (Cramer, 1997), the period from age 12 through 17 when use of identification increases (Cramer, 2007), and age 18 onward when defense use has stabilized. To examine the possibility that intellectual ability might influence compensation for immature defense responses in individuals with AgCC, we correlated RDS scores with IQ scores in both groups.

RDS for the partial AgCC participants were evenly distributed in the rank order for all defense categories (Figure 1A). Partial and complete AgCC groups did not differ for means or ranks on RDS categories, justifying our pooling of these participants into a single AgCC group. Additionally, because there were no significant sex differences in defense mechanisms in the entire sample, nor in either group, we did not further examine sex as a variable (full sample: RDS Denial *U* = 299.5, *p* = 0.480; RDS Projection *U* = 321.5, *p* = 0.746; RDS Identification = 317.5, *p* = 0.692; two-tailed tests).

## Results

AgCC and healthy participants groups did not differ for total number of defenses used overall, *U* = 357, *p* = 0.57 (two-tailed), or for number of defenses per each of the six cards. This suggests that groups were equally engaged in the task. The 2 × 2 ANOVA of defense type (denial/identification) by subject group showed a significant interaction term, *F*(1,55) = 6.64, *p* = 0.013, $\eta_{\text{p}}^{2}$ = 0.108 (Figure 1A) and a significantly lower use of identification overall, *F*(1,55) = 36.58, *p* < 0.001, $\eta_{\text{p}}^{2}$ = 0.399, but no overall difference between group, *F*(1,55) = 0.003, *p* = 0.954, $\eta_{\text{p}}^{2}$ < 0.0000. *Post hoc* comparisons conducted to examine the interaction effect, found that participants with AgCC were more likely than healthy participants to use denial, *U* = 289, *p* = 0.032, and were less likely to use identification, *U* = 218, *p* = 0.002. Groups did not differ in use of projection, *U* = 404, *p* = 0.987.

We re-analyzed the data excluding participants who were currently taking antiepileptic drugs (*N* = 2) and re-analyzed the data excluding participants with partial AgCC (*N* = 4). For both analyses, the pattern of results for remained consistent with the original findings and effect size changes were minor. Because none of the subgroup participants’ scores were outliers, excluding them did not change the overall findings, and we did not have any *a priori* hypothesis about such group differences, they were pooled in all subsequent analyses.

In the AgCC and in the healthy group, age was positively correlated with identification. In the healthy group age was negatively correlated with denial (Table 3). However, the ratio of RDS Identification / RDS Denial was not significantly correlated with age for either group (ACC *r* = 0.12, HC *r* = 0.09), indicating that age-related changes don’t occur as a direct transition from least to most mature defenses. However, 2 × 2 ANOVA of defense type (denial/identification) by subject group conducted separately with 3 age groups revealed a clear developmental progression. The interaction effect supporting the first hypothesis was seen in adults 18 and older (AgCC *N* = 12, HC *N* = 15; Figure 1B), with the same pattern of results in post-hoc comparisons (participants with AgCC were more likely than healthy participants to use denial, *U* = 53, *p* = 0.038, and were less likely to use identification, *U* = 44, *p* = 0.013). The interaction effect was not evident in the under-age-12 group (AgCC *N* = 8, HC *N* = 11), despite a large overall difference in defense type (denial > identification), *F*(1,17) = 47.61, *p* < 0.001, $\eta_{\text{p}}^{2}$ = 0.737. No contrasts were significant in the 12–17 year old group (AgCC *N* = 7, HC *N* = 4), which was a notably smaller sample.

Finally we examined associations with cognitive abilities. Among participants with AgCC denial was negatively correlated with VIQ and identification was positively correlated with word count per story (Table 3). There were no other significant findings.

## Discussion

The present study sought to answer the following question: Is interhemispheric integration critical for normal development of defense mechanisms? We answered this question positively examining the stories generated in response to Thematic Apperception Test (TAT) figures by a unique sample of 27 otherwise high-functioning individuals who were born without the corpus callosum (AgCC), the largest structure connecting the two cerebral hemispheres. Using a well validated method to score defenses from the TAT (Defense Mechanism Manual, DMM; Cramer, 1991a, 2007; Porcerelli et al., 1998), participants with AgCC were found to use significantly more denial and significantly less identification relative to healthy volunteers matched for sex, age, and intelligence.

The use of more denial and less identification among AgCC participants is consistent with a pattern of defense usage expected in younger developmental ages (Cramer, 2007) and supports the conclusion that inter-hemispheric information transfer during development is critical for age-appropriate maturation of defenses. Furthermore, the present study confirmed the orderly progression of defense development as a function of (brain) maturation, as denial was relatively more frequent in earlier and identification in later developmental stages among healthy participants. Absence of the corpus callosum from birth may be further inferred to disrupt the orderly progression of defenses because the only correlation found to be significant among participants with AgCC was between older age and identification. Because the difference in defense usage between healthy individuals and individuals with AgCC was particularly apparent after later adolescence (age 18 and older), the difference in defense usage between AgCC and healthy participants may be posited to require a full trajectory of maturation among healthy individuals to emerge. Due to the small sample of adolescents in this study, we cannot provide greater detail regarding the age at which the pattern of defense mechanisms in individuals with AgCC diverges from their peers. Nonetheless, our findings indicate that the halt in defenses maturation among individuals with AgCC can only be evident when compared with healthy individuals who are well on their way to effective maturation completion.

Among individuals with AgCC, denial was lower among participants with greater verbal intelligence whereas identification was generally more employed by individuals who were capable of producing more words per story. In the healthy comparison group, defense mechanisms were relatively independent from all cognitive measures. These findings suggest that greater verbal intelligence and greater capacity for verbal fluency in producing stories may be able to compensate for otherwise more immature defense responses in AgCC.

Before discussing the findings of the present study some limitations must be acknowledged. Individuals with AgCC may show other anatomical changes in addition to the agenesis of the corpus callosum. These include intrahemispheric white-matter abnormalities including reduction of ipsilateral cortical association tracts or misrouted callosal fibers running parallel to the interhemispheric fissure (Tovar-Moll et al., 2007), abnormal microstructure and reduced volume of the ventral cingulum bundle (Nakata et al., 2009) or a number reduction of von Economo neurons, large spindle-shaped neurons localized to anterior cingulated cortex, and frontoinsular cortex (Kaufman et al., 2008) that have been posited to have a role in social cognition. This limits our ability to attribute poor development of defense mechanisms exclusively to absence of the corpus callosum. In addition, complete callosal absence in AgCC does not result in complete interruption of inter-hemispheric information transfer. Notably, while inter-hemispheric connectivity in AgCC may be within the normal range when quantified from resting-state fMRI, task-based transfer of information is nonetheless reduced (Paul et al., 2007; Tyszka et al., 2011). Separate analyses of complete and partial AgCC participants did not yield significant differences in defenses, indicating that even incomplete callosal disconnection may impact maturation of defense mechanisms. A further question concerns the stability of defense mechanisms, given that the measurements in the present study were limited to one session. However, outside of childhood and adolescent development, findings generally suggest that defensive functioning demonstrates relative stability over time (Perry, 2001; Perry and Bond, 2012).

Defense mechanisms develop out of interactions between an individual and parental figures or other significant caregivers. We do not have information on the quality of relationships between AgCC participants and their significant caregivers during development. These interactions may have carried some effect on defense development as poor social interaction (Paul et al., 2014) may be one of the mechanisms by which primary AgCC contributes to erratic development of defenses.

Some (*N* = 2) AgCC participants took anti-seizure medications (valproic acid, or phenytoin), but these participants’ defense scores did not fall outside the AgCC group distribution. Potential participants were excluded if they had a diagnosis of schizophrenia or other psychotic disorder, or diagnosis of bipolar disorder, but we did not test for other psychiatric symptoms or behavioral difficulties that may occur among individuals with primary AgCC. The association between psychiatric and other developmental symptomatology and defense mechanisms in AgCC may be a fruitful area of exploration in future studies. However, we did not have any hypotheses regarding such an exploration, and given our small sample size, we consider it beyond the scope of the present study.

Atypical defense maturation in agenesis of the corpus callosum offers evidence that defenses require large scale neuronal integration. The primary integration at issue here would be the cross-talk between the two cerebral hemispheres, or a subset of cortical homologues. It is also possible that there are contributions from developmental abnormalities in brain regions subserving cognitive control mechanisms, emotion processing and self functions. For example, the medial prefrontal/anterior cingulate cortex, a brain region which is structurally abnormal in AgCC has been highlighted in neuroimaging studies of emotion regulation (Dixon et al., 2017), cognitive control (Kerns et al., 2004), and cognitive dissonance (Botvinick et al., 2004). In AgCC, the cortex along the medial wall of each hemisphere typically features a radial organization, with no structural equivalent of anterior cingulate cortex (Hetts et al., 2006).

Several other brain regions implicated in processing defenses may also be involved. For example, as briefly reviewed above, denial of deficit as a consequence of right hemisphere damage (Solms, 2000) has been understood as a defense. The direct implication of focal brain damage in the occurrence of new defenses remains speculative, for lesion studies lack assessment of defense styles prior to brain damage. Confabulation is an example of a behavior that is addressed by both psychodynamic theorists and clinical neurology. Similar to the example of denial of deficit described above, confabulation has also been interpreted as a defense mechanism, even when it occurs following brain – frontal lobe – damage (Turnbull and Solms, 2007). In the neurological literature, confabulation has also been described in individuals who underwent surgical callosotomy as treatment of intractable epilepsy. Based on observation of confabulation patterns in these individuals, Michael Gazzaniga proposed that the left hemisphere serves as an *interpreter* of incomplete and ambiguous information, including information related to one’s own behavior and identity (Gazzaniga, 2000). When the corpus is absent from birth, it is possible that the interpreter will not be receiving adequate information from throughout the brain. Reliance on inaccurate or incomplete information may produce a net effect not distinguishable from denial. It should nonetheless be kept in mind that denial in standard psychoanalytic thinking is a function of the mind forcefully keeping away from awareness information that is posited to be particularly caustic for the self. The above neurological model(s) fail to account for the specific type of the signals kept away from awareness, or, in other words, why some and not others based on the quality (what) and not on the accuracy (how much) of information as often observed in standard psychoanalytic practice.

A study of brain activation during suppression (i.e., willfully placing disturbing thoughts out of awareness) found elevated activity in the bilateral dorsal and ventral lateral frontal lobe, the anterior cingulate gyrus, pre-motor regions, the intraparietal sulcus (BA 7) and right putamen, and found depressed activation in the hippocampus (Anderson et al., 2004). Whereas in standard psychoanalytic theory defenses may occur outside the realm of awareness, with these limitations in mind these results suggest that a coordinated set of brain regions is at work during (aware) defense generation and consequently this process may be disrupted by malfunctioning in any of these specific regions or the pathways which connect them.

Intelligence and defense mechanisms are both resources for psychosocial adaptation (Cramer, 2006). However, verbal and performance IQ are generally unrelated to defenses in children and adolescents (Cramer and Brilliant, 2001) and only moderately related in young and middle adulthood (Cramer, 2003). In the AgCC group, VIQ was negatively correlated with denial scores and word count was positively correlated with identification scores. A parsimonious explanation of this phenomenon is that in AgCC, a neurological condition where social skills tend to be less efficient, individuals may depend more heavily on verbal (symbolic) intelligence to support and compensate personal and social psychological adaptation. The findings in this study suggest that in AgCC verbal skills offer compensatory strategies, such that dependence on verbal intelligence is particularly relevant for modulating use of defenses at the lower developmental level and verbal generativity is relevant for modulating use of higher-level defenses.

Atypical behavior in adolescents and adults with isolated AgCC and normal-range intelligence scores is most likely to emerge when engaging in complex novel problem solving (Gott and Saul, 1978; Sauerwein et al., 1994; Brown et al., 2005a) and processing of socially relevant material (Paul et al., 2004; Symington et al., 2004; Turk et al., 2010). On tasks involving more complex cognitive and social processes, their performance is particularly limited by slow reaction times and processing speed (Marco et al., 2012), poor comprehension of syntax and linguistic pragmatics (Sanders, 1989; Banich and Brown, 2000), restricted verbal expression of emotional experience (Paul et al., 2006), poor comprehension of humor and literal interpretation bias (Paul et al., 2003; Brown et al., 2005a, b; Huber-Okrainec et al., 2005), and difficulty imagining other’s social perspective (Paul et al., 2004; Symington et al., 2004; Turk et al., 2010). Larger longitudinal studies examining cognitive, social, and psychological development in parallel are needed to clarify the relationship between these domains.

## Conclusion

In conclusion, the present research has shown that the smooth maturation of defense mechanisms (denial, projection, and identification) is affected in absence of the corpus callosum. Specifically individuals with AgCC show greater use of denial and lesser use of identification relative to healthy volunteers. These findings are consistent with the general view that large scale interaction among brain regions is needed to support utilization of age-appropriate defense mechanisms.

## Data Availability Statement

All datasets generated for this study are included in the article/Supplementary Material.

## Ethics Statement

The studies involving human participants were reviewed and approved by the Human Subjects Review Committee at the Travis Research Institute (data collection) and Institutional Review Board of the California Institute of Technology (data analysis). Written informed consent to participate in this study was provided by participants ages 18 and older and by the participants’ legal guardian/next of kin of participants ages 17 and below.

## Author Contributions

LP and SP designed the study. LP and WB collected the data. SP and JP administered the DMM scoring. LP analyzed the data. SP, LP, RA, and DT drafted the manuscript. JP edited the manuscript. All the authors commented on the manuscript.

## Conflict of Interest

The authors declare that the research was conducted in the absence of any commercial or financial relationships that could be construed as a potential conflict of interest.

## Supplementary Material

DATA S1 TAT stories from individuals with AgCC.

DATA S2 TAT stories from comparison group.

## References

[B1] Albucher R. C. Abelson J. L. Nesse R. M. (1998). Defense mechanism changes in successfully treated patients with obsessive-compulsive disorder. *Am. J. Psychiatry* 155 558–559. 10.1176/ajp.155.4.558 9546006

[B2] American Psychiatric Association (2013). *Diagnostic and Statistical Manual of Mental Disorders, DSM-5*, 5th Edn Washington, DC: American Psychiatric Association.

[B3] Anderson M. C. Ochsner K. N. Kuhl B. Cooper J. Robertson E. Gabrieli S. W. (2004). Neural systems underlying the suppression of unwanted memories. *Science* 303 232–235. 10.1126/science.1089504 14716015

[B4] Andrews G. Singh M. Bond M. (1993). The defense style questionnaire. *J. Nerv. Ment. Dis.* 181 246–256. 8473876 10.1097/00005053-199304000-00006

[B5] Ballmaier M. Kumar A. Elderkin-Thompson V. Narr K. L. Luders E. Thompson P. M. (2008). Mapping callosal morphology in early- and late-onset elderly depression: an index of distinct changes in cortical connectivity. *Neuropsychopharmacology* 33 1528–1536. 10.1038/sj.npp.1301538 17712348 PMC2810852

[B6] Banich M. Brown W. S. (2000). A life-span perspective on interaction between the cerebral hemispheres. *Dev. Neuropsychol.* 18 1–10. 10.1207/S15326942DN1801_1 11143800

[B7] Bedeschi M. F. Bonaglia M. C. Grasso R. Pellegri A. Garghentino R. R. Battaglia M. A. (2006). Agenesis of the corpus callosum: clinical and genetic study in 63 young patients. *Pediatr. Neurol.* 34 186–193. 10.1016/j.pediatrneurol.2005.08.008 16504787

[B8] Besharat M. A. Khajavi Z. (2013). The relationship between attachment styles and alexithymia: mediating role of defense mechanisms. *Asian J. Psychiatr.* 6 571–576. 10.1016/j.ajp.2013.09.003 24309875

[B9] Bibring G. L. Dwyer T. F. Huntington D. S. Valenstein A. F. (1961). Contributions to psychoanalytic theory. A study of the psychological processes in pregnancy and of the earliest mother-child relationship. *Psychoanal. Study Child* 16 9–72.

[B10] Bogen J. Frederiks J. (eds) (1985). “Split-brain syndromes,” in *Handbook of Clinical Neurology*, Vol. 45 (Amsterdam: Elsevier Science Publishing Co), 99–106.

[B11] Botvinick M. M. Cohen J. D. Carter C. S. (2004). Conflict monitoring and anterior cingulate cortex: an update. *Trends Cogn. Sci.* 8 539–546. 10.1016/j.tics.2004.10.003 15556023

[B12] Brown W. S. Paul L. K. (2019). The neuropsychological syndrome of agenesis of the corpus callosum. *J. Int. Neuropsychol. Soc.* 25 324–330. 10.1017/S135561771800111X 30691545 PMC7989584

[B13] Brown W. S. Paul L. K. Symington M. Dietrich R. (2005a). Comprehension of humor in primary agenesis of the corpus callosum. *Neuropsychologia* 43 906–916. 10.1016/j.neuropsychologia.2004.09.008 15716161

[B14] Brown W. S. Symington M. VanLancker-Sidtis D. Dietrich R. Paul L. K. (2005b). Paralinguistic processing in children with callosal agenesis: emergence of neurolinguistic deficits. *Brain Lang.* 93 135–139. 10.1016/j.bandl.2004.09.003 15781301

[B15] Buchanan D. C. Waterhouse G. J. West S. C. Jr. (1980). A proposed neurophysiological basis of alexithymia. *Psychother. Psychosom.* 34 248–255. 10.1159/000287465 7280165

[B16] Chiappedi M. Bejor M. (2010). Corpus callosum agenesis and rehabilitative treatment. *Ital. J. Pediatr.* 36:64. 10.1186/1824-7288-36-64 20849621 PMC2949675

[B17] Chiappedi M. Fresca A. Baschenis I. M. (2012). Complete corpus callosum agenesis: can it be mild? *Case Rep. Pediatr.* 2012:752751. 10.1155/2012/752751 22973527 PMC3437615

[B18] Cramer P. (1987). The development of defense mechanisms. *J. Pers.* 55 597–614. 10.1111/j

[B19] Cramer P. (1991a). Anger and the use of defense-mechanisms in college-students. *J. Pers.* 59 39–55. 10.1111/J.1467-6494.1991.Tb00767.X 2037963

[B20] Cramer P. (1991b). *The Development of Defense Mechanisms. Theory, Research and Assessment.* New York, NY: Springer-Verlag.

[B21] Cramer P. (1997). Evidence for change in children’s use of defense mechanisms. *J. Pers.* 65 233–247. 10.1111/J.1467-6494.1997.Tb00954.X 9226941

[B22] Cramer P. (2003). Personality change in later adulthood is predicted by defense mechanism use in early adulthood. *J. Res. Pers.* 37 76–104. 10.1016/s0092-6566(02)00528-7

[B23] Cramer P. (2006). *Protecting the Self: Defense Mechanisms in Action.* New York, NY: The Guilford Press.

[B24] Cramer P. (2007). Longitudinal study of defense mechanisms: late childhood to late adolescence. *J. Pers.* 75 1–24. 10.1111/j.1467-6494.2006.00430.x 17214589

[B25] Cramer P. Brilliant M. A. (2001). Defense use and defense understanding in children. *J. Pers.* 69 297–322. 10.1111/1467-6494.00147 11339801

[B26] Dixon M. L. Thiruchselvam R. Todd R. Christoff K. (2017). Emotion and the prefrontal cortex: an integrative review. *Psychol. Bull.* 143 1033–1081. 10.1037/bul0000096 28616997

[B27] Fenichel O. (1946). *The Psychoanalytic theory of Neurosis.* London: W. W. Norton & Co.

[B28] Freud A. (1936). *The Ego and the Mechanisms of Defense.* London: Hogarth Press.

[B29] Freud S. Breuer J. (1895). *Studies on Hysteria.* London: Hogarth Press.

[B30] Gazzaniga M. S. (2000). Cerebral specialization and interhemispheric communication: does the corpus callosum enable the human condition? *Brain* 123 (Pt 7), 1293–1326. 10.1093/brain/123.7.1293 10869045

[B31] Glass G. V. Peckham P. D. Sanders J. R. (1972). Consequences of failure to meet assumptions underlying the fixed effects analyses of variance and covariance. *Rev. Educ. Res.* 42 237–288. 10.3102/00346543042003237

[B32] Glass H. C. Shaw G. M. Ma C. Sherr E. H. (2008). Agenesis of the corpus callosum in California 1983–2003: a population-based study. *Am. J. Med. Genet. A* 146A 2495–2500. 10.1002/ajmg.a.32418 18642362 PMC2574703

[B33] Gott P. S. Saul R. E. (1978). Agenesis of the corpus callosum: limits of functional compensation. *Neurology* 28 1272–1279. 10.1212/wnl.28.12.1272 569786

[B34] Handbook of Psychiatric Measures (2008). *Handbook of Psychiatric Measures*, 2nd Edn Washington, DC: American Psychiatric Publishing, Inc.

[B35] Harwell M. R. Rubinstein E. N. Hayes W. S. Olds C. C. (1992). Summarizing Monte Carlo results in methodological research: the one- and two-factor fixed effects ANOVA cases. *J. Educ. Stat.* 17 315–339. 10.3102/10769986017004315

[B36] Helmes E. McNeill P. D. Holden R. R. Jackson C. (2008). The construct of alexithymia: associations with defense mechanisms. *J. Clin. Psychol.* 64 318–331. 10.1002/jclp.20461 18302210

[B37] Hetts S. W. Sherr E. H. Chao S. Gobuty S. Barkovich J. (2006). Anomalies of the corpus callosum: an MR analysis of the phenotypic spectrum of associated malformations. *AJR Am. J. Roentgenol.* 187 1343–1348. 10.2214/AJR.05.0146 17056927

[B38] Hoppe K. D. (1977). Split brains and psychoanalysis. *Psychoanal. Q.* 46 220–244. 10.1080/21674086.1977.11926798 870923

[B39] Huber-Okrainec J. Blaser S. E. Dennis M. (2005). Idiom comprehension deficits in relation to corpus callosum agenesis and hypoplasia in children with spina bifida meningomyelocele. *Brain Lang.* 93 349–368. 10.1016/j.bandl.2004.11.002 15862859

[B40] Kaplan-Solms K. Solms M. (2000). *Clinical Studies in Neuro-Psychoanalysis: Introduction to a Depth Neuropsychology.* New York, NY: Karnac Books.

[B41] Kaufman J. A. Paul L. K. Manaye K. F. Granstedt A. E. Hof P. R. Hakeem A. Y. (2008). Selective reduction of Von Economo neuron number in agenesis of the corpus callosum. *Acta Neuropathol.* 116 479–489. 10.1007/s00401-008-0434-7 18815797

[B42] Keiser R. E. Prather E. N. (1990). What is the TAT? A review of ten years of research. *J. Pers. Assess.* 55 800–803. 10.1080/00223891.1990.9674114 2280342

[B43] Kerns J. G. Cohen J. D. MacDonald A. W. III Cho R. Y. Stenger V. A. Carter C. S. (2004). Anterior cingulate conflict monitoring and adjustments in control. *Science* 13 1023–1026. 10.1126/science.1089910 14963333

[B44] Knyazeva M. G. (2013). Splenium of corpus callosum: patterns of interhemispheric interaction in children and adults. *Neural Plast.* 2013:639430. 10.1155/2013/639430 23577273 PMC3610378

[B45] Kohut H. (1977). *The Restoration of the Self.* Madison, CT: International Universities Press.

[B46] Lampl-de Groot J. (1985). *Man and Mind: Collected Papers of Jeanne Lampl-de Groot, M.D.* New York, NY: International Universities Press.

[B47] Lebel C. Walker L. Leemans A. Phillips L. J. Beaulieu C. (2008). Microstructural maturation of the human brain from childhood to adulthood. *Neuroimage* 40 1044–1055. 10.1016/j.neuroimage.2007.12.053 18295509

[B48] Lix L. M. Keselman J. C. Keselman H. J. (1996). Consequences of assumption violations revisited: a quantitative review of alternatives to the one-way analysis of variance “F” test. *Rev. Educ. Res.* 66 579–619. 10.3102/00346543066004579

[B49] Lowenstein R. M. Kris E. Hartmann H. (1975). *Éléments de Psychologie Psychanalytique.* Paris: Presses Universitaires de France.

[B50] Luders E. Thompson P. M. Toga A. W. (2010). The development of the corpus callosum in the healthy human brain. *J. Neurosci.* 30 10985–10990. 10.1523/JNEUROSCI.5122-09.2010 20720105 PMC3197828

[B51] Marco E. J. Harrell K. M. Brown W. S. Hill S. S. Jeremy R. J. Kramer J. H. (2012). Processing speed delays contribute to executive function deficits in individuals with agenesis of the corpus callosum. *J. Int. Neuropsychol. Soc.* 18 521–529. 10.1017/S1355617712000045 22390821 PMC3605885

[B52] Meissner W. W. (1974). The role of imitative social learning in identificatory processes. *J. Am. Psychoanal. Assoc.* 22 512–536. 10.1177/000306517402200303 4455725

[B53] Morriss M. C. Zimmerman R. A. Bilaniuk L. T. (1999). Changes in brain water diffusion during childhood. *Neuroradiology* 41 929–934. 10.1007/s002340050869 10639670

[B54] Murray H. A. (1943). *Thematic Apperception Test Manual.* Cambridge, MA: Harvard University Press.

[B55] Nakata Y. Barkovich A. J. Wahl M. Strominger Z. Jeremy R. J. Wakahiro M. (2009). Diffusion abnormalities and reduced volume of the ventral cingulum bundle in agenesis of the corpus callosum: a 3T imaging study. *AJNR Am. J. Neuroradiol.* 30 1142–1148. 10.3174/ajnr.A1527 19246528 PMC3777656

[B56] Northoff G. Bermpohl F. Schoeneich F. Boeker H. (2007). How does our brain constitute defense mechanisms? First-person neuroscience and psychoanalysis. *Psychother. Psychosom.* 76 141–153. 10.1159/000099841 17426413

[B57] Northoff G. Boeker H. (2006). Principles of neuronal integration and defense mechanisms: neuropsychoanalytic hypothesis. *Neuropsychoanalysis* 8 69–84. 10.1080/15294145.2006.10773514

[B58] Oldfield R. C. (1971). The assessment and analysis of handedness: the Edinburgh inventory. *Neuropsychologia* 9 97–113. 10.1016/0028-3932(71)90067-4 5146491

[B59] Paul L. K. Brown W. S. Adolphs R. Tyszka J. M. Richards L. J. Mukherjee P. (2007). Agenesis of the corpus callosum: genetic, developmental and functional aspects of connectivity. *Nat. Rev. Neurosci.* 8 287–299. 10.1038/nrn2107 17375041

[B60] Paul L. K. Corsello C. Kennedy D. P. Adolphs R. (2014). Agenesis of the corpus callosum and autism: a comprehensive comparison. *Brain* 137 1813–1829. 10.1093/brain/awu070 24771497 PMC4072909

[B61] Paul L. K. Lautzenhiser A. Brown W. S. Hart A. Neumann D. Spezio M. (2006). Emotional arousal in agenesis of the corpus callosum. *Int. J. Psychophysiol.* 61 47–56. 10.1016/j.ijpsycho.2005.10.017 16759726

[B62] Paul L. K. Schieffer B. Brown W. S. (2004). Social processing deficits in agenesis of the corpus callosum: narratives from the Thematic Appreciation Test. *Arch. Clin. Neuropsychol.* 19 215–225. 10.1016/S0887-6177(03)00024-6 15010087

[B63] Paul L. K. Van Lancker-Sidtis D. Schieffer B. Dietrich R. Brown W. S. (2003). Communicative deficits in agenesis of the corpus callosum: nonliteral language and affective prosody. *Brain Lang.* 85 313–324. 10.1016/s0093-934x(03)00062-2 12735947

[B64] Perry J. C. (1990). *The Defense Mechanism Rating Scales*, 5th Edn Cambridge, MA: Perry.

[B65] Perry J. C. (2001). A pilot study of defenses in adults with personality disorders entering psychotherapy. *J. Nerv. Ment. Dis.* 189 651–660. 10.1097/00005053-200110000-00001 11708665

[B66] Perry J. C. Bond M. (2012). Change in defense mechanisms during long-term dynamic psychotherapy and five-year outcome. *Am. J. Psychiatry* 169 916–925. 10.1176/appi.ajp.2012.11091403 22885667

[B67] Porcerelli J. H. Sandler B. A. (1995). Narcissism and empathy in steroid users. *Am. J. Psychiatry* 152 1672–1674. 10.1176/ajp.152.11.1672 7485634

[B68] Porcerelli J. H. Thomas S. Hibbard S. Cogan R. (1998). Defense mechanisms development in children, adolescents, and late adolescents. *J. Pers. Assess.* 71 411–420. 10.1207/S15327752jpa7103_9 9933944

[B69] Sanders R. J. (1989). Sentence comprehension following agenesis of the corpus callosum. *Brain Lang.* 37 59–72. 10.1016/0093-934x(89)90101-6 2752275

[B70] Sauerwein H. C. Nolin P. Lassonde M. (1994). “Cognitive functioning in callosal agenesis,” in *Callosal Agenesis: A Natural Split Brain?*, eds Lassonde M. Jeeves M. A. (New York, NY: Plenum Press), 221–233. 10.1007/978-1-4613-0487-6_23

[B71] Schell-Apacik C. C. Wagner K. Bihler M. Ertl-Wagner B. Heinrich U. Klopocki E. (2008). Agenesis and dysgenesis of the corpus callosum: clinical, genetic and neuroimaging findings in a series of 41 patients. *Am. J. Med. Genet. A* 146A 2501–2511. 10.1002/ajmg.a.32476 18792984 PMC2774850

[B72] Solms M. (2000). Dreaming and REM sleep are controlled by different brain mechanisms. *Behav. Brain Sci.* 23 843–850; discussion 904–1121. 10.1017/s0140525x00003988 11515144

[B73] Sperry R. W. (1968). Hemisphere deconnection and unity in conscious awareness. *Am. Psychol.* 23 723–733. 10.1037/h0026839 5682831

[B74] Sperry R. W. Gazzaniga M. Bogen J. Vinken P. J. Bruyn G. W. (1969). Interhemispheric relationships: the neocortical commissures; syndromes of hemisphere disconnection. *Handb. Clin. Neurol.* 4 273–290.

[B75] Symington S. Paul L. Ono M. Symington M. Brown W. (2004). “Theory of mind in individuals with agenesis of the corpus callosum,” in *Proceedings of the International Neuropsychological Society*, New York, NY.

[B76] Tang P. H. Bartha A. I. Norton M. E. Barkovich A. J. Sherr E. H. Glenn O. A. (2009). Agenesis of the corpus callosum: an MR imaging analysis of associated abnormalities in the fetus. *Am. J. Neuroradiol.* 30 257–263. 10.3174/ajnr.A1331 18988682 PMC7051410

[B77] Taylor G. J. Bagby R. M. Parker J. D. A. (1997). *Disorders of Affect Regulation: Alexithymia in Medical and Psychiatric Illness.* Cambridge: Cambridge University Press.

[B78] TenHouten W. D. Hoppe K. D. Bogen J. E. Walter D. O. (1985a). Alexithymia and the split brain. I. Lexical-level content analysis. *Psychother. Psychosom.* 43 202–208. 10.1159/000287880 4034891

[B79] TenHouten W. D. Hoppe K. D. Bogen J. E. Walter D. O. (1985b). Alexithymia and the split brain. III. Global-level content analysis of fantasy and symbolization. *Psychother. Psychosom.* 44 89–94. 10.1159/000287898 2419941

[B80] Tomasch J. (1954). Size, distribution, and number of fibres in the human corpus callosum. *Anat. Rec.* 119 119–135. 10.1002/ar.1091190109 13181005

[B81] Tovar-Moll F. Moll J. de Oliveira-Souza R. Bramati I. Andreiuolo P. A. Lent R. (2007). Neuroplasticity in human callosal dysgenesis: a diffusion tensor imaging study. *Cereb. Cortex* 17 531–541. 10.1093/cercor/bhj178 16627861

[B82] Turk A. Brown W. S. Symington M. Paul L. K. (2010). Social narratives in agenesis of the corpus callosum: linguistic analysis of the Thematic Apperception Test. *Neuropsychologia* 48 43–50. 10.1016/j.neuropsychologia.2009.08.009 19686767

[B83] Turnbull O. H. Evans C. E. Owen V. (2005). Negative emotions and anosognosia. *Cortex* 41 67–75. 10.1016/s0010-9452(08)70179-5 15633708

[B84] Turnbull O. H. Fotopoulou A. Solms M. (2014). Anosognosia as motivated unawareness: the ‘defence’ hypothesis revisited. *Cortex* 61 18–29. 10.1016/j.cortex.2014.10.008 25481464

[B85] Turnbull O. H. Jones K. Reed-Screen J. (2002). Implicit awareness of deficit in anosognosia? An emotion-based account of denial of deficit. *Neuropsychoanalysis* 4 69–87. 10.1080/15294145.2002.10773381

[B86] Turnbull O. H. Solms M. (2007). Awareness, desire, and false beliefs: Freud in the light of modern neuropsychology. *Cortex* 43 1083–1090. 10.1016/s0010-9452(08)70706-8 18044668

[B87] Tyszka J. M. Kennedy D. P. Adolphs R. Paul L. K. (2011). Intact bilateral resting-state networks in the absence of the corpus callosum. *J. Neurosci.* 31 15154–15162. 10.1523/JNEUROSCI.1453-11.2011 22016549 PMC3221732

[B88] Vaillant G. E. (1994). Ego mechanisms of defense and personality psychopathology. *J. Abnorm. Psychol.* 103 44–50. 10.1037//0021-843x.103.1.44 8040479

[B89] Vaillant G. E. (2011). Involuntary coping mechanisms: a psychodynamic perspective. *Dialogues Clin. Neurosci.* 13 366–370. 22034454 10.31887/DCNS.2011.13.2/gvaillant PMC3182012

[B90] Wallerstein R. S. (1983). Defenses, defense mechanisms, and the structure of the mind. *Am. Psychoanal. Assoc.* 31S 201–225.

[B91] Westen D. Gabbard G. O. (2002a). Developments in cognitive neuroscience: I. Conflict, compromise, and connectionism. *J. Am. Psychoanal. Assoc.* 50 53–98. 10.1177/00030651020500011501 12018875

[B92] Westen D. Gabbard G. O. (2002b). Developments in cognitive neuroscience: II. Implications for theories of transference. *J. Am. Psychoanal. Assoc.* 50 99–134. 10.1177/00030651020500011601 12018876

