# Supplementary material for: Integration Between Cerebral Hemispheres Contributes to Defense Mechanisms
Source: Front Psychol. 2020 Jul 7;11:1534. doi: 10.3389/fpsyg.2020.01534 (PMC7359856; doi:10.3389/fpsyg.2020.01534)
Supplement: DATA S1 — TAT stories from individuals with AgCC. [file Data_Sheet_1.PDF]

NOTE: Examiner comments in green. Q=general query. Th = what were they thinking? F = What were they feeling?

1

There was a boy thinking about playing his violin, but he did not know how. So he decided to sit and think about how he would play the violin one day and how great he would become. (**Q**) A little depressed. (**Q**) He got a brand new violin.

Back in 1800s, Mary, her husband John and Mary's mother. They were busy building a new plantation in which they would live. Feeling depressed and tired because they have to work so hard. Long day is taking a toll on them. It is going to turn out good because it is what they always wanted, to farm. I do not know what she is doing.

Looks like they are feeling really depressed because heard some bad news, that somebody died. Are wondering how this could happen, but it is all going to turn out fine in the end but somebody died close to them.

A serious moment. Doctor and assistant. Doctors operating on a guy who got wounded in war and they are just taking out a bullet. They will take it out, patch him up and he'll be ready to go. (The boy?) no idea.

The boy is sick. He's not feeling too good right now. Father came to check on him. Father cares about him a lot and hopes he'll be ok. [How does it end up?] Cold winter, he's going to die.

The guy, the father just walked in on his daughter, can't believe he saw her naked, can't wait to get out, is embarrassed. He really wants to leave, hopes she'll never find out he saw her naked.

A young boy is at his violin. He just received it yesterday for his school orchestra. He is concerned because he does not know how to play his violin. In the end he goes to his teacher to ask for help. The teacher begins to give him lessons and soon he is playing in the school orchestra, happier than before.

One day on a large farm there was a young girl who wants more adventure in her life. She does not have the money to move away so she decides to save money and go to school after which she will graduate and go to college. The mother is sad, she does not want her daughter leaving. Her father works hard in the fields so that he may not think about his daughter leaving him. She agrees with the family to send money to help pay for groceries and miscellaneous items. The family then agrees that this move for her is okay. They send her off with a joyful goodbye instead of a sad one.

The young son stands quietly while his distressed mother looks at the wall. They have just lost a very dear member of the family and can say nothing about it. The son makes efforts to comfort his mother but it is no use for she is too sad. The son decides to live with his mother for another two months and care for her. **(Q)** Both are sort of speechless.

A young boy stares out as his dear friend is having surgery from a bullet wound fighting in the war. He feels a certain responsibility of being loyal to his friend. He waits there impatiently as the doctors cut and perform surgery on his friend. The doctors are very hopeful for a successful surgery but there is a slight chance he might not make it. The boy sits in the room during the surgery watching and waiting impatiently for his friend to rise up from the surgical table and give him a giant hug. His friend gets up off the surgical table and two hours of surgery. The doctors tell the young boy that he is going to be okay.

An old man stands over his grandson feeling a bit sad yet hopeful. He refuses to take his grandson to the hospital for proper care. In the old days some people believe that ritual

magic could revive an unconscious soul. The old man combines all his strength to revive the young boy on the bed. As he waves his hand up and down over the young boy's body, the man is performing a chant. After about an hour of ritual the young boy opens his eyes and a tear of joy drops from the cheek of the old man. The old man goes on before he passes to teach the young boy about the old ways of medicine.

One night in a bar, a young blond haired man walks in. He looks around for someone to take home that night for a passionate rendezvous. He then after an hour spies a beautiful young brunette woman sitting at the bar having a drink. The man approaches her and whispers in her ear. The woman after hearing such gentle words could not help herself but to find out what this man had to offer. He then takes her home with him and makes passionate love to her which seems to go on forever. Lying in bed, he asks her what her name is. After realizing who the woman was the man has sort of a very shocked expression on his face. He feels very surprised at the identity of this woman. The woman turns out to be his long lost first cousin. He very quickly jumps out of bed as the woman falls asleep from exhaustion. He quickly puts on his clothes and looks down using his arm to shield his eyes. He feels terrible, he realizes that the world can be a very small place even for a man with lust. He calls her later and as they talk he apologizes for the previous night's event. She agrees to stay friends with this man and he moves on to himself. He thinks "Oh well, another bar, another night, another woman, another adventure."

Looks to me like this little boy is frustrated because he is trying to figure out how to play this instrument. It is a violin. He could either learn how to play, or because of his age he looks like he would rather go outside to ride his bicycle or play or something. [What happened before?] He could have been playing or watching Television and his parents came in and gave him this violin, and that is when he got, maybe he picked it up before and tried to play, or tried to figure out what it was. (Th?) He is thinking am I ever going to learn how to play this Either that or what am I going to do now.

OK. Looks like this girl here has probably told a relative, could be her mother that she is going to school. Looks like her mother is not too pleased cause it is more like she would rather have her on this farm, looks like a little farm, and she would rather have her stay there than go off to college or wherever she is going off to. [How does it turn out?] What happens next? Looks like despite her mother's wishes she is going to go off and go to school. She would rather pursue a dream that she has rather than stay on the farm. (F?) Sadness. Looks more like sad. Having to tell her mother that she would rather follow a dream than stay here. Her mom looks just as sad to see her go. . (Th?) Well the daughter looks more like she is thinking she is sad to leave and the mom is basically sad to see her daughter go.

This gentleman could be this lady's son. And she may have just told him some kind of sad news perhaps Maybe there was somebody in the family passed away or something. I say that because I see in her hand over here, something like a handkerchief. And he looks like he cannot believe that [What happens next?] Well. They could possibly embrace. Maybe reminisce about this relative of theirs. Wonder what happened. (F?) Looks more mostly like disbelief, cannot believe that he has gone, that they are gone. (Th?) Oh. They just heard the news, or like he did anyway, so he is still probably in a state of shock or something. Still cannot believe it.

This little boy it looks like he is thinking or he is kind of daydreaming, maybe about being a doctor. Of course with this gun, or this rifle, right here, it could be like of course he is not really wearing anything he is just in a coat and tie, I was going to say maybe he was in cadet school or something. Maybe one of his relatives was a doctor before him. [What happened before?] Before this? Maybe he was at target practice. I only say that because of the rifle in the picture here, but he does not have his hands on it so I cannot completely tell. Not for certain. (F?) Oh what he is thinking – he is thinking more like that way he will be in the future. [How does it turn out?] Um. It could turn out that he will pursue it for the immediate future he will probably put this rifle away and go do something else. Depending on where he is at . If it is military school they will have him doing something else. But he just puts his rifle away for now.

Looks like this gentleman who is leaning down is trying to wake this person up. The reason that I say this is because I notice that he is trying to wake this guy up, and he is not going to read the last rites or anything because the guy in the picture who is lying down, I notice that his knee is up most of the time when I have seen it anyway. It is just because this gentleman has his knee up. . [What happened before?] He just went in to take a nap and he told this guy to wake him up specifically at a certain time. [How does it turn out?] He will wake him up and this gentleman well I guess he will get ready to go where ever he had to go. (Th?) Well right now the one who is standing is thinking well I got to get him up. And the one who is lying down might be in a dream state or something. (F?) The one lying down he is just feeling peace because he is relaxed and sleeping and the other one is just trying to get him up, maybe worried that he will be late to where ever he needs to go. Just wants to make sure he gets up.

Well this gentleman here looks like he is still trying to wake himself up. He is dressed but last stretch and yawn. Looks like he might be ready to go to work. The lady in bed there might be his wife. She might not have to get up as early as he did. He figures he is up and he is getting ready to leave and he can just leave her there to sleep. . [What happened before?] Um. Oh they both went to bed, it might have been evening, and then he got up early and took a shower and then ate something, well he got up early and then took a

shower and then he will grab something to eat, maybe on his way out. . (Th?) Well she is asleep so she is probably not thinking too much just how much more time she has to sleep. Well he might be thinking about his day ahead. [How does it turn out?] He will leave eventually and she will get up and then she will start her day. She might do the same thing. Take a shower , get dressed. (F?)   Um. Oh he might just be feeling just a little tired from getting up early. And she is just feeling peace that she was asleep.

The little boy is in school reading a project that he is supposed to be doing, he is concentrating hard because he wants to be sure to get it all correct. He is confused liked me on what to say or do, but if he thinks hard enough he will be able to find a solution. At the end of his reading he finishes his project and gets an A. That is a book isn't it, it is not a musical instrument? Oh how dumb if it is. He is trying to figure out how to play it, he is reading the music. They need color pictures so you can tell what's there (F?) I don't want to do this, I don't like this instrument, and maybe if I sit here long enough it will play itself.

The beginning of the day the mother is standing by the tree looking at the sunrise while the daughter is headed off to school. The daughter is looking as her mother stares at the sun while the young man with the horse sits there handsomely. Just before he gets started on his day to work in the fields. I can't think of anything else [How does it end?] When the young man finished plowing the mother goes inside to cook dinner and the daughter comes home from school and another day is finished. (F?) daughter is looking at her mother thinking oh my mother is going to have another child and having to go through all of this again, but she looks very content, and the mother says I am going through all of this again with another child, but in the end it is all worth it. The young man is thinking okay another hard day at work, but it is a beautiful day.

Looks like Betsy Ross, the mother stands at the window waiting for her daughter to go out, the young man is also waiting. As the daughter gets ready he is sitting the mother that he will treat her daughter good, and that he loves her, and would like to ask for her daughters hand in marriage, but would like her blessing first. The mother is thinking about whether he would be good for her daughter, and in the end she decides yes she will.

Ooh, The young man is stranding there thinking of the days his father was at war, and how he got sick and had to operate on him. During those days they did things without anesthesia or medication or any sorts. so the young man thinks that in my day things are better and if he would have been alive today if he lived in this age. I will be a better father he thinks. It won't be like in my father's day. (F?) that if he studies hard he will be

able to save lives instead of losing lives.

The young man is very sick and the priest is standing over him praying for him to get better. At this last hour he is giving him his last rights, as the young man peacefully dies. (F?) sadness for such a young life to pass away, for he was so young, but he knows that it was his time to go.

the husband stands over his wife crying because his wife is dead. and wondering what is going to be of him now that she is gone. Wondering who did this or how this happened. He is totally devastated. The woman is dead so we can't say much about her. [How does it end?] he goes and tells his children that their mother is no longer with them and now they have to go on without her.

One day there was a little boy and he was looking at his violin and he was thinking about how he was going to make music come out of it. So he thought and he thought and he came to the conclusion that he would just start strumming and he figured that that would be how he would learn how to play the violin. [What happened before?] He was told he had to play the violin and he was frustrated so he thought and he thought about what he was going to do and that goes to the beginning of what I said. [How does it turn out?] He picked it up and he started strumming and he was happy with himself and music came out and that's how he learned how to play the violin.

Hm. Ok. One day a long time ago, there was a girl going off to school. She had been on this farm for a long time. One day she decided I am going to go and leave the farm and go to school because every day she did the same thing over and over again so the day she wanted a change was the day she looked over at her sister who was pregnant and living the same life over and over again. That's what made her decide to pick up her things and go to school and make a new life for herself. She knew that she would come back and she would still know the people but that she would be a better person. [How does it turn out?] she leaves and goes and she comes back and she's a better person and nobody holds anything against her for not staying on the farm and working and they love her still. (F?) Apprehension (Th?) she is thinking she has to do this to better herself.

This is a sad day. This is a mother and a son. The mother's husband, the son's father, died and the mother feels lost because they had been married for a long, long time. The son is sad but he knows he has to be strong for his mother so they are going to prepare for the funeral. It is a hard thing for them and the mother is very sad but they are going to do it and they will get all the preparations together and it will end up just fine and life will go on. The lady is thinking that she will be fine but she will have lost something in her heart.

These are such sad pictures! I can't even make them happy!

This is going to be bizarre. One day a little boy had to have an operation. He got all prepped up for the operation and he is on the operation table. The doctors are all around him and they are going to start operating on him. But something terribly goes wrong on the operating table and the boy sees the light from heaven and he sees himself all dressed up in a suit and he knows that he is going to die because you see there is a gun over here and he got shot from the gun. So he is at peace with himself as he sees the light to go to heaven. The doctors feel very sad that they couldn't save the little – well, young man. [How does it turn out?] the young man dies – he dies and goes to heaven. (Th?) the doctors are thinking how terrible – such a sad thing, such a terrible incident. The young man is not thinking anything.

Do you have any happy pictures?
This is a story about a lady who is very sick. She has been sick for a very long time. The family has decided to bring a healer in because nothing else has worked. The healer comes to the house and starts his healing process by praying over her and chanting and talking and spending time with her. In his mind he is doing everything he can to help her get better. [How does it turn out?] she dies and the healer feels bad. (Th?) the lady is not thinking anything, she is gone. The healer is thinking I can only do my best. (F?) the lady is not feeling anything. The healer is feeling hope.

Ok. How come her boobs are showing they didn't cover her boobs. OK this is going to be a terrible story this is a story about a man and a prostitute. He has come to visit the prostitute. They have had their time together and she is asleep and now it is time for him to leave. He gets up and gets dressed and is going to leave but he is very very ashamed of himself and after he feels all that, he leaves and she continues to sleep until she wakes up for the day. (Th?) The lady is not thinking nothing, anything. The man is feeling ashamed and glad that it is over that he finally that he finally knows what a prostitute does he was always curious and now it is over.

12

The story of a young boy who wanted to master the art of music and it was very difficult for him. At times he gave up and was frustrated by the whole thing but he was always told to never give up and the young boy did not understand why it was important to finish what you start and then once he finally mastered the violin he understood the meaning of that. (**Q**) He is disappointed and frustrated.

Seems to me that the young lady is determined to break away from the farm life. Maybe she is doing her schooling and that is her way out. She does not look happy with the situation there. And then she leaves, becomes rich, and in New York she finds her dreams. It is always like you have seen that in the movies before. (**Q**) Looks kind of like despaired, depressed, feeling lonely.

I see a son and his mother; looks like there was perhaps some kind of misunderstanding between them and I guess grandmother. His mother turned her back and he did not get his message through. (**Q**) looks disappointed and his mother has got that stern look like we have been over this before. (**Q**) He eventually leaves to deal with the generation gap and loves them anyway and they continue to have a good relationship.

This one sucks, they are cutting some guy open because here, There was some kind of accident and I take it that they are trying to rescue the guy. He was wounded and it looks like it's an urgent As if they are trying to get a bullet from the guy. This kid's face does not fit the bullet. There is some kind of detachment, the kid is not involved with this, it is not something important to him lying on the table. And I think maybe one of the old guys told him to turn his back because there might be bloody situation and he has a look on his face like he could not care less, callous about the whole thing, like he could not care less. (**Q**) just wants to get it over with because he has something on his mind (**Q**) save the guy's life and the kid goes and talks to one of the old guys who might be his father gets ten dollars or something and goes to see a movie or something with his girlfriend. He wanted money.

The Young man who is just looking for rest, trying to get some shuteye and his grandfather is trying to wake him up with divine intervention as he waves his hand over his face. And he says it is time for church and the young man looks up at his grandfather and says “I could care less I just want to rest.” The old man says “are you sure you do not care because with an attitude like that God is sure to strike you down. Remember if you want any kind of blessing in your life you have to love the Lord.” And the young man said “I will take my chances.”

This is sad. Definitely sad. Husband and wife. It is suicide. The husband knew that his wife had a drinking problem, used drugs and did all he could to save her even turned to the Lord, the Bible is on the table there, and everything failed. He walks in the room to find his wife lifeless and he cannot bear to see the sight and he turns his back to her, covers his eyes in disbelief, he does not want to see the truth.(**Q**) He becomes more of a religious fanatic and he goes to church every night of the week to forget his problems and that becomes his crutch.

He is thinking how to play a violin, very hard because he is not good at it. Frustrated that he cannot learn how to play it too well, he just leaves it alone for a while and thinks how he can learn how to play the violin. He has a frown, not touching it, just looking.

She is looking over the field and thinking if she does not study and go to school, she is going to have to work and be a farmer. When she goes to school and studies hard, she will find a better paying job and she will not suffer financially like the other farmers and she decides to go to school.

This man has problem with his mother, arguing about he wants to move out. She is sad and lonely because her son is going to leave her. He is frustrated because he feels like his mother is treating him like a baby and he wants to move on with his life and be more independent of his mother.

This is when this boy was involved in a violent act. Reminiscing about how he saw two bad guys cut open his friend. Feels very depressed and is never going to forget about it. Is going to try to find these two men who cut open his friend and do the same thing to them.

A very sick person and this is a priest who is going to heal the sick person, not medically but spiritually. Praying over her so that she gets well. After he prays over her the sick person eventually gets well and regains consciousness.

Him and his wife. His wife is sleeping. They are on their honeymoon and he just got up. It is the end of their honeymoon. She is still sleeping, he is kind of mad that he has to go to work. He tells her that he will see her after work and he just goes off to his work.

There is this kid who's mother wants him to play the violin, but he does not know how and so he goes to violin lessons and he is doing really poorly and he does not want to practice and his mother wants him to play in front of his family and he knows he cannot so he is faced with a dilemma. (**Q**) Frustrated and sad.

There is this girl and she is growing up at a farm and she just graduated from ninth grade and her mother wants her to stay on the farm and help her brother with the chores and the mother thinks eighth grade is far enough but the girl wants to go on for high school and make a better life for herself. (**Q**) She is feeling confused. She does not know whether to listen to herself or her mother. (**Q**) She realizes the education is far more important, so she goes off to high school in the big town.

this lady is the mother of this guy and he has been working, going by her house and stuff to visit her and he worked. She has been forgetting things, getting Alzheimer's and right now he told her he is thinking of putting her in an old folks' home and she is surprised and does not know what to say. (**Q)** He feels bad because he knows his mother doesn't want to go to a home but he know it's the best for her. (**Q)** She eventually goes off to the home and finds out it is not as bad as she thought it would be and knows that her son is doing the best for her.

There is this kid who who has to go into surgery and I don't know get like some important surgery like a cancer taken out and he is scared because he remembered his father had the exact same surgery and died, and so he is scared of what is going to happen, if he is going to die too. (**Q)** He goes through with the surgery and he does not die and he feels more reassured about the whole process in hospital and all that.

There is this young man who thinks that his town is being attacked by vampires so he sets out to the woods where everybody thinks they are and he finds a spot and he goes to sleep and as he is sleeping this vampire comes up and kills him.

There was this family, just a wife and a man, and they were living in an apartment, like in the 1940s. And the radiator, heater, it was broken and the wife was always telling the husband to fix it and he never does and one day he goes to work and she is still in bed and he goes to work and he comes home and is calling for her and he goes to her room and the radiator was spilling out carbon-monoxide and she died of carbon-monoxide poisoning. (Q) Very sad. He feels it is his fault because if he fixed the heater it would not have happened. She is feeling nothing.

Once upon a time there was a young boy who always dreamed of having his own violin until one day he got his wish and he was very happy. (**Q**) Then his father arrived from another country and bought him a violin.

There was a young girl who went to school five days a week because her mother kept telling her to get educated so she would not have to put up with the hard work in the farm like her father and herself. So she tried really hard and learned as much as she possibly could. She thought that if she worked really hard, she could achieve her goals and get a good job. (**Q**) She became successful.

A businessman was getting some advice from his mother regarding his relationship with his family. She kept telling him to organize his life. He took it very seriously and began thinking about what he could do next to follow her advice. After a long conversation he went home and began a new life.

There was a young man who was remembering the stories his father used to tell him. About being a doctor during the war. He started imagining what it would be like treating wounded men while they were about to die. He starts feeling the pain the patients went through while wounded. The young man went on and wrote a book about what it was like fighting in the war.

There was an old man who brought back to life people who had been murdered without a cause. He thought of the consequences that might have occurred if he brought back to life the wrong person. So he seldomly did it. He felt like if he was doing the right thing as if it was his job/obligation in order to seek justice. (**Q**) Until one day, he died himself.

There was a young woman who enjoyed tempting young men into having sexual relationships with her. One day I guess she was rejected and was left alone lying on her bed. She felt bad about herself and felt remorse and after the young man left she decided not to do it again.

The boy might be thinking that he needs to play the violin but he does not want to. Um maybe the boy is thinking about what song to play. And by the way that he is staring at the violin he might be thinking that he does not want to play but he has to play, another scenario might be that he does not know how to play but they expect him to play so he is sad. [How does it end?] If he knows how to play then probably he will play even if he does not want to and in the second scenario he might let someone know that he does not know how to play. [How did it begin?] Before probably the boy was happy and did not expect that he had to play the violin, and by the look on his face he got disappointed and he might look a little bit scared, too.

Um. It looks like family and the daughter appears to be going to school. Um the what looks to be the father, he is a farmer and he is working the fields, and the mother is just standing there she is probably watching the father work, and she is expecting a baby. The daughter looks to be sad in what way, that she is not being able to help out in the farm, but I think that she knows that she needs to go to school in order to get a better future. [What happened before?] Before probably they just started off their day and she got ready to go to school and the mother she must have walked with her just to tell her good-buy, and the mother looks like he is staring up in the sky, probably she is thinking about the future. [How does it turn out?] It turns out that the daughter, she goes to school, and the mother she goes back to the house and takes care of the house chores, and the father he just goes on taking care of the field.

Um it could be a mother and son, it looks like probably the mother had told the son something bad happened. And he looks very concerned and very sad. And the mother appears to well not care, but like she had to tell him something because she has her back towards him, it must have been like something very bad. [How does it turn out?] probably the son left and thought about what he did or what the mother told him and apologized to the mother given that it was something that he did, or he probably came back and just comforted her if it is something that just happened to her. (Th?) the mother must be thinking well I already told him what I have to say so I am at peace now, I don't

have to worry anymore, again the son must be thinking about ways to console her or how to say I am sorry if he did something wrong. (F?) The mother must be feeling very at peace, very at ease that she already said what she had to say and the son must be feeling puzzled, and sad, not angry , but very sad.

Um. It appears that the young man can be a doctor and he is thinking about the things that he can do when he grows up so he can be a surgeon. Or another scenario might be that the boy since he is holding a rifle, he is thinking about going to murder someone. And in the back of his mind he is seeing that person lying and having doctors perform surgery. [How does it turn out?] maybe the boy does not go out and murder the person that he intended to. [What happened before?] Um. Maybe the boy had a fight with this person and it was something very bad that he had to that he was thinking of committing murder (F?) the boy must be feeling, must be having second thoughts about murdering. He does look very angry, but at the same time he does look very afraid.

It looks like a father is giving a blessing to his son. Um maybe the son is asleep and the father just went in to tuck him in then is giving him his blessing. [How does it turn out?] um it turns out that he covers the boy up and lets him rest and he walks away and goes to rest himself. (F?) The boy sleeping must be feeling at peace, very restful. And the father is probably feeling very happy. (Th?) The father might be thinking how very proud he is of his son. And the boy is just asleep.

It looks like a couple where the wife has passed away and the husband is mourning the death of his wife. Maybe she was very sick because she was lying in bed. And he was there with her after he died. [How does it turn out?] It turns out that the husband buries her and again he ends up living alone and very sad. (F?) well the wife does not feel anything because she is dead, but the husband must be feeling very sad, extremely sad, and he must be thinking as to what his life would be without his wife.

20

There is a boy that is sad and um his violin is broken Before um he was playing the violin he broke a string and broke the other strings um. [How does it turn out?] he is a professional (Th?) I wish I learned how to tie the strings back on.

There is a girl who lives on a farm who loves to read and the dad is a plougher and the girl becomes a teacher and reads to him. [What happened before?] um. Um. Her friend doesn't like her um. Because she was playing with another girl. She is feeling sad (Th?) she is thinking I wish I had a friend.

The man is thinking about a divorce He was mad and he didn't like it. [How does it turn out?] he becomes a teacher. [What happened before?] the wife and the man fought a lot. (Th?) he's thinking um we should have a divorce.

There is a girl who wants to be a doctor she wants to help people she is feeling um. Sad. Because her dad had surgery. Um. She becomes a nurse um. Um. (Th?) I am sorry that my dad had surgery

There is a man who wants to be a doctor so he helps the man by saving his life. [What happened before?] his mom and dad died when he was 8. What is he thinking… Um… I wish my mom and dad were still here, that they were there. (F?) Um. He is feeling happy that he became a doctor.

Um. There is a man whose wife is dying of a disease he is feeling sad. He hoped that she would be there. [How does it turn out?] he is a doctor. Before he was a vet. (Th?) he is thinking that his mom and dad could be there. (F?) sad .

He can't play violin so he's mad. Maybe he doesn't want to. Mom told him to play violin so he sat and watched it for a half hour, then his mom was mad at him and beat him up.

These two women like the same man. She gets to spend more time with him because she's happy with life the way it is. The other one wants to go to school, further her education. Then one day, she comes back to the village and the lady is pregnant with the guy's baby and the end . . . the one with the books is thinking that she has to do the right thing.

This man is the lady's son and has just come home to tell her that Dad's dead. The father died in the hospital. She can't believe it, so she tells him she can't believe it. The end . . . a funeral. The woman is surprised and the man is angry because he did not have a good relationship with father and mad that he didn't get to talk to him before he's gone.

Wacky. He's getting appendix taken out . . . maybe it is like war wound, not sure. This guy looks like he's wearing a suit, two people in surgery like . . . don't know what he's doing, looks like a dream-type thing. He's probably just remembering about a story his father told him about the war and he's drafted. This guy is glad he went to war and got education and works in corporation now and doesn't have to go to war. Father tells him to go get educated because if he doesn't he'll have to go to war. So he's thinking about story his father told him how he got shot and had to go to surgery. So he's slacking off in school and if he fails, he has to go to war. So he's thinking about getting motivated to go back to school.

This is a guy putting his grandson to sleep. Spending time with him thinking about when he was younger and he's just putting his grandson to sleep. He's feeling good that he had a good life, looking at his off-spring, how beautiful he is . . . happy.

Guy is cheating on his wife. Guy and girl got hotel, he is supposed to be home for dinner with wife, but decides to go out and sleep with the woman. He sleeps with her and realized that he should be home. He is feeling disgusted, so he gets up and gets in his car and goes home.

Boy looking at instrument and wondering how to play. I think he is sad because he does not want to play it. Thinks to himself how to get out of it. (**Q**) Sitting there daydreaming. (**Q**) Parents will not make him play it after all.

Woman on parents' farmland, has opportunity to go to school but she knows her mom wants/needs her to help, but she would rather go to school. (**Q**) Sort of sad. (**Q**) Standing there looking toward school, but farmland keeps pulling her back from her goals. (**Q**) She will be going to school.

Man thinking to himself. He did something bad so he is thinking to himself how to tell his mother, he knows it will hurt her but he told her anyway. She is thinking how could he and how could he get out of it. She is feeling worried for him. (**Q**) He's feeling bad. (**Q**) He will resolve his problem his way. Mother will?

Boy is thinking about father being murdered by these two men. He is feeling on the revenge side, plans to kill them with this gun in the picture. (**Q**) He will get his revenge because his thoughts keep coming back about his father.

Father appears to be losing his son, very sick. He does not know what to do. Instead of medical treatment he decides to pray. He is feeling sad. In the end the son dies after all. (**Q**) Man having hand over son.

Man walked into his house, found wife murdered, does not know who to call because they will think it is him. And he is not happy or sad. Sort of in between, just shocked. Thinking to himself, "who did this?" Story will end unresolved.

What is that? He bought an instrument and then he learned how to play it. [How does it turn out?] He learned how to play it. (Th?) Where he got it, maybe for a present or something. (F?) Nothing.

She's coming home from school. And is walking next to a farm. [How does it turn out?] She goes home. (Th?) Maybe she wants to live on a farm (F?) Her books

Umm He came home from work and saw his grandma. [How does it turn out?] She got sick. (Th?) what disease she has. (F?) the chairs

They are operating. [What happened before?] He got shot. [How does it turn out?] it was successful. (Th?) If he is going to live. (F?) the knife.

He is sleeping. [What happened before?] He was tired. [How does it turn out?] That guy woke him up. (Th?) Maybe he has a dream. (F?) His parents.

She is lying down. [What happened before?] Her legs got hurt. [How does it turn out?] She walks again. (Th?) How her legs are. (F?) His bed.

Um. I'm not sure. There was once a boy. Who wanted to go outside and play His mom said you have to finish your homework first. And you type fast. Then he was marched up to his room to finish his homework and that is all. (Th?) he is thinking I wish I could finish my homework fast so I could go outside and play. (F?) Um. Feeling. Mad that he can't go play outside.

There is also a lady who lives on a plantation in New England. She had a mom and a dad and she also had a brother. The brother would always take care of the horse. And the girl would always get sad because she couldn't go to school until one day she snuck out early in the morning and went to school When she came back they were all worried about her and asking her where she went and she said she was at my friends and the dad asked why are you carrying books. [How does it turn out?] Um. Um turn out. That the girl is grounded because they found out she went to school. (Th?) um. That she never snuck out of the house and went to school.

There once was a man He went to his mom's and wanted to bake cookies. They didn't have any peanut butter for peanut butter cookies and the man wanted to go to the store. And then he went to the store, came back and bought too much peanut butter and then him and his mom got into an argument. And his mom was crying. And that is all. [How does it turn out?] um they say sorry to each other. (Th?) that he bought too much peanut butter (F?) that he never argued with his mom.

Wooah. There once was a boy who could see dead people. And he wanted to go round to see if he could see dead people in the cemetery. And he told his mom that he was going to his friend's house. And he went to the cemetery and saw dead people and he saw a ghost, was frightened and came back home. There he found 2 ghosts that chased him from the cemetery and killed his mother and that is all. (Th?) he is thinking that he never had these powers to see dead people. (F?) that he …just a sec. That he never went to the cemetery.

There once was a man who was demon possessed he saw a man Can you erase that , that he saw a man. He was with his son and was very mad and then put a curse on his son and that is all. [What happened before?] the man fell off a cliff died, and his ghost came back, and then he put the curse on his son. [How does it turn out?] that the boy turns into a ghost. (Th?) the man has company now.

There once was a woman and a man and the man went to work and when he came back the woman was shot and the window was broke and so he thought that a burglar came in and shot her. He reported it to the police then they found out the woman was drunk, threw the wine bottle out the window and shot herself, and then all night that man was crying. (Th?) That he would have never went to work because his wife got drunk a lot. (F?) That he can't believe his wife did that.

One day a boy named um… Jake , decided to play a violin. On accident, he… um… broke it … um,…, he was thinking to himself, why am I even playing this? He was thinking of this question. For an hour or two and looking at his violin. He asked his mother later on why he's playing the violin. His mother said Because good boys play the violin. The end (F?) Now he feels great.

One hot summer day a young lady named Sarah decided to visit her grandmother who lived near a vineyard. Sarah thought that if she visited her grandmother her grandmother would get well from a cold… Even though she didn't want to, she decided that she should. She felt weird because she hasn't seen her grandmother in four years so she felt guilty. Her grandmother was right by a tree where there was not too much sjade but a little Sarah decided she would buy some books from a bookstore for her grandmother to read. Her grandmother couldn't read too good so Sarah read them to her. A weekk later, Sarah left and her grandmother was well again. Thanks to Sarah, her grandmother can come visit Sarah and Sarah felt good.

One day an old lady named Julie stared outside in the window And the IRS came to look inside her house. They found a bill that was overdue for her house. Did I say her name?) Julie did not know what was happening so she kept looking outside the window. The IRS man named Bob didn't know how to tell Julie that her house might have to be taken away. A couple of days later Julie got a letter saying that the IRS might have to take away her house. Julie thought that she'll never be able to find another house again or a place to live. She felt scared, afraid and alone so she went over to the IRS and asked why they had to take away her house. They showed … They took her bill from her file from her house when Bob came over. They showed her the bill and when she was looking at it she started to laugh and the IRS man asked why are you laughing? Julie said, "This is my granddaughter's who passed away bill from her house." Then Bob felt glad that they didn't have to take away a house from Julie. The end.

Whoa… um … gosh… one day, a man named John had to get surgery so he could feel better again from a poisonous candy bar in his stomach. The doctors had to remove a certain nut tat was in the candy bar that was very poisonous and could make John die. While the doctors were getting ready for the surgery, John felt afraid that the doctors may make a mistake. He thought about his child life when he was a kid. He… John decided to think about positive stuff like when the doctors get the peanut out of his stomach he'll be all right. While he was thinking of this the light went out. Then he woke up with no pain in his stomach. Then he knew that God was with him and that made him feel good. The end.

One cold winter night a boy named Brian had a very bad cold… It was almost Christmas and all of his friends were having Christmas parties while Brian was stuck at home in his bed. No one knew how to cure this disease. He felt really alone and that he might die and that no one would come to visit him when he is about to die. One night when he went to sleep the priest came into his room and put his hand over Brian's head and asked God to cure Brian. The next morning he felt terrific and decided to go out and play. He didn't know what happened, but he knew that God was with him. The end.

One night, um… a man named Adam took his wife to the hospital. The doctors told him that his wife was already dead and the doctors couldn't do anything about it . Adam decided to go home and rest. Adam felt really lonely at the time. When Adam decided to go watch TV and get stuff off his mind, he saw a commercial for help for when people die. He thought this would help him. Instead he couldn't even pay the price, couldn't pay the doctor's bill. It also made him cry. He started to think what he could do but it was too hard for him to even think because he was so sad. Then he thought of something he decided to call his best friend, Mark. Mark gave him some advice and told him to spend the day with himself with Mark. After that day he felt OK about losing his wife and he told <Ark that he was the bestest friend he'd ever had. The end.

Hm… Trying to think. Once there was a young boy who wanted to play the violin and when he was listening he got real sleepy and started to go to sleep and he dropped his head on his violin and broke it. (F?) depressed (Th?) Umm. About how he's going to be a good musician [How does it turn out?] It turns out that he got a new one.

Hm… Hm… UM. One day a farmer girl went to church and when she got back she saw a guy stealing a horse and she felt bad because she didn't llock him up and they thought it was a thief but really it was her father (Th?) That someone's taking the horse because she didn't lock him up but it was actually her father [How does it turn out?] She finds out it was OK.

Um K. Um. Once there was an old lady and her husband died and she is feeling bad but she is thinking what if he went to heaven or hell? (F?) bad [How does it turn out?] She finally figures out that he's going to heaven

OK. I don't know what they are doing. Once there was a guy in the army and he got shot and so now he is in the hospital being stitched and he is thinking he is not going to live and he feels pain [How does it turn out?] that he lives (F?) pain

Um… the guy is an angel from heaven and he is trying to help the old lady because she can't see [What happened before?] She got hurt. It was light and she was looking up and it flashed real bad [How does it turn out?] That she gets healed (Th?) She's thinking that she won't be able to see the rest of her life (F?) He felt a hand on her eye. That's it. I think.

Um. A guy just woke up for the morning and he feels real drowsy and his eyes are tired and he feels sleepy and he thinks he should just lay in bed [What happened before?] He stayed up till 11:00 at night and that is it [How does it turn out?] it turns out that he feels better after he eats his breakfast

Um. He went to violin practice and he is looking over notes. [What happened before?] he did bad in violin practice. [How does it turn out?] good. (Th?) he is going to do good in violin practice. (F?) sad

They had been working on a farm for a very long time. And she is reading a book and thinking about how good it is going to turn out. And she is thinking it is going to turn out real good and nothing bad is going to happen. [What happened before?] she read a whole bunch of books. She is feeling sad. [How does it turn out?] bad.

He just came to his mom's house from work and he is thinking it is going to go bad. And it turns out good. and it turns out good. [What happened before?] um come from his house and he had a bad day at work. (F?) sad. (Th?) um work is not going to turn out good. [How does it turn out?] good.

Um. The guy had a heart attack and the people are working on him to save him. And the boy is thinking everything is going to turn out good. And it turns out good. (F?) sad.

The boy is sick and he cannot afford to go to the doctor's and he is feeling sad. [What happened before?] the boy was swimming in the very cold water and they are feeling very bad [How does it turn out?] the boy turns out to be OK. (Th?) he might not make it.

The person died and the guy is sad. He is feeling very sad. The thing that happened before he was she went to work and they got into a car accident and he is feeling very sad. [How does it turn out?] bad. (Th?) her parents are going to be mad at him.

In the beginning this guy was trying to play his violin, then middle: he has had problems with it and end: he is very angry and annoyed and frustrated and he is just staring down on his violin. He looks like he probably does not want to play it anymore, is sick of it.

This lady is just walked out of Sunday school and then middle: she is observing her surroundings and I guess the end: she is reminiscing and thinking about good old days and about memories that she had. She is feeling a little sad, but proud. Proud because of place where she was brought up, that is, the town. She is going to walk back to her house.

This guy is an Italian mobster and his mother just found out that he killed some one. Beginning: he killed some one. End: That his mother doesn't believe that her son did that. Mobster guy, ashamed, he is going to leave life of crime.

Psychotic picture. Beginning: this man was wounded in battle, civil war battle. Middle: he is brought to hospital and surgeons are trying to get bullet out of stomach. His brother is standing in front of mirror and is going out of his mind. the injured soldier is in pain and regrets getting into war. Surgeons are going to fix-up wound. He is going to be healthy, but will have a scar for life.

This is in Armenia and it's about 1915 and during genocide where this Turkish soldier just stabbed this innocent Armenian civilian and now in the middle: the boy is lying down in his bed, he is in pain but at the same time he is angry and his father is kneeling beside him. And he is praising him. He is praying for Jesus so that he will not die and his father is very sad. And at the end, his son gets well from the help of the Lord and he forms Armenian revolutionary organization.

Beginning: Wife of this man is dying from cancer, no in the beginning wife is diagnosed with it. Middle: She is suffering from disease and her husband is

emotionally suffering from her pain. He cannot bear to stand losing her and at the end she passes away and the husband goes insane.

He sat down and he was looking at the violin and wanting to play. [How does it turn out?] But his mother said no so he just sat there looking sad at the violin. (Th?) He is thinking that when he gets older he could be a violinist. (F?) he's feeling sad because his mom wouldn't let him play the violin.

She was a young girl whose father was a worker and her mother was pregnant they were very poor and she couldn't bare that her mom was sitting outside with her pains and aches she wanted her to go inside and have rest but her mom wanted to stay outside. [How does it turn out?] It turns out that her mom had her baby and she had to be the one that takes care of it. [What happened before?] They were lived out in the country, they were able to go to stores but her mom is pregnant so she can't. (Th?) She is thinking if it is a boy or a girl (F?) Sad.

It is a boy and a girl the mother would not look at the son the boy got mad at the mom and the mother would not let him get his job so she would not look at him. He is feeling mad and (Th?) They are thinking that if his mother is going to let him have the job or not. [How does it turn out?] where he gets his job.

They are doing surgery on a person and they are doing the surgery on the stomach they are thinking if they could do it they are feeling scared and it ends out that he dies. [What happened before?] someone shot him by the stomach so he had to go to the doctors.

It is of a girl that is going to die, the father cannot stand it he is looking at her he cannot go to sleep. He keeps looking at her and he wakes up in the morning he finds that she is dead and he is very, very, very sad. (Th?) They are thinking if she is going to heaven or hell and he is sad and it turns out that he dies. [What happened before?] she was really ill. She had to lay down and she just died.

It is of a girl who is laying down um. The boy, the man walked in and saw the breasts and he would not look at it so he is mad, and he is thinking what should I do? And he is mad. [How does it turn out?] Turns out that he gets her out of his house and he leaves. (Th?) he is thinking what am I going to do (F?) feeling mad.

This boy just got the violin as a present. He wanted something else. He is not ungrateful that he got this but still he wanted something else, maybe a flute. He will learn to play it but he will probably learn to play something else also and he will be better at the other thing. And it is his birthday that is why he got the gift. (Th?) How am I going to learn how to play this. He is sad that he did not get his flute, but it is not ungrateful sadness, I have to stress that it is just that he wanted something else.

It is in France at a vineyard. Seems as though there is some sort of a love triangle but that is not it these two are involved. She likes the beauty of the countryside but she longs for something else. Maybe she is just appreciating the beauty right now. He is a worker there but he is not just a worker, he is the lover of this woman. She is obviously a few months pregnant from him. And this girl over here she is a niece of the owner of the vineyard and she is just there to have some studies done, she needed to get away from the city. There is a sadness to this picture but I cannot put my fingers on it. It seems like they are all striving towards a goal. It is not a common goal but they all have their own path. It seems kind of bleak. The only one who has any hope is this pregnant woman. [How does it turn out?] This girl goes back to the city, back to her school. She is pretty much out of the picture. But these two are going to stay here. The baby is what is going to come out of this. The child is going to be the first person to leave this place of the people who actually live there. (F?) tired. (Th?) Well the girl with the books seems to be looking off in the distance maybe at another part of the vineyard. She is neutral. The man is just trying to get his work done. I think he is beckoning to try to get the woman to come over. The woman is just enjoying the sun She like I said I think is probably the only happy one there.

This woman is this man’s mother. She is completely out of it, she has Alzheimer’s probably she cannot remember anything. Her son is successful He left probably when he should have when he graduated from high school. he is a business man of some sorts but he is troubled by the fact that his mother cannot remember anything. [How does it turn out?] Well he he is a good looking guy he does not look like he has too much worry in his

life but he is distraught. Afterwards he is just going to realize that it just happens that he cannot do anything about it, but he is not going to put anger at himself or anyone else about his mother's condition. He is just going to think about the times before and he is going to have those, as cheesy as it sounds and he is going to have those and they will help him immensely. This is the last time he is ever going to see her.

OK. These men are doing an autopsy on this man this man on the table is related to this boy. Um. This boy killed the man on the table. Um. The man on the table is this boy's father I guess. He never really hurt or abused the boy, I think he is just insane, it is a really long time ago. The boy, I think he is just really really screwed up and just killed his father, It is really sad. There is really no reason why it should have happened. (F?) Well these two gentlemen up here are just doing their job. The only way that they are going to know what happened is when they are through with the autopsy. They really have no relation with the other two. The boy is completely neutral. [How does it turn out?] Uum the boy spends probably about I don't know, maybe ten years in a mental institution, maybe longer. He is young and he is smart. He probably made it seem as though his father were abusive, possibly to get time off. (Th?) The boy is not thinking anything. Um. These two gentlemen are well the guy with the knife is thinking scientifically, he is just trying to figure out how the guy died, what happened. The guy next to him is saddened it is maybe his second or third time at an autopsy.

These two are related also. The older man with his hand outstretched he is this man's father. The younger man is I think he is dead and this man is his father just wants to touch him, maybe see if he is breathing to put his hand up to his nose, to his mouth. [How does it turn out?] Well the father lives a couple more years with grief, but he lives more than that after the grief maybe subsides. He thinks a lot about how he could have outlived his son and how strange that is. But like the man and his mother I think he is going to make a realization that these things just happen but he is going to realize that these things just happen but they happen for a reason. I think the boy just died naturally nothing extreme, maybe a heart condition. I mean that is extreme, but nothing dangerous nothing

murderous. (F?) The old man is not crying I do not think he will for a long time, but he is saddened, empty (Th?) He really just wants to touch him one last time.

This is not a real person. This is a blow-up doll. This guy is just realizing how messed up he is. Um. He has probably has not had any human contact in a while, physical, loving human sexual contact and he is realizing how weird it is and the books on the table are about human interaction, how to make friends, stuff like that. (F?) He is ashamed but he is ashamed in front of himself, no one else knows. I do not think he even has sex with this thing, it is just sort of a companion. I do not think he is blaming anyone for the way it is but he does know that it is different that it is odd, that he should be interacting with people, that is why he is covering his eyes. He is not sick. He is not really sick. That is why he has art on his walls. He is just he is just a little messed up [How does it turn out?] It turns out well he is self aware now, there has probably been a long time in his life when he has not been self aware and he has just been happy in his ignorance and I think he is going to break out of this it is over with this . He is definitely going to move on.Not today, but soon. (Th?) He is tired. He is thinking that he wants to change. I think he has put it in his mind right now that he will change.

What's that? [It's up to you] You are writing that down? He's thinking what to do. He's wondering how to play [How to play?] yeah. [What is happening?] He is looking at it. [What happened before?] Probably he made it. [How does it turn out?] Good cause it looks good to him. (F?) I want to know how I can play with this, how to make this thing work.

This lady is walking from school and she is like almost about to collapse or something. And she wants , like she just came home from school. [What happened before?] Just walking from school. She was at school. [How does it turn out?] Turns out that the lady that's walking from school helps this other lady who is about to collapse. (Th?) How can I help this lady? (F?) Like Oh no, like can I help you or something. Or should I help her.

Um they are probably like no forget they are probably, no never mind, a relative came in that they didn't know about and they are like astonished, surprised. [What happened before?] They were just minding their own business until this guy just barged right in. (Th?) This can't be my brother, he died or something, I don't know. [How does it turn out?] Turns out that this wasn't his brother he was just trying to get money cause he looked like his brother. (F?) Like DK

Oh um, this guy is sitting around this table he got in an accident, something fell down on his arm and he is bleeding very quickly and they wanted to stop the bleeding very quickly and they wanted to get the stuff off of his skin that is getting all over the place to stop the bleeding. [How does it turn out?] Turns out that the guy that got hurt lives, he just lives. (F?) Before or after? [anytime] before, how can we help this guy, should I help him?

Alright this lady died, and the father, um the husband comes and says goodbuy for the last time. [What happened before?] well she came down with a bad sickness she became very ill and they couldn't figure out what was wrong with her. [How does it turn out?] She dies. Well they could bury her, no they did bury her, they went away and cried. (F?) feeling sad for the husband. The people who came to the funeral are sad, cause they

won't be able to see the lady again ever. (Th?) They are thinking that like that lady was a very nice lady, a very nice lady

Um the father got woke up and he got out of bed, yawning, he got dressed, and then went and kissed his wife goodbye and then he went to work (F?) He's tired. He's thinking I have a long day ahead of me.

This kid is getting his violin ready to play in the school orchestra for the Christmas play. He is thinking what it I screw up and he is nervous. He does well and he plays well and doesn't mess up.

This summer day and the whole family is out on the farm. The husband is working on the fields and the mom is just relaxing in the sun and the daughter is going to sit under a triee and read some books because it is a nice day. At the end of the day they sit at the dinner table and talk about how nice a day it was. (F?) They all feel joyful and in high spiritas because it is a nice clear day and they are all relaxing around the farm.

This man was a top executive in a company and the company went bankrupt and he is telling his mom. She is thinking about what he is going to do now. [How does it end?] (F?) The end is she talks to him about sending his resume to some new companies to get a new job. He feels kind of sad and angry that his business went out of business, She feels shocked and a little disappointed, I guess.

This guy is a detective and he was after a suspect and the suspect was fleeing from him and he caught up to him and the suspect pointed a weapon at him so he shot him in self defense and now they are doing an autopsy on him. (Th?) (F?) His is feeling satisfied that he caught the suspect and he is thinking how rough of a job it is because sometimes he has to take peoples lives.

This was an old married couple that has been married for 50 years and one day he found out his wife had cancer and she became really sick. In this picture he is leaning down to see how see his doing. Kind of rub her head and be affectionate with her because she is dying. In the end she passes away. He is thinking what is he going to do without his wife now that she is dead. He is extremely sad.

this is a newly married couple and his wife got extremely sick and they weren't sure what it was. They just thought it was a small flu, but it turned out to be something worse and

she ended up dying in their house. In this picture she just passed away and he is crying and very sad. He is feeling very sad and he is thinking about what he is going to do without her because she was the love of his life.

What's that a picture of? The boy is sitting there being sad and he is not playing his violin on his desk. [What happened before?] He was playing and now he is not [How does it turn out?] his mother might tell him to play it. (Th?) I don't want to play this. (F?) sad.

The guy is working in a field and the woman is like reading a book and this other woman is like standing by a tree and there is a horse, barns, um. [What happened before?] the girl was reading the book. [How does it turn out?] he stops reading the book. (Th?) um he wants to go home, the farmer does. (F?) um. The farmer is feeling sad, I mean tired.

The girl is looking out the window and the boy is looking at something. [What happened before?] the boy was talking to the girl. [How does it turn out?] That they maybe start talking again. (Th?) um. What am I going to do. (F?) this one feels sad both of them feel sad, actually

There is a boy on the bed he needs the hospital. Um. This is confusing. Um. [What happened before?] they were doing something and the boy got hurt. [How does it turn out?] he gets better. (Th?) oh no. the boy is saying oh, no in his mind that he got hurt. (F?) sad.

Another boy is hurt and this other boy is sitting by him and he is going to put his hand on his face or his forehead. [What happened before?] he was doing something and then he got hurt. [How does it turn out?] he gets better after a long time whatever was wrong with him. (Th?) um this is hard. Are you OK? (F?) sad

This man is crying. He is in a bedroom[What happened before?] um. He was really sad of something. [How does it turn out?] that he stopped crying (Th?) get better (F?) sad.

Once upon a time there was a boy that could not do homework and he was still really frustrated and he couldn't do it and then he practiced and practiced and still he couldn't do it and then he went to his mom and then to his dad and they teached him how to do his homework. The end. (Th?) That he can't do his homework but his mom and dad teached him and now he can do homework. (F?) um. He is feeling sad, frustrated and then at the end he is feeling all happy and calm

There is a girl that had to keep going to school and her mother and father and her live on a farm and her father is mean and her mother is lazy so the mother and father kind of ignores them, ignores her a lot she is trying to talk and the mother and father are not smart, too and she wanted to be smart and when she got older she got smart and her mother and father weren't. [What happened before?] um. They used to be nice parents and then they turned all mad and lazy. (Th?) she is thinking that what is going to happen to my parents when I am all smart and they are not (F?) she is feeling that her parents are probably dumb and she is going to be the only one in her family that is smart and she is feeling sad and she is going to be lonely

First there is a woman and it looks like her father her husband died then her son came into the room taking off his had and the woman feels sad thinking about what happened to their father. And then they call the police and call the person who shot him but they never did so then the mother and the son and every body else went to the funeral. (Th?) What is going to happen with no father or husband to help you. And wondering if they are going to find the person who shot him.

A boy is dreaming what is going to happen to his life and it looks like a man is going to cut him and there is another guy and when he gets old he is going to get cut by 2 people with a knife in the stomach and it looks like he is already an old man so he is thinking about how that is going to happen to him like a deja vue, and then he ran up stairs and he is probably going to have a nightmare about what is going to happen to him. [What happened before?] he was thinking what is going to happen in the future. (F?) scared and nervous

It looks like the son died and the father put his hand on his hand and he is down on his knees and crying about his son because he wanted him to be a grown man. Somebody called the ambulance but they didn't have enough money so he died, his son died. [How does it turn out?] it turns out that the father is going to kill himself. (Th?) he is thinking about why would he die by sickness (F?) he is sad

Looks like her wife died by getting like by doing drugs so the husband doesn't want to look at her because he is sad, he is crying a lot and he looks like he is about to run away. [How does it turn out?] it turns out that the wife is dead and the husband doesn't want to see her anymore because the wife died. (Th?) he is thinking why did she take drugs a lot, she could have stopped (F?) that he is crying and he is mad and sad.

Does he need a name? [It's up to you.] Billy wanted a violin for his birthday b/c all of his friends had one and he was jealous and he really wanted to learn. His birthday was in a few weeks and his parents were down in luck, they didn't have a lot of money. And Billy knew this so he was really sad b/c he wanted a violins and his friends were making fun of him b/c he didn't have a violin and his parents kept saying we don't' have any money, and Billy's b/day was in a couple of weeks Billy was so anxious and his birthday finally came and he started opening presents and he got almost everything wanted except the violin. His grandparents came over for dinner and brought one more present and he opened it and it was a violin and he was so happy. He ran outside to show his friends. They started playing and Billy was a natural. Billy joined an orchestra and is still playing today. (Th?) He is thinking about how fun it is going to be to play his violin. He is like daydreaming.

I got to think about this one. All right. Beth, a young college student in Kansas to find that her mother married a young strong handsome man. She lived on a farm and her mother and stepfather grew corn, cabbage, and carrots. Um, the week she came home her mother had become pregnant. She quit college and stayed home to help her mother b/c her mother couldn't help b/c she was pregnant, obviously, so one day when her and her stepfather were working on the farm he asked her if she wanted to see a beautiful place. They hiked up a hill and down towards a lake where there were deer and fish and um there was grass and trees everywhere. When they came home the mother was found with a bunch of surgeons being operated on and they found out later that the baby was born. (F?) The mom and the father were very like happy and everything, but the girl didn't really want the sister because she wanted the attention so she went back to college and the parents lived happily ever after. The girl passed college and moved out to CA where she married a man named Mike Collins. (Th?) They are thinking about how great it would be to move the mother and the stepfather out to CA so the whole family could be together.

Can't think. Um. All right Abraham Lincoln had just become the president when there were fights down in GA and there were lots of murders. One murderer came to a big mansion where many of Lincoln's supporters lived. Many tried to run and hide but there was not enough room in closets and under beds. Um. By the time the police came there were 2 people surviving. The house mate and the biggest supporter of all, Lincoln's vice president. I forgot his name. Should I make a name up? [It's up to you.] All right. Vice President John Michael Adams. Um. He later found out that Lincoln was assassinated by I think his name was John Balloon or something. The maid and John Adams were very devastated but knew they had to go on so the maid and John Adams went out to Washington DC to become the president of the United States. (Th?) They are thinking about how terrible all of the deaths were in the house and how sad it was that Lincoln died.

In Roswell New Mexico there has been reports about spirits that have been getting around and cutting people's stomachs open. The leader of the spirits is a young possessed child named Michael Okeery. The latest murder was the governor and his three children. The National Guard is being brought in and so are exorcists. Michael is planned to be caught at the lieutenant deputy's house tomorrow night. I don't know how to end it now. Exorcists will try to get the devil out of the child and then they will send the spirits away. This is a horrible one. I got to figure out how to end it. [What happens now?] The spirits are cutting open the governor and Micheel Okeery is in the front just thinking about the different spirits that he is leading. (F?) Michael is not feeling anything because he is possessed and doesn't feel any thing. And the governor is just thinking he is in a bad dream right now.

Once there was a young girl who went into a coma b/c of a car accident. The opposing car had t-boned her and knocked her head against the steering wheel. As the parents heard, they drove as quickly as they could to get to the accident site. When they got there, there were many paramedics. They called one doctor, he was supposed to be a miracle man over to there house. When the miracle man got to their house, he leaned over the bed and started doing some hand motions. As soon as you could say

what are you doing the girl was awake and fine. (Th?) The doctor's trying to get God's powers into him to heal the girl and the girl is just lying there motionless because she's just motionless and she is just knocked out. (F?) The doctor's feeling nervous and the girl can't feel anything.

Um. A man is coming home from work and is looking for his wife He's looked in the kitchen and he is looked into the bathroom and she has been murdered. He doesn't move but is in deep pain. He calls the police and the police come over and dust her fingerprints. Um. He has touched all over everything to find the weapon or how she died so he was arrested for the murder of his wife. (Th?) The guy is feeling deep pain because his wife has just been murdered and the girl is just motionless and not feeling anything.

What's happening now? [Yes] Reading [Make up a story] Um. [Any story A kid once ate too much and he felt nauseated and when he got back into the classroom he needed a story and he threw up all over the book (Th?) I want to get out of school because I don't feel good (F?) Pain.

Once this lady and her husband lived on a farm and she was a Christian and her husband wasn't and she had her Bibles and books under her pillows and couches because if her husband saw them he would like beat her or something [What happened before?] She tried to run away from her husband because he was chasing her because he found out about the books [How does it come out?] The wife is going to make the husband a Christian and they're going to buy lots of Bibles and books (Th?) That God is mad at them and the whole world around them and how big He is. (F?) God's protection over them.

This family was really poor and this guy went out for a job and when he got home his mother got mad and they got in a big fight and now they won't speak to each other again (Th?) They are thinking that she's thinking that um, why he got that job, and he's thinking should I get a new job. (F?) They're feeling sad.

The kid's father had to have a surgery and he was in the same room and he had to turn his head because they were cutting his dad open [How does it come out?] It is going to come out that Dad will be all right and they can be friends again (Th?) They're thinking that they are glad that the surgery went well (F?) They are feeling happy because, happy because Dad's alive.

This one lady got sick and no one could heal her and this one special doctor he had power like Jesus and he had power and they called him up and said can you heal her and he said "I don't know, let me check on her and he went over to her house and he put his hand over her face and um she awakened (Th?) They are thinking that they are lucky to have a

doctor like that (F?) They are feeling, they are feeling, they are feeling good that Jesus had time to make a great man like that.

This one guy, he woke up and he rubbed his eyes and he found that his wife was dead and he was so sad that he ran out and he sat on the porch and just sobbed (Th?) That he would take the place of his wife if he would have known (F?) pain

He was sitting down looking at the violin. Um. He thought it broke so he got sad. Then his friend came up and said I will help you out that is the end. (Th?) he was thinking that he would have to pay for it and get a new one. [What happened before?] he broke the violin when he was playing it.

She was looking at all the people that were working She said to herself I do not think they should be working they are not servants so she read the bible and it said something about there should be no servants working for other people because there is no other God but God who is in heaven so she went up to the boss and said you should quit your job because there is no other God. That is the end. [What happened before?] um she was going outside and she saw the people and she started thinking it shouldn't happen. (F?) she felt like she felt sad for the people that is how she was feeling like.

It was that he was talking to his mother one day and he said that you should live with me. They kept on arguing so they got in a fight and they did not really talk to each other um. They wanted to say I am sorry but they could not stand it they were feeling selfish. [What happened before?] he went to go see her and talk to her and see her and ask her if she wanted to live with him. (Th?) they were thinking that they should live with each other.

There was this guy that robbed the town so um some people wanted to kill the guy and some people did not so the people the wanted to kill him got to because there was more people that wanted it so the boy saw them and started crying and he wanted to tell the government about all the people killing the guy that robbed but he could not because he would have to pay to ride on an airplane. And he was very sad but then he felt better after a while. (Th?) he was thinking that they should stop hurting that man he didn't really mean to rob the town. [How does it turn out?] it turned out where the man died and he forgot about it and he felt better when he forgot about it.

One day this man went out and he looked at the sun too much and he turned blind and the doctor tried to cure it but it did not work. So his father prayed and prayed and then one

day the angel came down and touched him and healed him and the man's father felt better. That's it. (Th?) they were thinking the person was going to die with the blindness. (F?) They were feeling sadness .

One day this lady went out and was walking some robbers killed her and then her husband came and talked to make her live again but it did not really work The end and then she died the next day they found out she died the next day and he was really really sad. And he forgot about it after some days he got married again to another wife. That is it. (Th?) they were thinking that she would live but she didn't. (F?) they were feeling sadness at the beginning and then happiness at the ending part.

The little boy is looking at his violin. He seems a little puzzled so I guess he is trying to figure out what to do next; (**Q**) He was studying looking at violin trying to figure out what to do and lost in thought. (**Q**) like he is kind of lost at this moment but trying to discover how to figure out how to play violin, maybe he's depressed at moment. (**Q**) He will be okay, figure it out and be happy.

This lady was on a farm, looks like she was going to school or studying books. The other character is a guy with a horse in a field working without a shirt and a lady that is viewing what's going on; it is a clear sunshiny day, maybe out in the country in a rural area. She looks like she does not do any work in the field, like pretty dressed, just looking on just saying to herself that she hopes she does not have to do this as her job one day, that's why she is carrying her books to study; (**Q**) I think she'll continue to study go to school because I don't think she wants to do that work.

I see an elderly woman who looks like she is in a room, maybe she had a discussion with this younger guy; they came to a pause maybe both speechless, maybe they were having a great discussion and there was silence, with nothing being said at that moment, (**Q**) depression, grief, sadness, (**Q**) it will be a better time and them two get their sadness, grief together, they are not looking too happy right now.

looks like this is a gentleman that has been shot, hurt, injured; they have a doctor who is performing a surgery with a knife or blade; the young boy, maybe the guy that is having surgery, is in deep thought about the little one; there is a gun in the picture, maybe the guy was shot with it or trying to defend himself; (**Q**) I think he is hurt, feeling pain, the little boy is probably feeling happy for him.
(**Q**) I think he will be okay, survive;

In this picture we have an elderly lady who may be that she's on her dying bed or sleeping, and an older gentleman looks like he is praying for her, kneeling down on his knees, she looks to be asleep or unconscious; looks like they may be in some kind of a

dark isolated room. (**Q**) She is unconscious, he\ is in a state of praying, kneels down, first saying something.(**Q**) he may survive but she will still be bedridden or sick for the moment.

Looks like this man and woman spent the night together. And now he is up in the morning and is feeling sort of bad and angry for what he has done. He is thinking he should have not spend the night with her since he does not know her. And he is thinking that he needs to get out of there and go to work. She is still asleep but when she wakes up in about an hour she is going to be confused because he is not going to be there. She is going to be wondering where he is but also she is going to be sort of relieved that he has gone and that she does not have to deal with him. After all she does not know him either. The story ends with both of them going about their lives never seeing each other again.

Ok, well I think that he probably made a mistake in his violin and he is kind of bummed out but he is going to try again and he is going to do a good job. (Th?) that he can't do anything. [What happened before?] made a mistake playing the violin.

I think she's watching either a family member or a friend working in the field and she is writing a book and I think she's probably worried about this lady over here and I think she is going to get her book done and it is going to be perfect [What happened before?] I think that her family member or her friend who is working in the field got mad at her because she did something while he was working in the field and it distracted him. (Th?) that she could finish her book pretty soon and hoping that it will be a great success.

I think his mom just um I think his mom just just asked the boy a question and he was insulted from what she said but he knows that she didn't meant to hurt him and she just loves him and he is trying to decide if he should walk out or not. [What happens?] I think he is not going to walk out he is going to forgive her.

Um I think this guy is worried about his surgery and he is thinking what's going to happen and he is afraid he's worried [What happened before?] Hm he found out he had to have surgery. [How does it turn out?] Good. It's going to be successful.

I think this guy in bed is sick and his friend or family member is coming to see him and I'll just say it's the dad, the dad is going to touch his forehead because he knows he is very sick and he wants him to get better and he loves him. [How does it turn out?] I think he is going to get better and it's it's going to leave him with a sickness like he might be paralyzed. (Th?) I think he is thinking that he wishes he'll get better and that his other family members should be there. (F?) um sad

I think in this one his wife just died cause he is crying and he's feeling very sad and he wishes this didn't happen and that she was just sleeping and not dead and [How does it turn out?] I think that it'll turn out very sad but he'll know that she's going to heaven.

60

(Th?) Thinking that she'll thinking that he hopes that she'll get better and that she'll just not die. [What happened before?] she was in hm. I think she was probably in a I think she was in a car accident.
