# Supplementary material for: Integration Between Cerebral Hemispheres Contributes to Defense Mechanisms
Source: Front Psychol. 2020 Jul 7;11:1534. doi: 10.3389/fpsyg.2020.01534 (PMC7359856; doi:10.3389/fpsyg.2020.01534)
Supplement: DATA S2 — TAT stories from comparison group. [file Data_Sheet_2.PDF]

NOTE: Examiner comments in green. Q=general query. Th = what were they thinking? F = What were they feeling?

2

Reading up on how to play the violin. Thinking "I don't really want to do this." Is feeling very bored. The boy is bored and ready crush the violin over someone's head, like his instructor or something. The End.

Stand girl pretty still. They are thinking, I had better get out of this hot sun before my hair gets bleached blond. Woman ready to go over to the man sitting on horse to give him a really bad wedgie. I've gotten many of those. (**Q?**) They walk off hand in hand and eat pasta. (**FeelingQ?**)

Looks like Ricky Ricardo and Lucille O'Ball. Young man is thinking "I hope my date is ready soon." And the old lady is thinking "date better get down here soon or she is in trouble." Man is going to take her daughter out to dinner. Man feeling nervous. Man feeling pressed for time. (**Q**) He brings her daughter back at two am, four hours past curfew. He gets a waffle iron on his head. Grandpa had that happen to him.

It is something you do not see every day. Young man's father getting operation done. Young man is feeling nervous, hopes things are going to turn out all right. Man is not feeling anything because he is sedated. Doctor is concerned about losing watch in there. (How does it end?) Operation runs smoothly and everything turns out fine.

Psychiatrist, he has got her hypnotized. Is thinking that she has to tell him truth about how she is feeling and stuff. When she wakes up she is not going to feel as stressed as when she came in.

Guy is feeling sad about his wife who just passed away. She is going to end up cremated and in a while all her belongings are going to go to her boyfriend rather than him. (**Q**) He is going to remarry and buy himself a Bengotti. (**ThinkingQ?**)

4

Is that a book or I can't tell what that is… never mind. One kid is trying to figure out how to play a piece on his violin and he cannot figure it out and he is all angry at it. But finally he figures out how to do it.

When the girl comes home from school she finds that her father has already started working on the … plowing the field, and her mother is waiting on her to do her chores, like milking the cow for dinner (**Q**) So she gets done with her chores and then she eats dinner (**Q**) And she feels exhausted after her chores are done.

The old woman heard a noise, a frightening noise outside and so she called to her oldest son to check outside to see what it was and when he went outside he saw the house next to them was burning so he ran back inside and called the fire department.

The old man got shot in the stomach when he was in war, so they had to operate on him and his son came to visit him. And his son was scared that he would not make it but he did.

A magician was doing a magic trick and he needed some man from the audience to come up and help him with it, get hypnotized and when the man who did it awoke he was happy to be off stage.

One night the woman got very sick and the man was sad because he did not think that she would make it through the night but she did and so they were both happy at the end.

5

There is little boy is considering taking violin lessons. He is having hard time with the lessons and he is thinking about quitting but he is encouraged to keep doing it so finally comes time for his first recital and he is thinking about the first piece he is to do and he finally decides what his first piece is going to be (personally I don't know anything about violin music so I am guessing) and he is afraid of how he is going to perform but he is encouraged so he goes to his first recital and receives a standing ovation which he was very surprised at

Sun is coming up. People are ready to go out to work. They are getting ready to go to work or to school. Basically they all live out on a farm and since this is the old days they have to walk to school. Basically their work consists of hand plowing the field, probably even picking cotton too. The men usually are not ones going to school, woman are because men generally do all the work on the farm. The women also do all the cooking. Basically they live off the farm they only go into town for a few necessities which they cannot find or raise on the farm. I did not mention that they all live in one house, the whole family, Mother, father, aunts, uncles, grandmother and grandfather and all the kids. (**Q**) Basically at the end of the day everyone sits down to one big meal that has been prepared throughout the day and everyone talks about what happened during the day then after the meal everyone, right before they go to bed talks about and thinks about what tomorrow is going to bring. (**feelingQ**)

He is at his parent's house and he is not there wanting, he does not want to be there but due to the fact that his father is ill he has to be there and he is trying to figure out how to comfort his mother and he is also trying to figure out what they are going to do with his dad soon to be gone. Now I got to get creative cause I'm in limbo here. This is going to be a solution. As much as they do not want to, they sell the house and she goes to live with her sister. Of course I forgot the one part that causes it all, his dad dies. (**Q**) Sad afraid of the uncertainty.

5

I’m going to get overly creative on this one because there’s a situation in this picture that don’t make sense. They are doing surgery on a dying man, he has some disease in his intestinal area and it does not seem the man is going to make it but they are doing all that they can to try to help him but it does not seem he is doing to make it. He thinks he is having a hallucination because he does not think he can be dying yet but he sees this little boy dressed up in the suit which is supposedly his angel to take him home. (**Q**) Odd, surprised that he is actually dying he was not quite ready for death yet.

Why do all these have to be at one particular concept? Don’t write that down! This lady is on her deathbed and she is at the point of death. There is no in between or point of entering into it she is at the point of death. She sees this elderly gentleman with a glow on him and he has his hand over her like he is getting ready to take her somewhere. Almost like he is pulling her soul out of her head. (**Q**) She does not feel anything she is just kind of in a transient mode, no feel of happy, sad; I would not even want to say indifferent. There is no feeling at all. It ends with him not guiding her but actually taking her to wherever she is going in the afterlife. I am not good at horror stories and that is what these three in a row remind me of.

She has been sick for a number of days and he is either her it seems he might be her husband and he has just got done spending his last moments with her. He is suffering from traumatic shock because he just witnessed her die. He is now walking out of the room and letting the doctors do what they need to do to prepare her for taking her away.

Johnny's taking…taking violin lessons. He doesn't really like the violin. His mother is making him take it so he's just sitting there looking at it. He's going to wind up playing it when he is older. [anything else?] Nope. [how does he feel about all of this?] He doesn't like it. [And how does it end up?] He winds up playing for an orchestra when he's older. [And how does he feel about that?] He feels all right. [OK, anything else on that one?]

Nancy's going to college. She's waiting for the bus to pick her up, and she's…has…she lives on a farm. She has a pregnant mother, and her father's plowing the fields, and she wants to be a business woman if she can…if her parents can afford to go… for her to go to college. And she's scared to leave her…the farm, but she's going to be successful in the future. [anything else?] Nope.

The sheriff is …the sheriff's visiting… is about to tell an old woman that her son was murdered. And he doesn't want to tell her, but he has to. She has to find out. Then she'll find out sooner or later. He's going to tell her that they haven't found the murderer yet, and he's very, very sorry that this happened to her son. [And how does it end up?] They…she dies of a stroke the next day. [Anything else?] Nope.

Somebody's going to get cut up. It's a surgery from a war wound. And the boy in the picture is going to be that soldier. And he's going to survive, then go back to the frontlines, and wind up dying. [And how is he feeling?] He's feeling upset and in pain. [Anything else about this story?]

The grandfather is visiting his son, but he doesn't know his son's dead, and he's going to be very sad when he touches his forehead to find out. [How does the story end up?] The boy's dead. They give him a nice funeral, and the grandfather dies. [Anything else?] Nope. [Any other feelings that you see in there?]

A detective just found his wife murdered. [What else?] He's very, very sad. And he wants to find out who did it and arrest them. [What else?] That's it. [How does the story end up?] He doesn't find the guy. [And then what happens?] He…he goes into severe depression. [Anything else?]

One day this boy was feeling sad because he did not know what instrument to play so he goes into the orchestra room still looking sad and then he finds the violin and becomes happy. He goes to the orchestra teacher and he said he wanted that one. He brings it home and tries practicing with a scale. After a couple of tries he got it. Later on, the orchestra teacher gave him music like a song. He tried to play it but he was having a lot of trouble so he felt sad. After a while he practiced and practiced a lot and at the concert they have, he had to play solo and he did an excellent job. And that is the end.

One day in the country, there was this girl named Erin and she was very sad because she lost her mother and father when she was little and had no home or any money. One of the days she was roaming she came across the field for this farm. She saw the man picking up apples for his horse. And she looked across and saw a lady gazing at the sun. She went over to the lady still feeling sad, carrying her books and she asked the lady who the man was. She said "He lives here and takes over the farm." Erin went over and asked the man a question. She said "Do you have any room that I can stay in for a while? He said, well, I am going to ask you a lot of questions before you can come into my house. She answered all of them perfectly. He said, yes, you can stay in my house. Until you get the money you need, you can stay. After weeks and weeks, she got the money and bought a little cottage out in the country. The End

One day there was this man, he looks kind of lonely or he needs help with his house. He found this maid that was working in a different house and the boss was treating her very badly so she was starting to run away but the lonely man found her before she could and he said if you help me clean my house, I will pay you a good amount of money, so she said yes, and since he was very rich he had a big house and since he was all by himself he gave her her own room and paid her fifty dollars an hour. After about five years with her working in his house, she had enough money to buy a house and a car. So she said it is time for me to go now because I have enough money to buy a house and a car. So he says "Thank you for helping me fix my house. And good luck to you" She also says thank you

for being kind and considerate and helping her get her act together. So she moved to England and a nice fancy house. The end.

Oh God. I don't know how to tell this, can it be short, like really sort? One day there was a father and a son and while he was outside playing these two robbers came and took his father to their special hideout in town. Once the son found out where they were he was very very scared he had a scared look on his face the whole day. He heard a gunshot and they shot his father . The boy had a very sad look on his face and he was scared of those robbers that they might come and do the same to him (pause) Oh, my. I did not even see the gun in the picture! I thought I made this up but it is there and I thought I made up the club house but they are in a little club house

One day these two friends were playing outside Their names were Fred and Greg. They were playing basketball and all of a sudden, Greg fainted. Fred called 911 and he was, Greg was taken to the hospital. The next day, Fred went to visit Greg and he did every day because they were best friends. After about a month, Fred came in and he saw that Greg was feeling much better. That day he came home from the hospital, took his pills and then they both went outside to play basketball [In the picture] He is like waving and he is dead… or he is sleeping and his friend is saying like, Wake up.

One day this man woke up and he went into the next room to visit his daughter and all of a sudden he found his daughter dead lying right next to the bed he found a knife with blood on it and blood on her stomach. And he found a note saying that we are going to get you next time so he was very sad and afraid. He went and told the police and they figured it all out, they got the two murderers. The man recovered and he was living happily and safely

OK You want a story just from this one picture. Any time limit on this – I wish I could see what is in front of

This is a heavy-lidded young man, he may be reading – he may be have been reading for a long time I'd say he has been reading for a long time. You can tell he's been reading a long time by the placement of his hands, one on his jaw-line, near his ear, the other one on the other side of his face, near his temple and the fact that his elbows are also supporting his head and his hands so it is obvious that he has been reading for a long time. It is less obvious if he is really mentally absorbed by what he is reading or if he is just laboriously going through the task of reading. I lean toward the position that rather than being simply tired, that he is absorbed in the reading materials themselves and is deep in thought as to whatever plane or dimension these materials are taking him… [How does it turn out?] It turns out that he falls asleep, that his head drops down that his elbows and his hands lose their tension resistance and his head drops down on his chest and he gradually falls onto the book, if that is a book, it looks more like a binder. (Th?) it is difficult to tell what he is thinking because the only clues you have are the reading materials that he has and his face there are absolutely no details at all in the background or the foreground of the picture therefore I have no real clues as to whether he is in a garden, whether he is in school, whether he is in a church, whether he is in a park, whether he wants to be there . So knowing what he is thinking the only conclusion that I can absolutely draw is that he is very tired. (F?) he is feeling in addition to being tired. He is feeling a certain amount of self-absorption and loss of himself into whatever he is reading about. I don't think he is bored.

My goodness this is a busy one. Um. This is set in the Tuscany area of Italy. I say that never having been there but it appeals to me and I am judging that more on the older lady's dress and the man's dress than I am on the young woman's dress. And of course I am basing it on the geographical setting with the fields and the rolling hills and the water. I would prefer the sky to have some definition to it but it doesn't . I have a pretty good of what the young man is thinking and a pretty good idea of what the young woman is

thinking but I am not totally certain. (That is why court reporters have tape recorders for that very reason right there and that is not a criticism) of what the older woman is thinking or feeling. The younger man, obviously is getting ready to work the fields with a strong draft horse, probably a Percheron. The young man is used to farm work because he is so heavily muscled he is also more darkly complexioned than the young woman which could just be the shading in the picture or could be the effects of the sun. It is obviously a working farm judging from the buildings in the background. The farm the buildings, the land are the older woman's pride and heritage. The younger woman is dressed for going away to school, judging from the books it looks like she is leaving rather than coming and perhaps the young woman is leaving for the very first time. The older woman is not even watching her go. And the older woman's posture reflects to me pride, haughtiness, a sort of be gone with you attitude toward the younger woman. Is there hope for future reconciliation? I am taking you at your word here as a tester that there is no pass/fail because I believe deep ion my heart optimism is passing and pessimism is failure but as to the younger one reconciling or more likely the older woman taking back in or rather welcoming her back in, then I doubt it. If that is the wrong answer, then so be it. I don't believe that the younger man is rejecting the young woman. I think he is just accustomed to work. Now I just realized for the first time that I have not identified these people as mother son and daughter. I think that is likely. I think that is more likely than the young man and the young woman being husband and wife [What happened before?] What happened before I want to say that the young woman had spent her whole life in this existence she is now moving away from She appears to have some regrets about leaving and some reflections about leaving but she is leaving. I can't tell you from the clues that I seem to have here whether there was anything that happened that drove her away. I stick with my original view that the mother doesn't care whether the younger one comes back, but I don't have enough clues to say exactly why the younger woman is leaving except of course for the love of learning. [How does it turn out?] well I've already hinted at that and like I say, I could fail, but I don't' think the older woman really will feel the younger one is part of the older one's anymore. I doubt if the son will travel to see the young woman or not. He may, because he certainly doesn't dislike her but he is a hard working

farmer and I don't see any dairy cows that require daily attention so he ought to be able to get away some time to visit her unless the older one forbids it.

Lets see the woman looks like Bess Truman and the man looks somewhat like William Holden but it is hard to say, but I am not going to build my story around who they look like. So the man has his hat in his hands and he is looking down and he is certainly thinking and concerned and unsure of either what he just said or what he is going to do next, not that he is going to do it, but just unsure if that is the right thing to do given his situation with the older lady. The older lady is dressed simply but I don't think she is dressed as a housekeeper, by that I mean a servant, I think she has had a long association with the gentleman. But I am going to put them in the position that he has been a roomer or boarder at her house but she is a very independent woman obviously to run the boarding house although she had to start the boarding house after her husband's health failed but that was the thing to do in order to make money. So she has run this boarding house for a good many years and for the last several years, this gentleman has been not only a boarder, but a very good help to her in not so much running the business part of the house but in maintenance and as far as being steady and stable. So she has grown to depend on him not only for what he does around the boarding house, but emotionally too. Not a real emotional closeness, but an emotional tie nonetheless. And now he is moving on. Because he has found a better job somewhere else. And he knows that she needs him but he also knows he needs a better job. She is thinking my land after all these years what will it be like when he is gone. Can I still do it in so many ways like I used to and be so self-sufficient. So they are thinking their private separate thoughts and how will it turn out? They will keep in touch. He will come back, not as a boarder but as a visitor to check up on her. And he will touch base with whatever support structure she has left in the neighborhood to make sure she is all right so this is a turning point but I am very optimistic. (F?) Well I think I've said this but we can talk on it a little bit more I think she is a little bit in shock and wondering how can I cope and he is wondering am I really doing the right thing for her but I don't think he is wondering if he is doing the right thing for him and I don't think that is selfishness on his part I think it is just change in general so each of them is thinking about change.

Ok. My first question is this young man I think in the suit and tie, is he really in the operating room. First of all is this really an operating room. And I guess it is because I see tile, well I take it back because whereas the guy has a knife or tool in his hand of some sort, hm. Can't be it is not a hospital. Just doesn't look like a hospital operating room. They are obviously benign people working on him. That is to say that they mean to do him no harm, but the setting in fact the guy looks like Theodore Roosevelt, the surgeon does and the assistant looks like a German scientist that I have seen. But the room is too Spartan and doesn't have enough medical equipment in it to be a hospital room The patient appears to be wearing trousers but no shirt. And they are about to make an incision. What confusing me, well obviously everything confuses me because of the way I am rambling, is the young man in the coat and tie and white shirt. No one is looking at him. He doesn't appear to be part of the rest of the scene. so I am going to project that the patient is actually a cadaver and they are getting ready to do an autopsy to determine why I didn't intend to get so morbid. The cadaver killed the young man. And they are getting ready to do a vivisection which will of course include the brain of the cadaver Vivisections, believe it or not, are still legal in the united states under the United states code, but none have been done since the civil war. The youth in the foreground is not part of the scene yet dominates the scene. So I have to believe the actions of the two physicians have something to do with the life of the fellow in the suit. If that is true, the youth is not really thinking anything anymore, nor is the cadaver and I am not saying that just to get out of answering the question. The surgeon and his assistant are intent on their task as they ought to be since they are getting ready to cut open a body. I don't think they are thinking about the youth I think they are thinking about the incision and as to what will happen, I don't have any personal knowledge of vivisection but I have done a little reading on the extra y chromosome that some literature believes is present in the minds of those who commit criminal acts. Do I think they will find an extra y chromosome in the brain of this cadaver? I chose not to speculate and only will if you ask me another question (F?) well, I said that too, I said the 2 that are dead aren't feeling anything and I didn't mean that as a trick answer and the other 2 are just intent on cutting open the body.

I have seen that assistant in some scientific journal somewhere I don't know. But I doubt seriously that is Theodore Roosevelt so I don't think it is some historical enactment or something That is what I should have come up with I am going to quit talking [How does it turn out?] well I kind of headed at that too, since I have the pattern down, I don't think they will find the y chromosome, but I don't know, I really don't want to speculate…

I wish these weren't in black and white. Oh. That is interesting No. ok. We have a younger woman, nope, I believe it is a young man asleep on a divan, got a cushion from the divan and then another cushion for his head appears to be totally asleep not faking it or anything. Then we have an older man who's standing beside the bed or standing beside the divan but not totally standing he is also resting his right leg against the divan which could be a product of his age or of how he himself is feeling, perhaps tired. The young man has light on his face but doesn't appear to have any aura about him The older man could have an aura along the right side of his head and his body. But I discount that and say that it is not an aura but a just shading done by the artist to separate the body from the black wall. If these pictures were in color, it would have been an easier judgment on my part to make The older man is obviously reaching out to the younger man since I am discounting the aura I 'm also discounting anything like Vulcan mind –meld. You are obviously not a fan of star trek] But I do think that the older man is genuinely concerned with the younger man and wants to comfort him and let the young man know and the old man express how much he loves the young man. I don't believe he is trying to wake up the young man so the young man may never even feel the older man's touch but the old man will feel it and be glad that he did it. What happened before is obviously some kind of a prior relationship between the younger man and the older man. Family? I don't know. But obviously a bond there. Or the older man wouldn't come so close and wouldn't invade the young man's personal space. [How does it turn out?] I don't' believe the young man awakens, I don't mean that permanently, I just mean for the vignette. So I believe most of the experiencing, feeling and comforting is on the part of the older man. (Th?) The younger man is asleep. He is thinking pleasant, calm, peaceful thoughts. He

doesn’t appear to have any REM activity. The older man is thinking oh, how I love this boy and oh, how I want only the best for him.

The woman is ill. The man has been reading to her. He has read 3 books. He is tired, he is anguished, he is concerned. He doesn’t want to face what could happen, what may have already happened. I can’t tell from the position of the woman’s arms if she is sleeping or has expired. But I don’t believe things have been well for her. And the gentleman reading has experienced all of her pain and the frustration of trying to help and not being able to help. Obviously there was a prior relationship between them or he wouldn’t have been there reading 3 books. There is nothing to indicate that he is a medical personnel, or a religious man of any sort. And when I say a religious man, because I am here at fuller I have to be careful to include everyone. I mean, priest, rabbis, um. Ministers of the Scottish rites, other ministers after all Fuller is ecumenical. That is why I think this fellow has a relationship with her. He appears to have gray hair, she does not. That could indicate that is at least a generation in age between them, perhaps not. How does it turn out? I don’t know I want to be optimistic here and if she hasn’t recovered she will recover but it won’t be easy and it won’t be in a short period of time and it won’t be before the gentleman reads more books and encounters more desperation and frustration and remains tired. (F?) she is feeling weakness, and by that I mean physical weakness he is feeling sorrow, frustration, weariness, but also steadfastness. (Th?) he is thinking how long must this go on. She is thinking more of herself than she is of him, and he is thinking will I be able to endure this, by this I mean the physical discomfort she is going through.

This is funny… I think I know what he is doing because I used to play the same instrument. He says: "I cannot , I cannot play it, it is too hard." That is the middle… There comes the end" I did it, I did it. I did it. (F?) Sad like this like (whimpers) like [our dog] sometimes goes

This looks like more of a painting.. I am just examining it… This story takes place on a farm with a girl who is off to school but her mother says "no, you can't go until all the work is finished." That is the middle. Here is the end "how about I go to school now and I will finish the work after school.

Oh, my God, I don't know, I can't figure out this story. These people never seen… this boy never knew his mother and she never knew she had a son until one day he remembered where he used to live. He went in and he saw his mother looking out the window. He says "excuse me, I used to live here. What are you doing here?" And she says "I'm looking for my son." Here is the ending "Mom. It is me, your son."

I can only think of the whole story right now. This boy is remembering his faith His father died from war. He went to the doctor and the doctor had to get the bullet out but the father did not live so the boy said I will miss you, dad. That is the end

A man went to his house and he saw his wife laying in bed. She was not awake… she did not wake up she was dead and the man had to take her to the undertaker and measure her you know… here's the ending. They had her funeral on the same day they got married (F?) sad and a little happy

Can't do this one it has sexual harassment. I can't do sex. I can't A man got up and went to work and his wife got up late at night and she said what time is it and he said it is Saturday (F?) happy and funny.

I do not know. The boy is trying to learn how to play the violin and trying not to give up because he wants to grow up and be a violinist. And he does. (**Q**) Good that he is making like that he has accomplished something.

The mother's daughter is getting ready to go away with her boyfriend, and the mother is not really happy about it, and the daughter is really confused about what she should do and it is a hot summer day. And finally the daughter just decides to go and forgets about her mother. (**Q**) The mother is very angry with her daughter but the daughter is happy with her boyfriend.

His father died, and his mother is really in shock about it, and so is he. Then he has to go and move and take care of his mother because she cannot live by herself. And it is really hard because his mother is really old and is not expected to live much longer. (**Q**) Depressed and upset. (**Q)**The mother dies, and the son just goes on with his life.

It is gross. Two guys are cutting up another person for some unknown reason. And another younger boy is standing kind of out of the way not knowing what is going on and why they are doing this. And he just leaves there because he cannot stand hearing them laugh and talk about it. So he leaves and just goes away and does not think about it any more. (**Q**) Confused and upset because he does not understand what is doing on. (**Q**) He just goes on with his life and does not really worry about it anymore. He just lets it pass even though he does not want to.

A lady died, and a godly person is about is going to bless her so she can go up to Heaven and live on with her life. And she goes up to Heaven and has a really good time. (**Q**) Hope and sad and happiness. (**Q**) She did not she goes up to Heaven and has a good time and enjoys herself and enjoys living her life.

A lady just died, and the man is like really devastated about it. (**Q**) He cannot understand why she died. And he does not know what to do. (**Q**) Upset and devastated. (**Q**) The man reports the death and has a really nice funeral for her.

Once upon a time, there lived a boy who, um, didn't want to um…he…wait…who did want to play his um violin. And he um loved to play it like um, when… when the year was getting close, he got tired of playing it so um, he just stopped playing it, and he um, he had to say goodbye to it pretty soon, because he um, did not tell his teacher if he wanted to play it or not. So he, um, wrapped it up and gave it to his teacher. And the teacher said, "Why are you um…why are you giving me your viola?" And he said, "Because I…I Look at my practice chart." And…and she looked at it and she said, "You have not been playing have you?" And he said, "No, I mean, yes." "Then why is your sheet then why is your practice sheet blank?" I don't …" the boy…the little boy said, "I don't know, Teacher." And she told him, "It's OK if you didn't …it is OK if you did not practice. Iwill teach you how to do it if… because you are a new boy." And he said, "Yeah." The little boy said, "Yeah." And the teacher said, "Let us begin right now." And he said, "OK" And so they began to play because um the teacher promised him… the little boy that she would um teach him all the things that they did when he was not here. So they did. And he… and um, one day he said to the teacher, "Teacher, um is not it almost time for the concert? We had better um practice some more stuff like the new…like the new stuff that we have been doing because I'm going to move away pretty soon again because my dad is not doing his job so he has to go find a new job in our old town." And she said, and the teacher said, "OK, let us do it." And they started playing and stuff and ruining um the strings. The little boy was so happy that he did not want to go back to his hometown which was um Johnson City, New York. And he um told his dad "Why do not we just stay here and find a new job because I am beginning to like my new school?" And his dad said, "OK." And the mother said, "Why? I already…I…I…I…I started…I…I already started packing" And he said, the little boy said, "But mom, I have to practice, and there is a concert coming up." And the mom said, "Oh. So that is why you want to stay here, huh?" The little boy shook his head, "Yes." And he fell in love with his viola. And he… he wanted to repeat his school all 15 million times because he wanted, he liked his viola. And that's the end of the story.

Once upon… all right? Once upon a time there lived a woman who um liked to read books. And sometimes her mother and father made her do the garden tools so she did not

like it so she stayed on the wall and um gardened all day long until she got hot. And her mother kept on watching her to see if she was doing her job, and one day she saw her looking at the father. She saw the daughter looking at the father and not doing her work so she um took away her books, and she said, "If you do not work, if you don't work, you will not read." And she began to cry very loudly. And she loved to do the garden. And then she said, "Oh my, I better do the garden or else I will not see my books again. And that is what she did. And her father began to look at her, and her mother did. And she said…the mother said, "Why, you began all better. You began to do your garden work, and here is one book." So she began to gain her books by working in the garden. And she took care of her horses and swept the doorway and shoveled the ground if there was snow. And pretty soon she had one hundred, one hundred million books. And then the next day, she said, "Oh my. My mother must have given me a lot of books because I only started with two, and now I have one million books now." And pretty soon the little girl began to have um infinity books, one infinity and then two infinity and then three infinity. Then pretty soon she um since she had a really huge number which I cannot pronounce… I don't know what comes after athenity… fifteen million infinities, and she and she said to her mother, "Mother, why did you give me all these books? I only started out with two, and now I have a… a really huge number ." And the mother said, "Well you did your work. You did your play. You did everything I told you to do, and now I gave you all these books because I love you very much , and I am glad that you did that.: The father said… and the father said that she could take a break. "You are off your punishment. You did your work. You did your play. Now um you should go play with your friends." And she, the girl said, "No, I want to read. Could you help me with this word?" And the father said… and the father and mother said, "Yes. We can." And she began to list her friends because she… um they did not want her to read and so they began to go… move away from her because she was reading and they did not like girls um friends who read. And she um once she saw uh one of her friends moving. And she went upstairs to her room to cry because she knew that all the reading she had done has paid off because she is losing her friends because they did not want her to read, and now she is reading.

Did you lose me? [No, you are doing a great job]
And she said to her father and mother, “Mother and Father, I had better not stop reading or else all my friends will leave.” And she… and the mother called up all of her friends, and the mother said, “I cannot, I cannot hear what they are saying because they moved already.” And she said, “Oh, no,” and the daughter said, “Oh no, I lost all my friends.” And that’s the end. And that’s the end.

Once upon a time, there lived a mother who did not want to have a child. And finally she had a boy, but she kept on ignoring it, and when he grew… the little baby boy grew up, she still ignored it. And the son became a officer who did not come home very much so she began… the mother began to worry about him. And that once the father came home, and she said, Oh, you are finally home. Honey. Look what I did to this painting. And the boy said, “Why did you do that, my mother?” And she said, “Because I missed you so much that I could not even concentrate on my painting.” And he said, “Mother.” And she said, “Yes, son.” “You are under arrest. Sorry.” And she said, “Why am I “ “Because in Artist Land, you can’t you cannot ruin any pictures… paintings because, or photos or anything because you… because if you do then you are going to be under arrest.” And the mother said, “Ahhh. You cannot do that to your own mother.” And he said, “Yes, I can. It is in the rule book.” And she… and the mother said, “Fine. If…that is fine. Then put the handcuffs on me.” And he said, “Mother, why?” And she said, “Because you do not want me. Put … just put them on, OK?” And he said, “Oh I cannot do it. I will have to get another policeman to do it for me.” And she said, “Why?” “Because you are my own mother, and I cannot do that. It is in the rule book.” And she said, “Oh, that is funny. I never knew that it was in the rule book.” So they did it. So they read it and read it until she said… she looked up into her painting and said, “Oh, I cannot believe I did that to my painting.” And this other police officer came and talked to her, and she… and she got under arrested because she was crying and that was not allowed to in the rulebook either. So she got under arrested and put in a nuce room to calm her down, and she began to paint. And this… they let her go, and she began to paint…. Began to paint. So she would… wouldn’t um… she learned her lesson, never ruin the paintings because she would get under arrested again. And that is what she did. And he… she called up her son

and said, “Son, you had better go to a new job.” And he said, “What?” “Go to college and find a new job.” And he said, “What?” I can not do that, there’s no college around here.” And she said, Yes, there is. It’s in the rule book. Duh. There are lots of colleges in here, duh, duh, duh.” And he said, “Oh, I remember. There is a college around here somewhere, and I am going to Artist Land.” And she said… and the mother said, “You are already in Artist Land, Silly.” And she… and the son said, “Oh, yeah, I forgot.” And she found a nice cottage to live by the … cottage to live by the new um… casino which was a college. That is what they called it. And so they… and so they began to work and play. And the son began to go to the new college because he wanted to be an artist just like his mother. The end.

I can’t. I didn’t know what picture that is. [Well just… what do you think is happening in that picture?] I don’t know. Ih, yeah… I know. [go ahead]

There once was a boy who had a… who had lots of nightmares, and once he had a nightmare about his father, and then next he, next was, next to him that was working on him was him because he had lots of nightmares, and he was afraid that his father was dead so he ran into his dad’s room and um… and said, “Ah, finally. I thought that you were dead, Father.” And he said, “Gruh, I am not dead. Just grab my gun and put it away.” And he said, “Why?” “Because I am not going to go shopping or anything with that gun. It is being noxious to me. And he said, “It’s really… the dream is really, really, really true. The dream is really, really, really, really true. And he said to him. “Son, I feel a humpback on your back.” And he said, “A humpback, a humpback. I cannot see the humpback. Where is it? Where is it? And he said… the father said, “On your back, duh.” And that is where he got his first humpback, and he had lots of nightmares with that humpback because he knew it would move into his stomach, and he would have a nightmare that his father or mother or him would have a nightmare about that. And he said, “I had better get rid of this humpback. And I am going to go to the… the… the…” And he stopped for a second and said, “Oh, I forgot. It’s… I can’t go to the… the… the… the… the … the the the the… the… the cemetery.” And he said, um, “BB But with a friend, I can, I think.” And he… and he told his mother and father, “Bye, I have to go somewhere, I am going to go to my friend George’s house.” And he… they said, “All

right. Be back at 3:30." And he said, "Wowee! I get to go to the cemetery." Very softly. And he went to George's house, and said, "Let's go to the cemetery." And he said, and George said, "Oh, we cannot, I cannot." And he said, "Why not?" "Because…" And George said, " Because my Mother and Father would worry about me." And finally he spoke up, George said, "I am sorry, Friend, but I cannot, I cannot, I cannot." And… and George was afraid because he saw the humpback move onto his shorter shoulder. And he said, "All right. I'll go." And they went to the cemetery. And um Mag said, "Let us get rid of this humpback… that humpback and fast!" And he… and he said, "Ghost of the graveyard, take this humpback away." And he said, "OK." And the ghost said, "OK." And he said… the ghost said, "Another ghost is over there, and he is the one who takes humpbacks away. I am the one who takes warts away. And he said, "Ooh! Never mind. Bye." And he went to the ghost of the graveyard that takes humpbacks away. And he said, Ghost of graveyard that takes humps away… humpbacks away, take this hump out of my body." And the ghost said, "OK, but my sister is the one that takes humpbacks away." And he said, Oh, I'm getting sleepier. Oh." And he looked at his watch, and said, Oh, no! I have got to go fast." And his sister came early, and said, "OK, I'll take the hump… the humpback away. Whzzzz." And he looked at his watch and ran to his house and said goodbye to George. And George said bye to him, too, and he said… and they both went back home. The end.

Once upon a time, there lived a kid that loved to sleep, and once he had… he was dead, and this ghost came out and said, "Wohoo, wohoo." And he… and he didn't… and this kid never woke up. And he… the ghost said, "Wohoo, wohoo, wohoo, wohoo, wohoooooo.," And the kid finally got, and got spooked. And he laid back down dead… again! And the … and he… the… and he noticed that it was his ghost so that is why he was looking dead. The end. The end **(Q)** Oh, the kid was feeling kind of dead and scared. And the… and the ghost was feeling … feeling scared because that was the other kid's… the other kid's voice that was saying it to him. And he did not know…. The kid did not know that the ghost was him, and so he finally woke up and he said, "Get in my body, Ghost, or else I will kill you.": And the ghost said, "You cannot kill me. You cannot kill me. You cannot kill me. Try to kill me." And he said, "OK, I will take this gun" And

the… and he shot it. And the ghost tell me… and the ghost said, “You cannot kill me. See, you… you cannot kill me.” And it went right through. And the ghost said, “Oh! I cannot kill him. I have to kill him or else I will be in big troubles. You get in my body.” And the ghost said, “Ahh, OK, I will get in your body. All right. OK.” And he finally went into his body. The end.

There once was a boy who tried to wake up his mother. And the mother would not wake up so he started crying. And he said, “I am hungry.” I cannot make my food all alone.” And the mother said softly, “You can. Just use my recipes in the … oooh” “In the …” And the kid said, “In the what? Oh, yeah, I remember…” And the kid said, “Oh, yeah, I remember. In the dresser of her… of my mother’s.” And he went through his mother’s dresser and looked in the books and looked everywhere for the recipes. And he said, “Oh, I cannot find it anywhere!” And the mother said, “Look in my pillow.” And the kid said, “OK, I will. Give me your hand, Mother!” And he said it pretty loudly so everybody could hear it. And… And he said, Oops. Did I wake up everybody in this house?” And the mother said, “Yes, you did. Now go find anybody… your sister… your big sister and everybody else to help you cook.” And he says, “Why me? I cannot do anything. I cannot cook. I cannot.” And he said it pretty loudly so everybody could hear that the mother was dead. The end. And he said it pretty loudly so everybody could hear that he… that the mother was dead. There were no doctors and no others to help the mother to be alive again so they had to bury her. **(Q)** [How did they feel about that?] Sad. OK.

Feeling sad because he cannot play the violin. **(Q)** He starts crying because he cannot play the violin, then he sits down and once he tries it again he gets better at it [Anything else?] No. **(Q)** He starts playing it again and he got better at it

They are like out at the garden and there is like this girl coming home from school [any more?] They're like making like a garden **(Q)** They like go back to the barn and put everything away like my dad never does, he leaves it all for me…

That's like, this guy's like, this guy has on a tie and a coat and he has a hat in his hands and that is probably like his grandmother or his maid and she is looking out the window **(Q)** um Hey look, I got a fax machine. **(Q)** The guy goes outside and goes to the bank to pay the house fund but he does not have enough money to pay the House fund and the guy at the bank says if you do not pay your loans you will be bankrupt and they end up bankrupt.

(long pause, stares at picture) I can't think of one [How does it start?] [What are they doing?] The boy is like just looking like straight and there's this guy that's dead and there are two guys in the background, I cannot describe what their clothes look like **(Q)** can't tell **(Q)** umm, since the boy's dad is dead they had his grandfather watch the boy.

There is this, like, this like guy, I think, it's like a guy or a girl can't make it out and he is going to touch his head because the guy is probably sick **(Q)** I cannot think of it **(Q)** I still can't [Anything else?] No (looks tense)

Um this guy is like blocking his eyes from the light. The light is on and his wife is asleep and the light is on and it is like hurting his eyes **(Q)** Hm… oh, instead of the light is in his eyes, he is exhausted from probably being at a party too long or he got back from work **(Q)** Can't think of it (looks puzzled) **(Q)** Can't think of it.

I saw that picture yesterday. So I need to say how it got to this point and just lead from there? Well – To me it looks like a little boy who was interested in going to music class in school so his parents might have gone out and bought him this violin and it looks like he doesn't have a clue how to play it and it looks like he is thinking about what he is supposed to do with this violin. probably wondering if some day he will be able to make some kind of music out of it. just changing the subject a little bit, I got one of these when I was about his age so I know how much fun it is to try to figure it out. But (F?) confused probably wondering if he can actually play this violin, but right now he has no clue, wondering if he made the right decision if his parents actually got an instrument that he was wanting to play. [How does it turn out?] well if we base it on my real life. I ended up giving it up in a years time because I had to move but it could also turn out for the best for him, and he may learn how to play it and he may grow up to learn how to play it and play it in a symphony or something there are several ways it could go but to think positive he will make it a success story and he will do really well with the violin or with any other instrument that he may change his mind and try to play he may become a really good musician and possibly go into the field of teaching music.

Oh boy. Well the girl in the picture to me looks like somebody who is wanting to get away from the farm it looks like she has a school book under her arm or a book of some kind it looks like she is heading off to probably by her age a college or a university to me it looks like she is unhappy where she is at. So she wants to um whatever she is studying she wants to get this over with so she can move somewhere else. Now the hum I didn't like this picture too much yesterday when I had to rate it but it looks like a farm that needs a lot of attention because the guy is out there working pretty hard and then there is this other lady how is pregnant who is standing up there against a tree. There is not much else I can say about this picture because I can't think of a continuation on this one [What happened before?] To me it just looks like the girl holding the book in the picture is just miserable there because she wants to move away from the farm and try to make something better for herself or of herself. Maybe she just doesn't like the responsibilities that are needed to run the farm. [How does it turn out?] well hopefully she succeeded in

whatever she is out to do and does have the opportunity to in her eyes improve her life. And of course the lady who is in the picture who is pregnant of course she will have a kid and start the process all over again and raise the kid and maybe they will do something different in the process of how they will raise the kid…(Th?) Well the lady in the picture might be wondering if in fact this is her mom that if this is in fact her mom she might be wondering what she had done wrong to make her daughter want to run off and do something else or you know, want to move away from home (F?) Well the girl holding the book in the picture looks like she just wants to get out of there the guy in the picture I don't think he has any feelings, well if it is his daughter he might be a little upset but it doesn't look like he has any feelings. And the pregnant woman she just looks she looks like she might be stubborn, she looks like she might have her nose up in the air, either that it is because of the way she is leaning up against the tree it looks like she is resting her head up against it.

To me it looks like a mother and a son. This one I don't have a clue on. [What happened before?] before this picture it looks like they might have had a disagreement of some kind .because she is looking away from him. He looks like he may have said something to her to upset her by looking at the look on his face and he might be wondering what he can do to correct that. And it looks like he still has his coat on and he is holding his hat like he is not going to stay very long. [How does it turn out?] well hopefully, if they did have their differences they do work it out. (Th?) well he is probably thinking that he, he is probably wondering what he may have done wrong to upset her (F?) he has a look of hurt in his eyes and she has kind of like an amazing look in her face maybe wondering why or what…

Well this looks like it is back something during the civil war or even earlier. And the guy laying on the table obviously has something wrong and it looks to be dark outside, night time, because there is a lantern there because they are getting ready to cut him open, it looks like it is done without any anesthetic. So those guys apparently know what they are doing back there for that time. This other person doesn't quite, to me, fit into this picture just because it kind of resembles an earlier time to me, I don't remember seeing a suit like

this on anyone on a picture. [What happened before?] by the rifle standing there it looks like maybe he was shot in the abdomen cause they are slicing him up down there to possibly try to get the bullet out. [How does it turn out?] Well. If he is lucky he will survive. But in the days of early medicine it doesn't always seem to be that way. But hopefully he will. Cause I imagine he has a family. And he will want, you know, to be back with them. (Th?) well I don't think he is thinking a whole lot because hopefully they have him knocked out but the guy who looks like he is holding a lantern and the one doing the cutting are hoping that they can go in and retrieve whatever they can find in there. And will be successful with it and of course this other person I can't even associate anything with, he just doesn't fit for me. (F?) well the 2 people working on him are feeling hope and hopefully accomplishment as well… the guy on the table is probably praying to make it through it.

Well yesterday I was trying to decide whether this person in bed was either just resting from being ill or if this person was dead. And if the person over him was either the doctor or a minister giving him his last rites. [What happened before?] well something obviously happened to the person who is laying down. That got him into that position. [How does it turn out?] well if the person was just ill, and the doctor did what they were trying to do, then this person will go on and live hopefully a normal life again, a healthy one. And if it is the other way, where the person has died, then it is just up to the minister to do what they have learned to do to properly sent the person's spirit in the right direction, I guess. (Th?) well to me the person laying down is not thinking a whole lot of anything and the person over him is doing what they are trained to do and wanting to do it to the best of their ability. (F?) Well the person who is standing over the guy is he can be happy or he can be sad. Because he may have been someone who knew this person or who was close to him… and he could also be happy in the other circumstance as well because he is the one who has been chosen to look over the person in the bed.

I knew this one was going to be in here – I just had that feeling. Well they both look exhausted and he looks like he might have had a rough day at work and to me she just looks like she is just laying there asleep she might have had some plans for them. but in

the process she looks like she did fall asleep because he may have gotten home really late and it looks dark in the room and I don't think that he really has anything else on his mind but just to lay down and go to sleep. [How does it turn out?] well by the looks of this picture it looks to me like he is just going to oh, change into whatever he is going to sleep in and he is just going to go to bed and that will just be the end of it, cause since she is lying there asleep already, it looks like she has just given up hope waiting for him. (F?) well she is probably hurt to me, to him how I see him is just somebody who is home from a long day at work and is just overtired. He is rubbing his eyes…

The story that I came up with for that one is because I am going through a divorce right now.

This is a hard one because… um… So I start like before the story starts? [Up to you] It looks like he opened it up out of a package and he is wondering how am I supposed to use this thing. And he takes it over to his father and has his father show him how to play the instrument and that's how he learns how to play the strings (Th?) Um… (long pause). I guess he's thinking do I want to learn how to play this instrument or not, He looks unsure of himself. (F?) (long pause). He doesn't look happy or sad, he's in the middle…

Um This was also in those pictures… There is a mother standing around the farm while her husband is taking care of the farm and the daughter is going off to school she has her books in her hand And the daughter doesn't look like she's happy to be going off to school. She wants to stay around the farm. [Is there more? What happened before?] She gets up on a nice sunny day having to go to school while her dad's working the farm. [How does it turn out?] um. She winds up going to school (Th?) that it is good to have the crops that they are having and that it turned out to be nice the way they can take care of the crops in the field. (F?) happy that everything is going the way it should and the horses are nice strong and healthy and that they are able to keep up with the work in the field also.

Let's see. It looks like they were in an argument first thing in the morning and the mother doesn't want to hear anything from the husband and she is standing in the doorway looking at something outside while her husband is trying to talk to her. She is ignoring him, So he just goes about his own business and goes to work. (Th?) um. She doesn't want to hear what he has to say and he is upset with her because she won't listen to him. (F?) she is feeling aggravated because she doesn't want to hear what he has to say and he is upset.

I don't want to do a story on this one because I didn't like this one in that picture either. It looks like the guy is going to stab this other guy in the stomach unless it's a nail and this guy has a hammer. [What happened before?] They were enemies and it was 2 against one. And the one guy himself lost against the other 2. [How does it turn out?] that they

kill the one guy. (Th?) (long pause – shakes head). I DK I can't end the story in this one. I DK (F?) I don't think they are feeling any remorse.

The husband and wife lived together for a long time. One day the wife gets sick and she has been laying in bedfor a long time, let's see she went into a coma. Her husband comes to the side of the bed, wishing that she would soon wake up and they could live happily forever after. [How does it turn out?] He says all kinds of prayers he puts his hand on her head and talks to her and one day she just wakes up and they live happily ever after. (Th?) He is feeling lonely and helpless. And she can't think of anything until she comes around until she wakes up.

I should have saved that story for this one. Now you need a beforehand, right? (Um, hum) Ok the 2 of them are in a play together and the lady had fallen off of lets' say a horse and she wound up with a concussion on her head so she is laying in the bed helpless and the guy is very upset because there is nothing that she can actually do for her, just be there with her until she comes around… and he is wanting answers on what had happened to her because he had found her laying in a field [How does it turn out?] she wakes up about 5 months later with memory loss and the guy is there trying to run different things through her head to try to help her remember different things that happened before her accident (Th?) she is still confused, doesn't know what is going on she is trying so hard to remember but she can't and he is still just looking for answers trying to get her to remember things. (F?) he is relieved that she is still, that she is awake now, and she is trying to remember different things because she is starting to feel better than she did before.

Wow. Um … It might be a while. Um … I don't know. Um … Yeah. He's … It can't be this hard. I don't know. Ok.

Oh wow. This is bad. I think it has reached that time of day. Right now it might as well be an inkblot. Sad. I think. This is actually hurting my brain, trying to think. I don't know.

They are mourning a loss of a loved one. Either that or he told her some bad news. I don't get much simpler than that. If it were a movie, if it were set up as parts I could … All I can think of is they're saddened by bad news or loss. That's about it. Um … death. Really depressed, or, um … My brain does not want to agree with me right now. What else is new?

Other than looking like my nightmares? Um … Hmm. Um … depends if he is the one who shot … the guy who is being operated on or? I don't know. He is either remembering his, I would assume his wounded father, or he, um, shot him, or the guy on the table. Wow. He, um, the there are a couple scenarios, he, um … Ok. The boy lives on with the memory of the man he killed. Although, looking from this picture it is a long shot.

Um … this will be a short one, I know that The man is closing the eyes of his dead son. Yeah, that's what I got. Um … Hmm. I am trying to look at the picture for a clue. It's not happening. I don't know. He … got sick with a disease … I don't know. Grief.

Hmm … More death. This one actually looks like death. Um … This is going to seem a little cynical. The man is in mixed emotions after he killed the lying woman. Or that is the woman who was in bed, lying down, lying as she is in bed, not lying lying. I can't think of anything else. All I have been doing today is describing pictures I could be morbid. He uh … he decides to end his own life. I scare myself sometimes.

He just got the violin and for months, for months he has been asking his mama for the violin. And he's beautiful and dark brown, and he gets it and he unwraps it and it doesn't look a thing like the picture. He's just disappointed but he feels he doesn't want to be ungrateful. but he's just "well, it's not what I expected, but whatever" And so he accepts the violin begrudgingly. that's it.

They are a plains family. I'm going to guess, Missouri. She, the young lady is of course the heroine and she um, she, is a scholar of some sort, most likely a teacher. she is looking off into the distance, daydreaming and the I could create a hour long story after this picture alone, redirect. I don't want to waste tape space.... Alright, Rebecca has just come home from her schooling in St. Louis, Missouri. She's come back to her family's land in county Missouri and they pretty much just established it. She is in love with an Indian. She doesn't know how to tell her mom, who is pregnant with her fourteenth child. She doesn't know how to tell her mom because the Cherokee Indians are quite looked down upon, but she is very much in love with this native American, and she knows she has to confront her brother, and her brother is very very very loyal and protective of his sister, very proud of his sister for getting out of the rut. and She is going to be a teacher and that's something to be reckoned with. and her brother has been really upset lately because it has not rained and the wheat crop is in danger of being too low and so there is all kinds of conflicts right now and she doesn't know how to tell or when to tell her parents and brother that she is going to run off with this native American. she decides its best for the family, after pondering for a long time, that she just keep the relationship a secret and in the end three years later my great great grandmother was born, there you go.

"Arsenic and old Lace" that's what it reminds me of...Jane is a neighbor of Jacob and Jacob is a fluent attorney and a dear friend of June, June just lost her husband and she has a lot of property taxes she has to pay and she has a limited income, June's husband didn't leaver her with much. He was a traveling salesman his whole life. she stood by him though they just lost their eldest daughter to cancer and she had been taking care of her for the last two years and I forgot the name I gave the guy, that's so awful, so Jacob is trying to help her keep her property, and she's just saying how she should just sell this place. there's nothing left. and he's being supportive of her, but he knows he can help her if she would just stop being stubborn.

This is bizarre, um, this picture is of young Dr. Frankenstein. he's thinking, daydreaming, about the science of creating life out of death and he is remembering watching his grandfather operate on a man who is dying and being fascinated by it. little does he know that this young Mr. Frankenstein will soon grow up to invent the most hideous monster ever known to man (**Q**) How is he feeling, well, he's just pondering, he's just thinking about it. He's feeling a sense of yearning and…(**Q**) that was the end. He grows up to be young Frankenstein.

This gentlemen is trying to quit smoking and he's going under hypnosis so he visits this renowned hypnosis specialist and this hypnosis is putting him and this gentlemen is quite agreeable hypnosis and this is putting him under and he's in a deep deep state and the hypnosis is guiding him under hypnotherapy guiding and he doesn't feel anything on his journey to stop smoking and he's completely under hypnosis and he's unconscious. (**Q**) that was the end

This gentleman has decided sleep with a prostitute of the first time. He's angry with his life he's bored with his life and he's frustrated with his work in order to get even with his life, his work to do what he was taught never morally to do which is sleep with a prostitute. He was brought up to believe this was wrong but this is the only way to get revenge so he sleeps with her and he's finished and he's getting up and then he realizes "what have I done? this is horrible and I can't believe what I just did." and he feels frightened, frustrated, angry with himself, but he cannot undo what he did.

(pause) hm I hate telling people stories that I have to make up… [What's happening?] I DK It looks like he just doesn't want to play his violin. Or maybe he just can't play, doesn't know how. [What happened before?] um. (long pause) maybe it was like passed down to him because maybe someone else in his family played but he doesn't want to. [How does it turn out?] hm. He might keep it but tell them it is not the instrument he wants to play but he will still keep it to be passed down, I guess. (Th?) probably that he would rather be outside playing than playing an instrument (F?) boredom.

So they are all going to be pictures of the stuff I saw yesterday, hum? [that's right] Well, um, I guess the woman by the tree might be the mother and the other 2 are the children. Um. It looks like it took place a long time ago so it might be back when the women were just allowed to be getting an education and this family has always lived on this farm and the son has made the choice of staying on the farm and working and the daughter is going off to get an education. Um. So then it shows her leaving and he is out working. The mom looks, she doesn't look mad, she looks pretty happy so she is probably pretty proud of her daughter for wanting to go get an education. Hm. [How does it turn out?] I think the son would probably stay as a farmer and with the girl getting an education she would probably end up being a doctor and her mother, and it might end up with her taking care of her mother or something…(Th?) um. The mother is probably thinking about how proud she is of her children. The son is probably thinking about all of the work he has to get done on the fields and the animals and things. And the daughter is thinking about how she has to leave her family behind to go get an education…

Ok. Um. Well this might have started out as the mother saw possibly the grandfather and the son he had moved out before and he has his own family and he had to come back for his fathers' funeral and his mother is pretty upset and he is upset – I guess they don't really talk to each other they are upset and the grandmother is worried because she is thinking about what she wants to do since the husband isn't there and I guess after the funeral and everything the son may have the grandmother move in with his family so he could take care of her and so she wouldn't be by herself because she is probably thinking

that she is pretty alone and he is upset cause he lost his dad. [How does it turn out?] um I think she will end up moving in with them and it will a lot better for her because she will have her grandchildren to take care of and she won't be so alone and the son will have his mother there as well to take care of.

8. I didn't really understand this picture when I saw it yesterday. Oh, you are going to write that down. Are you allowed to help me? I'm serious I don't know who this guy is supposed to be in this picture [It's up to you] that is what confuses me because he looks like a lawyer or something and the picture behind him looks like an army hospital. Long pause [What happened before?] um. Hm. Am I allowed to take him completely out? [It's up to you] yeah but if he is in the picture don't you have to use him? [It's up to you.] [What happened before?] (sigh) Well maybe this guy in the suit was riding this nice little 57 Chevy and he saw this guy in the road who had been shot by some robber-type-people, and being the concerned citizen that he is he brought the guy to the hospital and now in this picture he is just kind of minding his own business and this doctor has an assistant who is trying to get the bullet out. And at the end everything is fine and this guy lives because of this guy in the suit and they become good friends. The end. (Th?) um well the guy in the suit is um just thinking, hoping the guy will be OK and even though he doesn't know the guy he is concerned the doctor and his assistant are probably thinking about the patient and the critical cutting that they have to do to get the bullet out and if they can get it out, and they guy on the table is pretty passed out so he is probably not thinking anything right now. (F?) um. I think the whole picture is hopeful for the guy. And concern for his life.

Hm. When this story starts, this young boy is diagnosed with cancer and he has a very short-lived life. He dies in bed at night, well he is sleeping that is the good news. And the parents invite a priest to come in to bless him. [How does it turn out?] um. Hm. I think it will turn out that the parents would remember the son but soon after they would try and have more children and hope that none of them have the same cancer disease that their first son did. (Th?) Well the priest is thinking about blessing the boy and sending him off to heaven and probably feeling a great deal of sympathy for the parents. He is probably

thinking about wanting to comfort them too, and the parents are thinking about how big of a loss this was a first child dying and by the end they are probably thinking about they are still thinking about him but at the same time they are thinking about having another child and thinking about being more cautious and thinking about whether this child would have the same problems as the first one.

This one I didn’t understand either. Are you not allowed to help me on these. I don’t know if I answered this one right yesterday. Um. There is a man and a woman and they get married and have their own house and after a few years the husband comes home from work, late and finds the wife murdered. Um. And here he is obviously very upset about it. And he probably stays pretty upset for a couple of years and feels all alone but after a while he meets a woman at work and he falls in love again and they get married and have children. (Th?) At the beginning they are thinking about how wonderful it is to be together and about starting a family and the husband here is thinking about who could have done this and why and he is pretty upset and angry and over the years all he can think about is her and how he didn’t’ get to have a very long life with her and by the end he isn’t thinking about the wife anymore he is thinking about how happy he is that he found someone new to love.

I DK what they are thinking. Or Feeling, (Q) He's looking at a story, I think, bc I can't really see it that good. [Keep going. What is happening in the picture?] I think he is reading his story. [What happened before?] I DK. [How does it turn out?] He's going to put away the book and take out another one. (Th?) I DK [What do you think he's thinking?] Thinking about what is in the story. (F?) He's feeling his head, he's feeling like this (gesture).

I DK a story about this picture. (Prompt) Doesn't look like any story bc I can't make a story out of this. [What is happening in the picture?] A girl is looking at something. I DK what it is but she is looking at something. [What do you think he's looking at?] The trees. [What happened before?] I DK, [Go ahead] I can't make it up bc it's sweating in here. [How does it turn out?] I DK. (Th?) That she likes the trees. (F?) Her book in her hand.

I DK what they are thinking. The man is holding his head, and the girl is not holding anything. [What happened before?] I DK. [Can you make something up?] I DK. [How does it turn out?] I DK (whining) (Th?) I DK. [Can you make something up?] NO. This is so hard. I have to finish 6 of them? Oh gosh (Th?) DK (F?) Already told you that. He is feeling his hand and the girl is not feeling anything.

DK. This is so hard. I DK what is happening. [What happened before?] DK [Can you make something up?] NO I can't This is so hard. (Th?) All I want to do is get… This is too hard. (F?) I can't do anything this is too hard.

I DK. I'm tired and I want to do Ballet. This is too boring. [OK. Take a break. Do some ballet.] after break …. [What is happening in the picture?] That man is about to touch that guy sleeping. [What happened before?] I DK. IDK [What will happen afterward?] He's going to touch him. (Th?) I think they are thinking that I DK. [What do you think?] That is what they are thinking. (F?) They are not feeling anything.

DK. [What do you think is happening in the picture?] DK My head hurts. DK. DK. [What happened before?] I DK [How does it end?] DK (F?) They are not feeling anything. And my head hurts. (Th?) I DK.

41

The boy is thinking, he is thinking what he is probably going to do with that thing **(Q)** he is probably going to paint it **(Q)** … **(Q)** … [anything else?] he is probably going to give it for a present.

(long pause) [start?] (AT) (Who's in it) two ladies and a man **(Q)** one is carrying books and **(Q)** sad **(Q)**

[Who are they?] A man and a woman [What's going on?]… [What are they doing or thinking?] (Gets tearful)

[Pause… Any ideas?] No.

[Pause… Any ideas on this one?] No.

[Too hard?]

44

I don't know his violin broke, he's thinking he has to fix it. (**Q?**) Sad that it broke and he's wondering how to fix it. (**Q?**)The outcome is that he fixes it and he's happy that it's fixed and he'll play it later on since it's fixed.

I don't know. What are you writing down? Are you writing this down? OK, the girl went to school and now she's back from school and she has some homework. (**??**)The outcome is that she finishes it and goes to work with her parents. (**Q?**) How she's going to get it done. (**Q?)**) They're feeling there is no feelings in that picture. (**Q?**)You can't tell. They have wondering minds. Hers is. How to get homework done.

Don't write, let me think. They're thinking about what they want for dinner and the outcome is that they eat the dinner they chose and then think about what they're going to do later on. (**Q?**)They're thinking in the picture. (**Q?**) No, their minds are just thinking. Their minds are wondering what they're going to eat.

It is kind of grotesque. The kid's mom is having surgery. The outcome is that the kid is happy and the mom is better. So there's hope in the picture. Hope and sadness. (**Q**) Mom is getting sick so she needed surgery and she got better. (**??**)Mom can't tell yes, because of the lipstick.

What were the directions again? OK, a person got sick and a priest came over to heal them. After the priest leaves, the person dies because he didn't get medical help instead he got religious help which didn't work anyway. (**Q?**) The priest is sad because he wants to heal but probably couldn't. (**Q**) How to heal him. (**Q?**) He is in the white light, the tunnel. So he can't feel anything since he is going to be dead.

He's like "whew" relieved. He slept heavy last night and he had too much caffeine so there's a sigh of relief of tiredness and the outcome: he goes to work. (**Q?**)He's thinking about the rest of the day. Work, eating, and things like that. (**Q?**) She's just sleeping there. She's not really doing anything to take part in the picture.

(long look – scanning) Um. [ideas?] unun [What happens?] He is looking at a violin. (**Q**) We wanted the violin. (**Q**) He has it, the violin (**Q**) He does not know how to play it. (**Q**) Like he cannot play it.

They are at a farm (**Q**) They went to the library (**Q**) Oh. They leave the farm (**Q**) Why do we have to leave the farm? (**Q**) Sad because they have to leave the farm [Anything else?] nope

(Shifting in chair) She is looking out the window (**Q**) She came into the room. (**Q**) She's going to leave the room. (**Q**) It's nice outside (**Q**) Happy [Else?] Nope

Um. A guy is getting cut in half (**Q**) He hurt them (**Q**) He dies. (**Q**) Um, we want to kill him because he killed some of our people (F?) Mad and sad.

She is sick (**Q**) She has a cold (**Q**) She gets better (**Q**) She is really sick (**Q**) Sad

She's dead. (**Q**) Some one killed her (**Q**) Um. They bury her (**Q**) That she is dead? (**Q**) Sad (**Q**) Yes.

Sad. They look sad. [OK, go ahead…] This, this person, this story is about a person who is sad. [OK, what else?] He, he does not know what note to play on his viola or violin. [and what else?] he has a it looks like he has a pencil in his mouth. **(Q)** About what note he should play and what song. **(Q)** by him being happy and playing his viola or violin. And, um, doing great at playing his violin or viola in front of a class. [anything else?] no [is that the end?] yeah. .

This is a story about a girl who lost her boyfriend, and there is a … in the background is a horse, a barn, and a hill, and a man, and a woman, and a horse. And the… and the … and the… story is about the man and the woman losing friendships. That's it. **(Q)** Um, at the end, they get … and then they get back to each other. **(Q)** good [anything else?] no. .

This is… this picture is about an… a story about a man and a old lady. The man is waiting for a taxi, and a woman is looking for a taxi. And the man and the woman are… the man is mad and the woman is sad. The story is about them waiting for a taxi. **(Q)** They… at the end they get a taxi. [anything else?] no [that's the whole story?] yeah. .

This is a story about a man and a… a man sleeping and a girl awake, and there is a gun. And the man… and this girl looks mad. The people who are… one of the people who is sleeping has a knife in his hand. **(Q)** At the end they get up and then they see that a gun was stolen from them. That's it. **(Q)** Sad because they knew the bad guy was coming and gone. [is that it?] Yeah. .

A woman faded out, and the man is trying to help her to stop fading. **(Q)** at the end they… the woman stops fading and gets up **(Q)** happy. The man is feeling happy, and the woman is feeling a little dizzy. [anything else?] no.

This picture is about a …a woman with a dog who is sleeping, and a man is covering his eyes. **(Q)** at the end, the man uncovers his eyes, and the woman wakes up. **(Q)** Sad, because they, lost, because they lost some of their jewels. [and what happened after that?] they, they call the cops. [anything else?] no [that's the whole story?] yeah. .

Basically it looks like he's bored out of his mind and doesn't want to play the violin. (Q) Him wanting him not wanting to play the violin, I mean I guess he's going to have to. (Q) Um bored, probably say depressed.

He looks like their working on their farm. (Q) And looks like the girl is going to read a few books um.. one is plowing the field um… and the other one I guess is just watching. (F?) I honestly I don't know. [Any guesses?] the guy is tired from working. (Q) Everyone going home after a hard day's work.

Looks like he's trying to talk to his mother, I guess. (Q) Looks like he's feeling a little frustrated. And I guess she's looking a little depressed as well, I guess, I don't know. (Q) I guess they just finish what they were going to do and just go their ways.

Um…Looks like they're going to be dissecting someone. Really that's all I can make out. [What do you think they're thinking?] Um…I can't tell. [What happens next?] Honestly I have no idea. (F?) Him, I can't make out, I guess the other one I guess is having a good time dissecting. [What would happen next?] I guess they would finish up with what they were doing and do what they want to.

Honestly I can' tell what it's supposed to be about. [What do you think is happening?] Looks like he's going to check up on eh the person in bed, it also looks like therapy. [What do you think they're thinking?]. Um…That the guy right know is going to get his problem solved and the one next to him is going to help him figure them out. [What happens next?]. I guess he fixes what his problems are then he leaves and the other guy helps the next person.

Um…Looks like he's crying over the woman or something. [What else?] I think he feels sad and after that I have no idea. [What is the woman feeling?] maybe sad or depressed, I have no idea. [What happens next?]. Um…I guess he leaves and they go their separate ways. [Anything else about this picture?] That's all I can make out.

All right. I was never very good at this. Mother is going to make is making him take violin lessons. He does not want to, but his mother is making him. So, in this picture, he is sitting looking at the violin his mother just bought him thinking, "What am I going to do with this thing? I am not going to take that class." But he finally he finally makes a resolve with himself and says, "I am going to do this. I might not like it, but I am going to do it because my mother wants me to. So, he takes the violin and bow to the local violin instructor that his mother has signed him up for. And he says, "I just want to say right now that I did not want to take this class, but I am doing this for my mother." And the instructor and the violin instructor says, "Fine." And so they start off the instructor starts off by saying, "I am going to teach you how to put your fingers on the violin to make the different sounds. And the instructor puts his own hands on his own violin put them into the finger formation for the for the chord A, and then he brings the bow across it and makes and makes the sound for the chord A. And then he tells Timothy, "Here, now you put your fingers the way I had them and make sure to press as hard as you can against the strings. It may be a little hard on your fingers at first, but it is what you have to do to make sure the sound is sharp, and it is not grating sounding." So, Timothy puts his fingers on the nick of the violin, the way the instructor had. He brings the bow up and he rakes it across the strings making the sound. He thinks to himself, "I cannot do this." The instructor says, wringing his ear, "That was I am not going to lie to you, that was poor, but that was your first try, and I would have to say, for your first try, that was good." And so, for the next three hours, Timothy and his instructor go over the different notes on a violin. And when the three hours are over, the instructor says, "Op, time is up. I guess I will see you tomorrow." Timothy says, "Already? I cannot believe it is over already." So he packs up his violin in the case, puts the bow in the case, closes the clasps. And walks out thinking, "Gosh, at first I didn't at first I did not want to do this, but now it is so fun." And he and he walks home proudly with the violin in its case in his hand. The end.

The girl in the in the foreground of the picture is on her way to school, thinking about what she is going to be doing. I do not have a name for her. I'll just call her "Girl A.: And Well, girl A is going to be walking to school where she does not know what she is

going to be doing today because the teacher did not tell them what they were going to be doing that day. The girl leaning against the tree, well, she appears pregnant so she is just kind of standing basking in the sunlight because it is a very sunny day out. The man in the picture is taking is taking the horse back down to its stable because he just got done riding it. That is a description of the picture. That is not a story. All right, another go at it. Well, the girl that I was talking about before, she is on her way to school, and she does not know what they are going to be doing that day, but she does know that it is it is going to have something to do with some sort of field trip, and she brought books with her in case she got bored. And let's see. She is walking to school along a dirt path that pretty much leads out away from their area and into the small town area nearby. And let's see. This is a lot harder than it looks, trust me. Who's going to be listening to this anyway? All right. All right. Dr. Warren, Dr, Lynn, this is very hard so if you are listening to this, try to come up with something a little easier. Pleeeeease. OK, back to what I was doing before. All right, she is walking to school not knowing what to expect except a field trip to God knows where. And the other woman in the picture, she's, well, as I said before, she is pregnant so she is just basking out in the sunlight kind of just enjoying enjoying that particular day because it is very sunny out that day. I was going to get to the guy in the picture before we stopped before. He is taking the horse in the picture back to back to the stable because he had been out riding for three hours, and he was tired, and the horse was tired. So, he was just going to take it back in. So I should really stop starting my sentences with "So." Well, as he is taking back the horse, back to its stable, he happens to come across a rabbit out in their field. So being not very bright, he proceeds to chase after the rabbit because it is munching on their crops. So, he is going to chase after it and try to catch it. Big mistake because while he is doing this, the horse decides to go on a little journey. And it ends itself up well, with its foot stuck in a in an improvised rabbit trap. It does not hurt it, but it has got it where it cannot move so it is kind of lays so it is kind of laying down with its foot in this trap kind of waiting for someone to come will come along and get it out of there. When the man realizes that his horse had gone missing, "Oh no, what have I done now?" So he starts looking for the horse. And, well, he comes across another rabbit in their field. And he chases that one for about fifteen minutes before he realizes, "I was looking for the horse

again and I got sidetracked." So, he gets out of their field, and he starts looking in the wooded area nearby their farm. There is a small wooded area by their farm, and he cuts across a nearby creek, and that is where he finds the horse. This trap this trap was in water. Stupid place for a trap for a rabbit, but well, this thing was lying down in the water. It was half in the water half out of the water with its foot stuck in this trap in the water. So he proceeds to pull apart the trap to get the horse's leg out. Of course by now, that leg of the horse's is pretty bruised up so he know it won't really be able to walk on it, run on it too much. He gets the leg out, brings the horse back up, and he starts leading it walking back to their house. He puts the horse into the stable, and he goes and tells his father that one of the horses got their foot stuck in a rabbit trap, and now has a bruised up foot, and so pretty well it is lame. It can still walk, but it is lame. It will not be able to run or do anything hard any more. I'm coming up with something. And well, the father says, "Well, since that horse will not be able to be used for working anymore, we are going to have to buy another one which that means that we will have to start doing some extra work around here so we can afford to buy a new horse. And so, the entire family works a lot harder for the rest of the year, and the following year they are able to buy themselves a new horse, and everything just kinda goes back into place. The end. (**FeelingsQ?**)

OK, this picture depicts an an older woman looking out a window and a man in a black in a black coat holding his hat looking down as if he is sad. This is a pretty hard picture, too, because there is not much going there is not enough going on in this scene. Well, the man is the man looks sad and has the black on because his mother just died. She was she was she is preceding her mother who is the one staring out the window. And he is sitting there thinking. "I cannot believe she died already and leaving Grandmother here and leaving Gram here by herself. I guess I will have to take care of her now." That was for effect, that. And So he leaves the room to go talk to his sister about how they are going to take care of Grandmother, and Grandmother is still standing out there kind of staring out the window thinking to herself, "How could this have happened to my to my little daughter? She was not even as old as I was, and she still managed to die. Damn polio." I can't believe I just said that. Well, in any case, the man and his and his sister

decide that she is going to be living she's going to live with them because they are still living together, his brother. They are brother and sister living together because the sister needed to move in because she was having housing trouble of her own. So these three lived so these so the two of them decide to have the grandmother live with them because she could not take care of herself because she was very old. And uh well, so they make all the arrangements and they move some of the grandmother's stuff in their home, and, you know, she has her rocking chair and all of her quilting things and, you know, just things like that. And she spends. She spends her days telling telling still telling her grandchildren stories despite the fact that they are older now. She still treats like they were twelve. And well, she they mange to get along with their lives despite the fact that their mother is no longer there. I would say, "The end," but I know I have to come up with more. Well, one day the grandmother drops her quilting and decides to get up and actually move around the house, you know, see what is all in the house because she still has not seen the their entire house yet. So she gets up, and she walks around, and she goes up to their attic. She manages to get up into the attic. And she is looking through a sorts of old photos of her daughter and her children, the ones that she is living with, in like their youth and things like that. And well, she reminisces about the things that they had done and where they had been in their lives. And well, she just lives out her life just kind of by herself. I mean she has her two grandchildren there, but, you know, she just has to keep herself busy. The end.

It's an interesting picture. It's a very interesting picture looks mildly, for lack of a better word disturbing. OK, first I'm going to try to give a description of the picture as best I can. In the foreground in the picture, there is it looks like a kid who has a cold because the end of his nose is mildly dark so he looks like he has a cold. He's wearing a black coat, white colored dress shirt with a striped tie. I am not sure what the colors are because it is a black and white picture. His hair is combed to the side, and he has kind of blank look on his face. And um and right next to him, also in the foreground, it looks like a rifle or a shotgun or something to that effect. And in in the a the fore not the the foreground in the background of the picture, it looks like there is a guy with like a scalpel in his hand that is about to open like a guy's stomach or something like that.

Like I said this is a very disturbing picture. And there's another guy standing right behind him like kind of looking over him. And behind them is a window so it's almost like a mad scientist's lab type deal kind of going on there like they're making the son of Frankenstein or something. That's I am trying to think of this. I gave my description, but I'm trying to think of what the story is here. OK Well, it would seem that between the boy in the foreground and the guys in the background, it looks like there is some kind of like a plate glass of window or something to that effect. So I am thinking that well, this boy or this guy or whatever he might be like is kind of standing outside as they do this operation like he was some kind of a like he was some kind of hunter because the gun is in the same plane as him. It's like out there with him. So I am thinking he is some kind of like a bounty hunter or something to that effect. And he had to hunt down this guy that the two men are operating on in the background. And he is thinking to himself, "Oh what I do to get paid?" So he is just kind of standing out there by himself thinking this. And, well, the two men inside standing over him, the one with the scalpel is saying to the one standing behind him he's saying, "This is the finest specimen that he has brought in yet. I cannot wait to find out to see what we will find inside of him." And the man behind him says, "You know, sometimes you are just sick." The other man says, "I know, but then that is my job now is it not?" And the man behind him says, "Yes, this is true. It is what we get paid for." The other man says, "Well, let's begin shall we?" And he places the scalpel against the man's stomach near his navel and begins to slowly cut upwards ending at the top of his sternum right right below his neck. He says, "Whew, we are going to need some kind of a saw to get through his sternum." So they walk off to get the to get a saw. And the other man goes, "I can't believe that they are going to saw this man in half just to see what is inside of him when they should already know." The young man walks off and well the end, because I can't really think of anything else that's I wouldn't want to go into detail on this picture being that I am a fairly morbid individual when I want to be. (**Q**) Well, the assassin, whatever you would want to call him, he's just kinda he's just kinda got the cool on the exterior but turbulent on the inside type thing going for him because, you know, it is his job to hunt people down, but that does not necessarily mean that he has to like it. So he has mixed feelings about what he does. Now, the one with the scalpel in

his had on the inside, he is kind of eager to find out what is going on inside this young man that they have hunted down. And the other one, he is kind of watching over the other man, and just kind of shaking his head, thinking, "Why, why does he have to do this?": So he has kind of got regrets that he is working for this guy basically. At least that's what I can see from this picture. I mean, it doesn't really give me much except four guys one of which is unconscious or dead and a rifle. There's enough going on in the picture, but it's kind of morbid so I wouldn't want to go into too great a detail. You wouldn't want me to go into too great a detail.

Well, a brief description. There is a man in a white dress shirt and a tie and black slacks laying on like some kind of a therapist's couch or a bed or something to that effect. And there is a man standing above him with his knee up on the bed or whatever it is. And he has his hand just hovering slightly above his face, and he is kind of looking down at him like some kind of like a psychic or one of those psychic healers or something like that. And well, the man lay the man laying down, who has his eyes closed by the way, is a man that has gone into see this psychic to see if he can make sense of some of the weird things that have been happening to him lately. He he's sometimes he will wake up like he had been through a deep sleep, and he will find himself some place that he does not recognize, sometimes wearing clothes that he does not recognize and, you know, just generally very odd feeling of "Where am I?" "Where have I been?" things like that. So, he goes into this psychic to see if he can make sense of this. Now, the psychic, he is standing over him. He is kind of doing that whole almost kind of a Jeddi mind trick type thing where he is trying to reach into his mind to see what is going on if there is someone else there or if there is some kind of split personality thing going on because that is what he would deduce from something like that happening. So he is standing over him, and he is reaching deeper and deeper into his mind. He comes across a section of this man's memory from what he guesses is his childhood, and he sees several painful memories, memories that, at least he believes, would be strong enough to drive a man into splitting in half just about. So he deduces that the man has split personalities or multiple personality disorder. How many personalities the man has is anyone's guess, but one at least has surfaced so he knows that he has to try and, for lack a better word,

trap this personality and keep it from taking him over and doing things to him doing things with him that he does not quite know what is going on. So, he is doing this, and he is just kind of doing his thing. And well, thanks to the psychic guy, the man laying down is able to recover and is only mildly bothered by the fact that he has other personalities living inside his head. They bother him every once in a while, but they do not fully come out and take control of it. The end.

OK, this one looks even more disturbing than that other one. OK, brief description of this picture. There is a woman laying on her back in kind of a bed, and she has got she is covered up up to her chest. And there is a man standing up with his back to her kind of kind of covering his eyes and his nose with his arm. He looks kinda guilty like he has done something wrong. And to the to what would be the left of the man, there is a small kind of an end table type deal with three books on it and a lamp. And then beside that there is a chair, and right above the foot of the bed, there is a picture of like, I don't know what you would call it one of those Spanish churches that you will find like down in New Mexico and things like that. And well, I really don't want to come up with a story behind this picture because it because it would sound very very sick, and I don't want to portray that picture for you two, for you three actually. So, let me think I could tell you what's going on in the picture, but I don't want to put a story behind it 'cause I do have a sense of morals, you know. Let me think. How many "umms" and "you" and "let me thinks" do you have on there? That seems to be about all I have so far what I have is majority of what I've said. Sooo Well, I'll just go ahead and say it. It looks like the man and the girl who is laying on this bed, for lack of a better word and because I'm just me, had wild and crazy sex, and well, now he is going to get up and leave and feel guilty about it for the next three weeks. And she is still asleep, so he is just kind of getting up and leaving before she wakes up. He is feeling an immense amount of guilt, remorse, and sadness because, I mean I mean, I can't really see much features of his face, but, I mean, he has his arm he's got his arm covering his eyes and most of his nose, and he does not look like he is smiling, let's just say. And the girl, she is asleep, and she is fairly content at the moment so that is what is going on there. That is all I can come up with on that picture. I don't want to go into any deeper detail.

I saw this one earlier… Now what am I supposed to do? I m not very good with making up stories… I'm not that creative with it… He looks pretty sad or disappointed maybe because he couldn't learn to play the instrument that he is looking at and he wanted to. [What happened before?] Maybe he attempted to play it and he just wasn't able to do it or learn fast enough for himself or he just wasn't satisfied with how long it was taking or whatever. [How does it turn out?] Um. Maybe in time he does eventually learn how to play that and becomes very good at it. (Th?) (silence) looks like he is thinking at that moment why can't I play this instrument.

(silence) um do I have to. I'm not a good story teller, I can't, I just don't know where to even begin or how to even start it. I wasn't good in school with public speaking or anything either…[What's on the card?] I can't tell. Um. My mind is blank on this. I just … [What's happening there?] I can't make any sense of it. What's happening here? This lady standing here all distraught. maybe a lost love or something. and this lady standing over here in the middle of a field for no apparent reason, pregnant, it looks like and this man standing here beside a horse in a field, looks like he's either been plowing the field or he is going to. … [What happened before?] (shakes head) [It's OK to make it up] Um… I have no clue like I said my mind is blank with some of this stuff I just can't think of anything [How does it turn out?] [silence] [shakes head] I don't know There is nothing here for it. (Th?) DK. (F?) She looks like she is a little upset or sad, she looks like she is happy and like basking in the sun, and he looks hot and tired.

OK. It looks like these two either shared some bad news or were arguing prior to the picture. Maybe right now they are thinking over what was said or done or whatever. They both kind of look kind of saddened or distraught over the situation. [How does it turn out?] I'd have to see the sequel. Um. Ah. I'm sure they end up talking it out with each other and basically sorting out whatever the problem might have been.

Hm. I don't know what in the world this is all about. In this picture it looks like there is a rifle here and apparently the guy's been shot and they are trying to remove the bullet surgically. I don't know what this person has to do with it [How does it turn out?] Well I think he survives it…the bullet is removed and it heals. I like happy endings. (Th?) Uh, well. What ever this person has to do with it they look pretty worried and upset in the facial expressions the doctors seem to be concentrating on removing the bullet.

(Shakes head) (silence) hmmm… (sigh) I don't know… I can't think of a story to put with this picture… [What's happening there?] I can't even tell. She looks like she's asleep, and he looks like he is getting ready to put his hand on her forehead or something. [What happened before?] Hm. Maybe she is not sleeping maybe she died and he was closing her eyelids or something [How does it turn out?] that is a tough one… um. I guess he is saddened by it and he is, but he deals with it. (Th?) I don't know

Um. [What's happening there?] Well evidently, not something good because he looks like he is really upset or saddened by whatever it was. [What happened before?] Hm. Uh. I don't' know maybe he was at work and he comes home and she is lying in bed maybe she had a heart attack or something and he found her that way and that is why he is sad and upset over the situation [How does it turn out?] I don't know (Th?) I don't even know.

It is this boy's birthday and he gets a violin for his birthday and it looks like he is concentrating on how it would work or how he could play it and before that this boy said to his mom or dad "I really want a violin for my birthday" but maybe he just likes music and this was a surprise but that is not likely. It ends probably where the boy is sent to a class where he learns to play the violin. And he is happy that he learned to do it because I know that it is hard to look at something and think huh, how do you do it and if you do not learn how to do it, it is a waste of time and money. [F?] It looks like he does not know what to do with the violin, maybe he does not know what a violin is, maybe he is trying to figure out which strings to do . He might have just gotten it and not know how to play it yet.

I cannot think of anything for this one. Well, all I can see is it looks like that guy's pants are just about to fall off [What happened before?] I'm not sure. I don't know because it doesn't give me a clue. [What are they thinking, feeling?] Looking at all three. That guy back there, I can't even see the expression on his face. I don't know what the characters are feeling [How does it end?] I am not sure how it will end at all.

It looks like these people are at a funeral or something. The guy looks like he has got on a tie or a tuxedo or something, not sure what he is touching. The lady looks like she is looking out of the window. And I am not sure about anything else how it will end or what made it happen [F, T?] I'm not sure what they are thinking because I can't even see the full picture. there would be something back here or something

Not sure what they are doing It looks like they have a knife or something and there is a gun leaning up in the corner or something and the back looks like Charlie McCarthy It probably ends where the guy dies, they stab him or… um, no, it is not a hospital [F? T?] Probably mad or pretty sad or something [What happened before?] not sure

OK. It looks like the person in bed is getting hypnotized or something I am not sure if he is sleeping or something but I don't know how it ends and I don't know how any of the characters are feeling in these pictures

It looks like this man's wife died or maybe that man just woke up and he is wiping his eyes because you know how people are tired and they wake up and stretch and wipe.their eyes, but then the wash cloth takes care of the rest of it [What else about the story] I'm not sure [How does it end?] I'm not sure if it really I'm not sure how it would end

This character took out a violin and is upset that he does not know how to play the violin. And he is thinking and plugging his ears. And this is the cloth that wrapped the violin, that is all [that is the end of the story?] Yes. [OK] And the bow is made up out of horse hair.

This story is western. This…it has western horses, a farm, a barn, some trees, a cowgirl, a cowboy, and a farmer's wife. And they are trying to hitch up a horse, a horse to plow both fields. And one of … and the boy has his shirt off because it is a hot day [And how are they feeling?] Happy [How does it end?] That um soon the other two ladies will uh change into shorts. And a sh.. a shorts, a skirt, and a t-shirt. The end

The grandmother is thinking "why is it snowing? The the man is upset, holding a chair, actually holding his hat. And he is leaning against the wall in the house, in the mansion, in a mansion, in the living room, in a mansion. [and how does this story go?] Um that they are not talking to each other. They are just staring at stuff. [And how does it end?] That they (sigh) will go eat for lunch. Eat out of their dining room. The end.

This boy remembers that his father … and he remembers that someone sliced his body open. And someone shot him, and he is lying on a bed with the covers on. And there are two doctors and a bookshelf. [Th? F?] They are feeling the intestines, the liver, the brain. And he is not liking, the boy is not liking it. The end.

This, the girl went to sleep. And she, and the guy fell asleep on a on a throne, on a um king, queen-size bed. And the man is trying to wake her up, because she dozed. She was dozing. And somehow he couldn't wake her up and she had a heart attack. [Th? F?] That she died. [How is he feeling about that?] Sad, That's what happened to my grandmother … before she died. That's the story The end.

57

This one is that another girl died, and they are using a chair at the end of the bed to support it. No, not… to support the bed from not falling. The guy is wiping the tears off his eyes because he is crying. And the wife is dead. And she is in the bed, and she is unconscious. The end [That's how the story ends?] Yes.

OK. It is not going to be a good story. Once upon a time, the end. **(Q)** Once upon a time Christopher broke his violin, I'm thinking. He broke his violin, he got so mad because he broke his violin that he punched his sister on the nose and they took his sister to the emergency room and he went into detention. And then he looked at his violin after he got home from school and he decided to break it with the sledgehammer, he got so mad. And then he threw the pieces in the trashcan and he ran away. **(Q)** He was thinking how sad, how sorry he was for his sister that he punched her because he got mad because the violin broke.

Once upon a time three hundred and eighty-five years ago there is this girl named Melissa and every day she would go to school and come home and one day her farm was burnt to the ground. She saw her mother and father and they were in the field crying because their house was burned down by the government. She got so mad at the government that she decided to talk to the president of the United States. **(Q)** Well she is thinking about her house burning down and that she got mad she was thinking about getting mad at the government **(Q)** They are feeling sad because their house got burned down and that is all I can tell you.

Once upon a time there is this old lady and this vampire that she met in Transylvania and then one night the vampire decided to bite her neck and to turn her into a vampire. And the next morning she woke up. She noticed that she was a vampire, too. She got so mad at the vampire that she met in Transylvania that she also tried to bite his own but she bit his neck and made a very powerful vampire and he got so mad that he decided to fly back to Transylvania and he decided to get the whole team of vampires to go to get the old lady. **(Q)** The old lady is thinking about when she was twenty about when she met that vampire, she was thinking about that time when he bit her neck. **(Q)** They are feeling like, the vampire is at least feeling thirsty, she is probably feeling mad, she is probably feeling sad because I can see her mouth.

OK. Ten years ago there was this little boy and two ghosts and the ghosts always chased him around when he was ninety-nine and then he died and three years ago the ghosts

decided to play a trick, but instead the ghosts got caught in their own trap. And then when he was ninety-nine he died they wanted him to be alive and so they decided to go into his body, but the dumbest thing was that they got squirted by water because it was not the real him. He was still alive in New York. **(Q)** They are thinking about how weird and dumb about how we are so crazy we knew he was still alive somewhere **(Q)** they were feeling ghostly, you know all tingly. The ghosts were feeling tingly.

OK. What is that guy (up to you) is ninety-one years ago, this nineteen year old boy died in a car accident, then one year his father went up and touched his face and ate him to bits. But there is one thing when he ate the nineteen old boy, he turned into the nineteen year old boy and all this Mexican stuff. The nineteen year old boy speaks Mexican. **(Q)** they are thinking about the father is thinking his son to bits and the reason that he ate his son to bits is because he got mad because his son died in a car accident. That's it **(Q)** The father is feeling sad because his son died, that is why he ate him to bits.

OK This twenty-nine year-old man was crying because one day he came home from work and they said that his wife had cancer she might die without further notice. But one day he woke up and it turned out the doctor said she was not having cancer, I thought she was not having cancer. I thought she had cancer but it turned out she was just having problems with her leg. **(Q)** he was thinking sad thoughts because he thought his wife was going to die without further notice but it turned out she was just having leg problems. And it turned out that she was lying, she just wanted you, Jack, to come home. **(Q)** sad, mad, angry that she died. And there is one thing. She still had leg problems.

Well lets see, looks like he is sitting there thinking about playing the violin and he is really bored and I guess it ends that he ends up playing it anyway. (**Q**) Just starts with him sitting there thinking about playing the violin. I was never any good at making up stories.

Okay, I guess this could be a story about a woman going off to school, or a girl going off to school. She is looking back at her family while they are plowing. She leaves everyone else to do the hard work. (**Q**) She looks depressed. I do not know about what but she looks like she is looking at the woman over here in the corner who looks kind of pregnant or something.

Okay, well this one I guess the woman is standing there looking at what is going on in the street and I guess it looks like the man is staring at a stain on the carpet or something. He is looking down real intently. (**Q?**) I don't know, I had a carpet cleaning business for a while and that's just what came to mind. And it ends with him cleaning the stain on the carpet. (**feelingQ?**)

Well, I guess this looks like a picture of a young man that accidentally shot his friend and somebody in the background doing surgery to get the bullet out and I don't know everything turned out fine and he lived. (**Q**) That he regrets what he did. He has bags under his eyes so he looks like he has been up a while thinking about it.

Well guess this one looks like an older man is kneeling on the bed and is getting ready to grab the person's face and scare him half to death. (**Q**) I guess it ends by the guy waking up and screaming. (**beforeQ?**)

I'd have to say this one looks like a man getting up in the morning and not quite awake yet, brushing the sleep out of his eyes, trying not to wake his wife. I guess then he goes off to work and leaves his wife at home in bed asleep. (**feelingQ?**) (**beforeQ?**)
